# Soil-derived microbiota induces T regulatory cells and protect against mouse colitis, metabolic disease, and sepsis

**DOI:** 10.1080/19490976.2026.2675089

**Published:** 2026-05-24

**Authors:** Edyta A. Szurek, Vu L. Ngo, Hirohito Abo, Anna Cebula, Benoit Chassaing, Rex A. Howard, Michael Hart, Shohei Hori, Casey T. Weaver, Andrew T. Gewirtz, Leszek Ignatowicz, Timothy L. Denning, Michal P. Kuczma

**Affiliations:** a Center for Translational Immunology, Georgia State University Institute for Biomedical Sciences, Atlanta, GA, USA; b Center for Inflammation, Immunity & Infection, Georgia State University Institute for Biomedical Sciences, Atlanta, GA, USA; c Microbiome-Host Interactions, Institut Pasteur, Université Paris Cité, INSERM U1306, CNRS UMR6047, Paris, France; d Mucosal Microbiota in Chronic Inflammatory Diseases, INSERM U1016, CNRS UMR 8104, Université Paris Cité, Paris, France; e Division of Animal Resources, Georgia State University, Atlanta, GA, USA; f Laboratory of Immunology and Microbiology, Graduate School of Pharmaceutical Sciences, The University of Tokyo, Tokyo, Japan; g Department of Pathology, Heersink School of Medicine, University of Alabama at Birmingham, Birmingham, AL, USA

**Keywords:** Environmental microbiota, microbiota–host interactions, tolerance, anti-inflammatory immunity

## Abstract

Recent years have highlighted the profound influence of the gut microbiota not only on local immunity but also on systemic host physiology. However, the translational potential of findings from preclinical animal models remains limited, in part owing to their microbiomes shaped in the absence of natural environmental cues. To address this gap, we developed a scalable, cost-effective, and reproducible preclinical model of environmental exposure (ENV), in which local soil-derived microbiota colonize laboratory mice. Sustained colonization with environmental microbes, particularly Gram-negative bacteria, was associated with a shift toward local and systemic anti-inflammatory immune responses, supporting the expansion of regulatory T cells (Tregs) and IL-10⁺ innate and adaptive populations. These changes confer protection against colitis, obesity, diabetes, and sepsis, ultimately extending both health span and lifespan. Thus, natural microbial exposure lays the foundation for future studies into microbiota‒host interactions, therapeutic resistance, and the development of more physiologically relevant models for human disease.

## Graphical abstract

**Figure:**
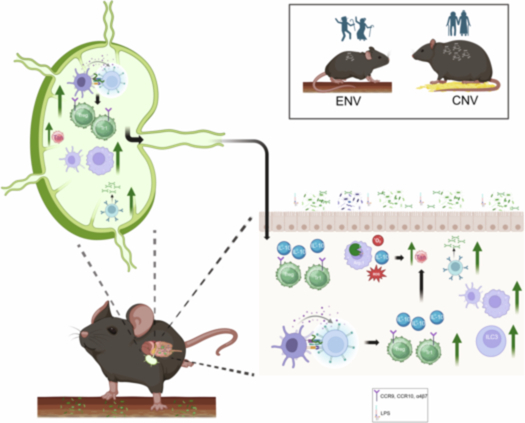

The environmental microbiota promotes local and systemic anti-inflammatory immune modulation, characterized by increased frequencies of regulatory T cells (Treg), type 1 regulatory T cells (Tr1), IL-10-producing innate immune cells, and group 3 innate lymphoid cells (ILC3). Innate immune cells presenting environmental microbial antigens activate adaptive cells in the mesenteric lymph nodes (mLN), inducing the expression of gut-homing receptors (CCR9, CCR10, and α4β7) on T cells and enhancing their intestinal migration. ENV-associated macrophages efficiently process lipopolysaccharide (LPS) derived from Gram-negative bacteria, supporting CD4 T cell survival and promoting their IL-10 production, which suppresses LPS-induced systemic endotoxemia. By reducing overall inflammation, ENV-exposed hosts exhibit improved glycemic control, enhanced resistance to obesity, and prolonged survival.

## Introduction

Laboratory mice used for studying physiology, immunology, and modeling human diseases ^1-3^ are housed in air-filtered cages with sterilized bedding, food, and water, along with routine testing to maintain specific pathogen-free (SPF) status. While these conditions enhance reproducibility and reduce infections, they create an unnaturally sterile environment that diverges from typical human microbial exposure. Since biomedical research ultimately aims to generate insights translatable to human health, SPF housing imposes limitations that call for alternative approaches. Recent models incorporating natural environmental exposures – such as wild-caught mice, pet store mice, “rewilded” lab mice, “wildlings,” and wild mouse microbiota-reconstituted (WildR) mice – have revealed how microbiota shape immunity and disease susceptibility, highlighting their translational potential.^4-13^ Unfortunately, these models are difficult to replicate owing to genetic, microbial, and logistical variability. Wild and pet store mice are genetically undefined and carry unknown infection histories. Rewilding is done using large outdoor enclosures; “wildlings” involve complex embryo transfers; and WildR mice need labor-intensive germ-free colonization. To overcome these challenges, we developed a scalable, cost-effective preclinical model – termed ENV (environmental exposure) – to study host-microbiota interactions under naturalistic conditions (Figure S1A). In this system, wild-type and lymphopenic mice (TCRα^k/o^) remain healthy for over 12 months when housed in cages filled with local dirt.

Exposure to live dirt microbes, particularly Gram-negative bacteria, triggered robust intestinal and systemic anti-inflammatory responses, promoting the expansion of CD4⁺Foxp3⁺ regulatory T cells (Tregs), CD4⁺Foxp3⁻IL-10⁺ Tr1 cells, and tolerogenic innate populations (e.g., CD11b⁺ cells expressing IL-10 or arginase-1 (Arg-1)). It also increased IgA-producing B cells, which collectively reduced intestinal inflammation and colitis, improved metabolic outcomes by preventing excessive weight gain and diabetes, and enhanced resistance to sepsis. Importantly, ENV mice lived longer and healthier lives, demonstrating that environmental microbial exposure supports longevity and counteracts inflammaging. Our findings make an important contribution to efforts aimed at integrating more natural animal models, which are essential for fully understanding human diseases. This approach enables the study of immune biomarkers predictive of aging, chronic inflammation, and metabolic disorders. Furthermore, it provides a practical, reproducible, and easily adoptable method for maintaining mouse colonies under physiologically relevant conditions, ensuring accessibility for a broad range of research institutions. Finally, our findings suggest that the impact of environmental factors on immunity is shaped by local microbiota exposure, potentially explaining the variability and limited reproducibility of studies across institutions – an important consideration for scientific rigor.

## Materials and methods

### Mice

All mice were on a C57BL/6 background. We crossed Foxp3^hCD2^ (gift of Dr. S. Hori (The University of Tokyo, Japan)),^14^ IL10^Thy1.1^ (gift of Dr. C. T. Weaver (UAB))^15^ and Nur77^GFP^ (Jax#16617)^16^ reporter animals (referred to as B6^Tg^). TCRα^k/o^ (Jax#2216) and Rag1^k/o^ mice (Jax# 002216) were obtained from Jackson (Jax#2216). Mice of both sexes were used, and we indicated the effect of gender where applicable. The experimental design included power analysis (G Power v. 3.1.9.2 ^17^) for sample size calculation and adhering to ARRIVE 2.0 Essential 10 guidelines.^18^ We used only a minimal number of mice to reach significance. The sample size and animal age were indicated for each experiment. All studies presented in this manuscript address complex systems that cannot be replicated using *in vitro* techniques. We utilized artificial intelligence (AI) to address the need to reduce animal use. The mice were euthanized at the indicated timepoints or when they met the humane endpoint. All animal experiments were approved by IACUC at GSU (protocol number A23008).

### Mouse ENV facility

A cohort of B6^Tg^ and TCRα^k/o^ mice from our standard vivarium (CNV mice) was moved to a one-of-a-kind, IACUC-approved, temperature/humidity-controlled mouse vivarium dedicated to the housing of mice exposed to environmental factors (ENV mice) that is located on the official Georgia State University property in a forested area 12 miles from our primary vivarium on the downtown campus. We used two systems: in-room and out-door enclosure. In-room: using standard box cages (GM-500, Tecniplast), local dirt was used as bedding (mixed 1:1 with corncob bedding) and replaced every 7 d. Dirt was collected from ~50 cm below the surface, stored for 2 d to stabilize the humidity and temperature, and harvested at least 2 d after rainfall to avoid rain-derived compounds. All the mice in our studies received the same dirt and diet (Teklad TD.7013) and tap water (ad libitum), thus eliminating the unknown historical exposure information inherent to using wild mice. Given that the microbiota contained in the dirt was likely to be a major driver of effects on the mucosal immune system, we also generated a control group housed in autoclaved dirt (ENV/AUTO; 121°C, 15  psi, 60 min, twice to ensure proper heat penetration). The efficacy of autoclaving was checked using commercial tests (Mesa Lab: Pro Chem Bowie-Dick type test, Spore Strips, autoclave tape, aerobic and anaerobic cultures) that we use in our CNV Germ-Free (GF) facility. As an additional control, we housed CNV mice on commercial mouse bedding and food pellets in the ENV facility. Out-door: the hutch top was constructed of pressure-treated wood. The bin was 90 cm high, and the attached hutch was another 90 cm in height. The structure was partially embedded in the ground. The bottom half of the structure was underground and filled inside with soil. The top half of the structure was above ground. An access panel was placed on one side of the hutch top, and the top of the hutch could be removed for access. The mice were provided with nesting material, above-ground plastic shelters, and the ability to burrow under large rocks and cohabitate to simulate the same repertoire of activities and mechanisms that mice in the wild would use to thermoregulate. A chain-link fence with a top surrounded the primary enclosure, protecting the mice from predators and preventing escape. This chain-link kept out larger predators such as foxes, coyotes, birds of prey, etc. For smaller predators such as snakes, the primary enclosure was a durable structure that was completely wrapped (bottom, walls, top) with 30 mm galvanized steel mesh. In the event of severe inclement weather, a tarp was provided to redirect precipitation away from the enclosure.

### Antibodies and reagents

Monoclonal antibodies (mAbs) were obtained from Fisher (eBioscience, BD), BioLegend, and R&D Systems. The chemicals used were from Fisher and Sigma (see Table S2).

### Tissue preparations, FACS and CyTOF analysis, and cell sorting

Cell preparation was performed as previously reported.^19^ Briefly, the lymph nodes and spleens were mechanically disrupted and filtered through a 40 μm mesh. Splenic (spl) RBCs were lysed with ACK buffer. A total of 1 × 10^6^ cells were used for staining. FcR was blocked with 2.4G2 antibody, and the cells were subsequently labeled with titrated fluorochrome- or metal-tagged mAbs. Dead cells were excluded with DAPI or fixable viability dye (FACS) or cis-Pt (CyTOF). The intestine was first digested with collagenase D and DNaseI at 37°C before staining, as reported.^19^ For CyTOF, the published protocol^20^ was followed with no modifications. Because of the expected 80%–90% sample loss, 5 × 10^6^ cells were used for CyTOF staining. For FTY720 studies, the mice were injected i.p. with FTY720 (Sigma) at 1 mg/kg dissolved in sterile water. Mouse blood was collected at the indicated time points, processed as spl, and analyzed by FACS. For fecal IgA analysis, feces (25 mg/mouse) were resuspended in 1 ml PBS, centrifuged at 100xg for 1 min, filtered through 100 and 70 μm filters, and centrifuged at 100xg for 1 min. Two hundred  microliters of the liquid was filtered through a 40 μm mesh, centrifuged at 10,000xg for 1 min, and washed 3x with 1 ml PBS. Microbial pellets were resuspended in 50  μl PBS + 1% BSA + 20% normal rat serum, incubated for 10  min, and labeled with titrated anti-IgA antibody. After 15  min of incubation at 4°C, the cells were washed 3x with PBS, resuspended in PBS + 1% BSA, filtered through a 40 μm mesh, and Syto-BC (Invitrogen) was added at a ratio of 1:10,000 to label the DNA. After 5 min of incubation, the cells were washed 2x with PBS, resuspended in PBS + 1% BSA, filtered through a 40 μm mesh, and analyzed by FACS or sorted into IgA^-^ and IgA^+^ populations using a cell sorter. The cells were analyzed by CytoFlex cytometer (B5-R3-V5, 3 lasers, 13 detectors; Beckman) or Cytoflex LX (N3-V5-B3-Y5-R3-I2; 6 lasers, 21 detectors; Beckman). The cells were sorted with an SH800 sorter (4 lasers, 6 detectors, optical filter pattern 2; Sony) or a CytoFlex SRT sorter (V5-B2-Y5-R3, 4 lasers, 15 detectors; Beckman; owing to the low robustness, the SRT was used as a backup instrument, only for pre-enriched samples (e.g., total CD4^+^ or naïve CD4^+^ cells were pre-enriched using kits from Miltenyi or Stem Cell Technologies). The CyTOF samples were analyzed on a Helios instrument (Cellular Analysis and Cytometry Core at Georgia Institute of Technology (Atlanta, GA)) or CyTOF XT instrument (Longwood Medical Area CyTOF Core at Harvard’s Dana Faber Institute (Boston, MA)). Data analysis was performed as reported.^20^ The gating strategy was performed as reported.^21^ FACS data was analyzed on a workstation computer equipped with FlowJo v10, using the following plugins: FlowAI, FlowSOM, FlowClean, FlowMeans, ClusterExplorer, to generate t-SNE plots. Machine-learning analysis (t-SNE, FlowSOM) was done as reported.^22^ ^,^ ^23^

### Cell culture

Cells were cultured in RPMI media supplemented with 10% (v/v) FBS, 1% (v/v) HEPES, 1% (v/v) non-essential aminoacids, 0.5% (v/v) essential aminoacids, 1% (v/v) sodium pyruvate, 1% (v/v) Pen/Strep, 2 mM glutamine and 50 μM 2-ME in the tissue culture incubator (37 °C, 5% CO_2_).

### Single-cell RNAseq and data analysis

We used the 10x Genomics platform as we did in the past.^24^ Mesenteric lymph nodes (mLN) and colonic lamina propria (cLP) from five individual mice per a group, per organ were FACS-sorted into live CD45^+^ cells, equal number was pooled (to reduce the differences between mice and create uniformed sample), adjusted to 1 × 10^6^ cells/ml and 1 × 10^4^ cells from each sample were subjected to in-Gel Bead Emulsion using Chromium iX controller according to manufacturer protocol. Up to 50 ng of cDNA was used for single-cell 5′ gene expression library construction. The quality of the libraries was assessed with a Bioanalyzer (Agilent) using DNA chips. For single-cell libraries, sequencing was performed on the NovaSeq X Plus 10B flow cell (2 × 150 cycles format) by Illumina HTS at the University of Florida, ICBR. The raw base call files (bcl) were processed using the 10x Genomics Cloud Analysis platform (https://www.10xgenomics.com/products/cloud-analysis). Initial processing, including sample demultiplexing, alignment, filtering, barcode assignment, and unique molecular identifier (UMI) counting, was performed using Cell Ranger (10x Genomics) with the Cell Ranger Aggr pipeline, v7.1.0. The reads were aligned to the mouse reference genome (mm10, version 2.2) using the STAR aligner implemented within Cell Ranger. Valid cell barcodes and UMIs were identified based on the 10x Genomics whitelist and error-correction algorithms. For datasets generated across multiple samples or sequencing runs, Cell Ranger *aggr* was used to aggregate individual gene-barcode matrices. Aggregation was performed at the molecular level using the *molecule_info.h5* files produced by *cellranger count* without the re-alignment of sequencing reads. To correct for differences in sequencing depth between libraries, normalization was performed using the default depth-based normalization, in which molecules are subsampled such that all libraries achieve equivalent effective sequencing depth prior to generation of the combined expression matrix. Only datasets processed with the same reference genome and compatible chemistry were included in aggregation. Cell Ranger’s default cell-calling algorithm was used to identify high-confidence cells based on distinct UMI count distributions, and low-quality cells (e.g., cells with less than 200 genes expressed or that express > 2500 genes were removed, and cells that had > 0.05% of mitochondrial-associated genes among their expressed genes) were excluded during this initial quality control step. The resulting filtered feature-barcode matrix was used for downstream analysis. Dimensionality reduction was performed using principal component analysis, followed by graph-based clustering and visualization using t-distributed stochastic neighbor embedding (t-SNE), as implemented in the Cell Ranger analysis pipeline with default parameters. In some datasets, a small number of genes not detectably expressed in the relevant cell populations were identified as technical artifacts and manually removed prior to downstream analysis. The replication free quality/sensitivity checks were performed by downsampling stability in Loupe (randomly subsampled to 3 × 10^3^ cells per group and confirmed that the relative cluster composition and marker patterns remained stable to downsampling) and repeated random subsampling (metadata was run 20 random subsamples (3 × 10^3^ cells/group) in Loupe; recomputed percent per cluster each time, and plotted the distribution of percentages) confirming the same pattern consistently for the target populations. The cell clusters were annotated based on differential gene expression and canonical marker genes. For each cluster, significantly enriched genes were identified and compared against established cell-type marker references (Table 1). Candidate cell identities were assigned using known lineage-specific markers and cross-validated with publicly available curated databases, including PanglaoDB,^25^ a comprehensive compendium of mouse and human single-cell transcriptomic markers, and CellMarker (v2.0),^26^ a manually curated database of experimentally supported cell markers across tissues and cell types. Final annotations were determined by consistency across multiple markers rather than reliance on single genes, and clusters enriched for cell cycle-associated genes were annotated as cycling states rather than distinct cell types. Ambiguous or low confidence clusters were conservatively labeled to avoid overinterpretation. This approach follows established best practices for manual cell type annotation in single-cell RNA sequencing studies.

**Table 1.** Properties of cLP and mLN cells based on the mouse type (CNV, ENV, ENV/AUTO).

cLP	Mouse type	Key features	Proposed phenotype
CD4^+^CD44^-^Foxp3^-^ (Tn)	**CNV**	High expression of T cell identity and protein synthesis genes (*Tcf7, Batf, Zfp36*)	Homeostatic naïve CD4⁺ T cells
	**ENV**	Inflammatory signaling and immune regulation (*Ifngr1, Bach2, Hif1a*)	Microbially-modulated CD4⁺ T cells
	**ENV/AUTO**	Stress and effector-prone signature (*Hspa1a, Bcl2l11, Batf*)	Activated/stressed CD4⁺ T cells, possible early effector or autoimmune signature
CD4^+^CD44^+^Foxp3^-^ (Tact)	**CNV**	Expression of migration regulators (*Ccr7, S1pr1, Klf2*), survival and anti-apoptotic factors (*Bcl2, Tsc22d3*), and stress responses (*Hspa1a/b, Hspe1*). Mild activation with preserved tissue recirculation capacity.	Migratory, homeostatically activated CD4⁺ T cells
	**ENV**	Regulatory transcription factors (*Bach2, Ikzf1/2, Zbtb20, Nr4a2*), immune modulators (*Cd74, Prkca, Tox*), metabolic plasticity (*Adk, Klf4*), and trafficking genes.	Transcriptionally reprogrammed, immune-tuned CD4⁺ T cells under microbial exposure
	**ENV/AUTO**	Strong inflammatory and effector cytokines (*Ifng, Il17a, Il22*), stress/inflammatory mediators (*Atf3, S100a4/6, Furin, Mapkapk2*), and tissue-associated markers (*Cxcr6, Lamc1, Il1r2*).	Pathogen- or autoantigen-driven effector CD4⁺ T cells with inflammatory bias
CD4^+^Foxp3^+^ (Treg)	**CNV**	Express genes such as Foxp1, Nfatc1, and Ccr7, which suggest some regulatory capacity and migration potential. However, without high expression of IL-10, CD39, CD73, or CTLA-4, their suppressive activity is likely less robust.	Moderate to low suppressive cells; they lack the key markers of potent suppression seen in other groups.
	**ENV**	The high expression of IL-10, CD39, CD73, and CTLA-4 strongly indicates highly suppressive Tregs.	Well-equipped suppressor cells are prone to inhibit inflammatory responses, particularly in tissues prone to immune activation
	**ENV/AUTO**	Express markers such as CTLA-2A, Tnfrsf18, and Gpx4, which suggest enhanced survival and suppression under inflammatory conditions.	Tregs with a generalized regulatory phenotype, possibly focused on Th2-type responses and survival under oxidative stress
CD8^+^CD44^-^ (CD8 naive)	**CNV**	Primed for immune surveillance and activation, with markers related to T cell migration (e.g., *Ccr7*, *S1pr1*) and potential for rapid response to signals. These cells are likely more quiescent but responsive when needed.	Likely homeostatic, maintaining immune surveillance with quick activation potential.
	**ENV**	Show a pro-inflammatory and cytotoxic phenotype, with markers indicating active effector functions and cytotoxicity (*Gzma*, *Gzmb*). They are highly involved in inflammatory responses and may have a memory-like function, acting quickly to clear infections or control inflammation.	Cytotoxic and effector-driven, designed for responding to acute infections or inflammation.
	**ENV/AUTO**	Involved in regulation and inflammation with high expression of stress response genes (*Hspd1, Hsph1, Gadd45b*, and *Fosb*) and cytokines (*Ccl2, Il1b*, and *Tnfsf18*). These cells might play a role in chronic inflammation, tissue repair, or immune regulation rather than just acute immune responses.	Involved in long-term inflammation or regulation, with a broader role in tissue remodeling and sustained immune responses.
CD8^+^CD44^-^ (CD8 act)	**CNV**	*Cish*, *Tnfrsf9* (TNFR family member, suggests involvement in costimulation and activation), *Socs1* (negative feedback on cytokine signaling), *Traf4*, *Icos* (involved in T cell activation and survival). *Tnfsf11* (involved in immune modulation), *Rflnb*, *Bst2* (suggests possible role in immune response modulation or signaling).	Effector memory CD8 + T cells, with a focus on T cell activation, homing, survival, and modulation of immune responses through co-stimulatory and inhibitory signals.
	**ENV**	*Tigit*, *Ccl5* (indicates immune response regulation and chemotaxis), *Gzma* (cytotoxic effector molecule), *Cst7* (suggests involvement in immune modulation). *Rgs1*, *Litaf* (associated with inflammatory responses), *Jun* (transcription factor indicating stress response), *Fosb* (involved in the activation of immune cells).	Cytotoxic effector phenotype with a strong capability for immune modulation, likely effector CD8 + T cells activated in the context of inflammation or infection.
	**ENV/AUTO**	*Tnfsf11*, *Ccl4*, *Ifng* (cytokines suggesting a pro-inflammatory response), *Cebpb* (involved in inflammation and immune cell activation). *Nfil3*, *Ywhae* (involved in transcriptional regulation of immune responses).	Highly activated cytotoxic CD8 + T cells, possibly associated with effector functions in chronic inflammation.
CD19^+^ (B cells)	**CNV**	Enrichment in heat shock proteins (Hspa1b, Hsp90aa1, Hspd1) suggests cellular stress responses. Plk2, Junb, Pim1, Rasgef1b suggest proliferative and activated B cells. Nfkbia, Nfkbid, Ifi30, Fcer2a, and Cd79b point to antigen presentation and B cell receptor (BCR) signaling. Ig-related genes (Igkv1-135, Iglc2) support active immunoglobulin production.	Activated, proliferative, stress-responsive B cells
	**ENV**	Peak1, Zhx2, Fgfr2 suggest metabolic and structural adaptations. Cd86, Cybb, Dock10, Adgre5 suggest B cells with antigen-presenting potential but reduced activation. Crip1, Vim, Rhob indicate a tolerogenic, possibly regulatory phenotype. Ig-related genes (Igkv10-96) indicate some BCR signaling, but not as prominently as CNV.	Tolerogenic B cells with metabolic adaptation
	**ENV/AUTO**	Cd79b, Btg2, Ighm indicate B cell receptor (BCR) activation and memory B cell presence. Nfkb1, Stat4, Kdm6b suggest active transcriptional regulation and inflammation. Egr1, Gadd45b suggest a response to stimuli and the potential for differentiation. Hsp90b1 and Calr indicate antigen presentation and endoplasmic reticulum stress response.	Memory-like B cells with enhanced antigen presentation
CD11c^+^CD11b^-^ (DC)	**CNV**	Macrophages or monocyte-derived cells: *C1qa, C1qb, C1qc (complement components), Csf1r (macrophage marker), Aif1 (macrophage marker), Lyz2 (lysozyme)*, *Msrb1 (reactive oxygen species management)*, *Apoe (lipid metabolism)*, *Clec4n (C-type lectin receptor)*, and *Fermt3 (involved in cell adhesion)*.	Macrophage-like with phagocytic and tissue-repairing roles, reflecting an immune surveillance or homeostasis function
	**ENV**	Dendritic cell or activated macrophage phenotype: *Ccr7 (migratory marker for dendritic cells)*, *Il1r2 (inflammation-related receptor)*, *Ifitm1 (involved in immune response)*, *Il1b (pro-inflammatory cytokine)*, *Foxp1 (transcription factor regulating immune cell function)*, *Syk (tyrosine kinase involved in signal transduction in immune cells)*, *Bst2 (immune response)*, and *Cd274 (PD-L1, immune checkpoint molecule)*.	Dendritic cells with strong antigen presentation, T-cell activation, and inflammatory response capabilities, suggesting they play a role in immune activation.
	**ENV/AUTO**	Inflammatory or cytotoxic dendritic cells, with some overlap with macrophage-like features: *Csf2 (granulocyte-macrophage colony-stimulating factor)*, *Gzma (granzyme A, cytotoxic marker)*, *Tnfrsf18 (TNF receptor involved in immune cell regulation)*, *Rora (transcription factor involved in inflammation and immune response)*, *Map3k5 (involved in cell signaling and inflammation)*, *Gzmc (granzyme C)*, *Mpeg1 (macrophage-expressed gene involved in pathogen defense)*, and *Tnfrsf9 (TNF receptor, immune regulation)*.	Cytotoxic dendritic cell phenotype with heightened inflammation and potential for cytotoxic activity, positioning them as key players in immune modulation and pathogen defense.
CD11c^+^CD11b^+^	**CNV**	Tnfsf14 (TNF superfamily, inflammation), Egr1 (immune response), Smad7 (TGF-*β* signaling inhibitor), Klrb1b (NK cell marker), Ccl4 (chemokine signaling), Cd2 (T-cell activation)	Inflammatory and immune regulatory with cytotoxic properties
	**ENV**	Il22 (epithelial repair), Il10 (immune suppression), Icosl (T cell costimulation), Nlrp3 (inflammasome, immune modulation), Csf1r (monocyte/macrophage regulation), Thbs1 (tissue remodeling), Retnla (anti-inflammatory macrophage marker)	Anti-inflammatory, regulatory, and tissue-repair functions
	**ENV/AUTO**	Klra4/Klra9/Klrb1a (NK receptor family, cytotoxic function), Tnfrsf18 (GITR, Treg and immune activation), Myb (dendritic cell differentiation), Pdgfa (fibrosis, tissue remodeling), Eomes (T-cell cytotoxicity and differentiation), Tcf7 (T cell activation)	Cytotoxic and highly activated antigen-presenting phenotype
CD11c^-^CD11b^+^	**CNV**	Immune regulation & trafficking: *Ccr7*, *Cd2*, *Cd69*, *Ptprcap.* Heat shock/stress response: *Hspa1a/b*, *Ddit4.* Antigen presentation & immune modulation: *H2-DMb2*, *Cd72*, *C1qa/b*, *Gimap4.*Transcriptional control: *Ebf1*, *Mef2c*, *Smad7*. Cell metabolism & survival: *Phgdh*, *Eif2ak3*, *Calcrl*, *Pml.* T cell activation & cycle: *Ccnd2*, *Apobec3*	Macrophage-like antigen-presenting cells and B-cell-associated immune regulators, with a balance of inflammation control and adaptive immune communication.
	**ENV**	Regulatory transcription factors: *Tox, Ets2, Ikzf3, Trps1, Setbp1*. Cytoskeletal & signaling adapters: *Zyx, Cnn2, Ypel3, Gngt2, and Fis1*. Immune modulation: *Serpinb9b, Nfam1, Cast, GpX1*. Metabolic & oxidative stress response: *Msrb1, Crip1, Osbpl9*. Transcription and RNA processing: *Hist1h1e, Polr2a, Qk*	Higher cytotoxicity potential, antioxidant defense, and stress-response adaptation, suggesting they might be more specialized for immune tolerance and tissue repair.
	**ENV/AUTO**	Strong pro-inflammatory signature: *Il1b*, *Ccl2*, *Cxcl10*, *Xcl1*, *Slpi.* Type I/II interferon responses: *Isg15*, *Ifi205*, *Ifitm1*, *Ifngr2.* Tissue remodeling & repair: *Thbs1*, *Tgm2*, *Dgat1*, *Plec*. Immune activation & suppression: *Cd83*, *Socs3*, *Crem*, *Lilr4b*, *Il1r2.* Metabolism & stress adaptation: *Acod1*, *Fabp5*, *Uck2*, *Dusp2/3*	Strong inflammatory signature, with high expression of chemokines, cytokines, and tissue remodeling genes, suggesting high activation and antimicrobial defense, possibly at the expense of immune regulation.
**mLN**	**Mouse Type**	**Key Features**	**Proposed Phenotype**
CD4^+^CD44^-^Foxp3^-^ (Tn)	**CNV**	High translation (Rps21, Rps28, Rps29, Rpl35a, Rpl41, Rpl37, Rpl37a, Rpl39), metabolic activity (Atp5e, Atp5mpl, Cox7c, Cox6c, Ndufa2), MHC class II presentation Cd74, H2-Aa).	Highly metabolic naive CD4 cells with strong activation potential
	**ENV**	Stress-resistance (Hsp90ab1, Hspe1, Hspa8, Dnaja1, Ucp2), survival genes (Bcl2, Txnip, Ddit4, Foxn3), adaptive proliferation (Ccnd3, Rsrp1, Pdcd4)	Stress-adapted naive CD4 cells with survival advantage
	**ENV/AUTO**	Strong T cell interactions (Tcf7, Rasgrp1, Rasa3, Grap2, Dock11), cytoskeletal changes (Actn1, Msn, Flna, Apbb1ip, Evl), memory potential (Tspan32, Dgka, Arhgef18)	Memory-like naive CD4 cells with regulatory properties
CD4^+^CD44^+^Foxp3^-^ (Tact)	**CNV**	High mitochondrial activity, ribosomal genes, Ccr7, Foxo1	Memory T cells, homeostatic maintenance
	**ENV**	Il7r, Stat1, inflammatory and metabolic genes	Activated T cells, inflammatory response
	**ENV/AUTO**	Top2a, Mki67, chromatin regulators, histone genes	Highly proliferative, effector T cells
CD4^+^Foxp3^+^ (Treg)	**CNV**	Highly expressed genes involved in chromatin remodeling (Hmgb2, H2afv), metabolism (Ndufa3, Cox7b, Atp5mpl), ribosomal activity (Rps28, Rpl35, Rpl37), and T cell signaling (Pik3cd, Prkcq, Grap2).	Highly proliferative and metabolically active Tregs with enhanced survival signaling.
	**ENV**	Enriched in immune response regulators (Stat1, Nfkbia, Pdcd4), inflammatory mediators (Pycard, Txnip, Ifi203), and stress response genes (Hsp90aa1, Junb).	Inflammatory, highly suppressive and stress-responsive Tregs, adapted to environmental challenges.
	**ENV/AUTO**	Express markers such as CTLA-2A, Tnfrsf18, and Gpx4, which suggest enhanced survival and suppression under inflammatory conditions.	Tregs with a generalized regulatory phenotype, possibly focused on Th2-type responses and survival under oxidative stress
CD8^+^CD44^-^ (CD8 naive)	**CNV**	Increased expression of components of the translation machinery and cellular respiration (Rps29, Rps28, Rpl35a, Atp5e). Immune signaling and survival (Jund, Rbm3, Crip1).	Cytotoxic cells with enhanced protein translation, immune signaling, and survival
	**ENV**	Activation or potential transition to an effector or memory state, with the expression of genes involved in cell migration, signaling, and survival (Il7r, Ccl5, Hspa8, and Bcl2). Pde3b, Ccl5, and Tmtc2 suggest chemotactic and immune response pathways	Highly cytotoxic/effector cells with enhanced migratory potential
	**ENV/AUTO**	Differentiation or activation towards an effector phenotype (Sp100, Maml2, Stat1, Ctsw), immune cell signaling (Cd37, Ifi203), and cellular adhesion (Flna, Itgb2). Actn1, Myh9, and Nlrc5 suggest cytoskeletal remodeling and immune signaling. Strong signaling through immune receptors (Stat1, Pde3b, Filip1l).	Effector T cells expressing factors involved in immune response regulation and stress response
CD8^+^CD44^-^ (CD8 act)	**CNV**	Metabolic and stress-response genes (Hspa5, Hspd1, Ldha, Phb2), cell cycle regulators (Id3, Cdk17)	Homeostatic or metabolically active memory CD8 + T cells
	**ENV**	Immune activation and inflammatory genes (Ccl5, Il2rb, Ifngr1, Stat4, Hopx)	Activated cytotoxic and inflammatory effector CD8 + T cells
	**ENV/AUTO**	Tissue-resident markers and immune regulators (Foxo1, Ctsw, Ms4a4c, Klrd1, Trbv19)	Tissue-resident memory-like CD8 + T cells with enhanced immune surveillance
CD19^+^ (B cells)	**CNV**	Genes like Hmgb1, Rps28, Rpl36a point to active protein synthesis and cellular differentiation. Rpl41, Vpreb3, and Snrpg indicate B cell maturation and the immune response. Iglc3, Iglc2 suggest immunoglobulin production. Cox6b1, Apoe, Crip1 imply a stress response and immune modulation. Tma7, Sec61g suggest functional adaptations in the endoplasmic reticulum and protein folding.	Proliferative, differentiated B cells with stress and immune activation
	**ENV**	Fgfr2, Klf2, Irf8 suggest immune regulation and signaling pathways. Hspa5, Hspa8, Hsp90aa1 point to the stress response. Ighm, Syk, Igkv1-135 suggest immunoglobulin production and signaling. Dock10, Serinc3 imply immune cell migration and lipid metabolism. Jchain, Igkc, Ighg1 indicate class switching and immunoglobulin secretion.	Adapted B cells with metabolic regulation and immune tolerance
	**ENV/AUTO**	Ifi27l2a, Fcer2a, Fcmr point to immune activation and antigen presentation. Ighv2-9-1, Ighv6-3 indicate immunoglobulin variable regions, suggesting BCR diversification. Lcp1, Ezr, Fchsd2 point to immune cell motility and signal transduction. Fgfr2, Zc3h7a suggest cellular survival and differentiation regulation. Igkv10-96, Igkv12-46 suggest BCR diversity and antibody secretion	Memory-like B cells with antigen presentation and inflammatory response
CD11c^+^CD11b^-^ (DC)	**CNV**	Activation and stress response features, with increased expression of immune-related proteins and ribosomal components, suggesting an active immune state Hmgb2, Cd7, Tmsb10, Rps21, S100a11, Arhgdia).	Activation, Protein Synthesis, Stress Response
	**ENV**	focus on immune signaling and cell cycle regulation, marked by the expression of chemokines, heat shock proteins, and ribosomal proteins, indicating a heightened immune response (Ccl5, Cdk8, Eif5a, S100a6, Hspa5)	Immune Signaling, Cell Cycle, Stress Response
	**ENV/AUTO**	inflammatory and immune-regulatory phenotype with elevated levels of immune activation markers, stress response genes, and proteins involved in cell survival and immune response (Hmgb2, Ccr7, Ctsb, S100a4, Mpeg1)	Immune Activation, Inflammation, Stress Response
CD11c^+^CD11b^+^	**CNV**	Fcna, C1qa, C1qb, C1qc: Complement system regulation, inflammation. Csf1r: Macrophage colony-stimulating factor receptor, macrophage differentiation. Cx3cr1: Microglial and monocyte migration and activation. Apoe: Lipid metabolism, immune regulation. Selenop, Grn, Fcgr3: Involved in antioxidant defense and innate immunity.	Macrophage-like, inflammation, antigen presentation
	**ENV**	Dscam: Immune cell adhesion and signaling. Stat2, Cd7: Cytokine signaling and immune response regulation. Ctnnd2, Vasp: Cell adhesion, migration, and cytoskeletal organization. Alcam, Ccnd1: Cellular adhesion and proliferation regulation. Mapk14: Stress response and inflammatory pathway activation. S100a9: Inflammatory response, immune cell recruitment.	Dendritic-like, cytokine signaling, adhesion
	**ENV/AUTO**	Hist1h2ae, Tubb5, Tuba1b: Cytoskeletal components, cell division. Xcl1: Chemokine involved in immune cell migration. Irf7, Nfat5: Transcription factors involved in immune response. Naaa, Ppt1: Involved in lipid metabolism and antigen processing. Cdc5l, Mki67: Cell cycle regulation, proliferation markers.	Activated dendritic cells, immune response, cell division
CD11c^-^CD11b^+^	**CNV**	Involved in immune regulation and inflammation, with markers of antigen processing and presentation (e.g., Hck, Csf1r, and Cebpb). Features suggest a cellular response to immune activation (e.g., Nr4a1, Gngt2, Ifi27l2a) and cell survival.	Likely activated antigen-presenting cells (APCs) or macrophage-like cells
	**ENV**	Key involvement in antigen presentation (H2-Ab1, H2-Eb1), immune regulation, and inflammation (e.g., Ccl6, Csf3r, S100a11). The cluster suggests a role in adaptive immunity and inflammation.	Primarily antigen-presenting cells (APCs), likely dendritic cells or macrophages.
	**ENV/AUTO**	Involved in immune responses and antigen presentation (H2-Ab1, C1qb), with markers of inflammation (S100a9, S100a8, Ccl5). Features indicate response to infection and tissue remodeling.	Likely dendritic cells or activated macrophages.

Gene Ontology (GO) biological process analysis and networks were generated with ShinyGO 0.82 (https://bioinformatics.sdstate.edu/go/) against the Mus musculus genome (Ensembl: mmusculus_gene_ensembl, assembly name:Mouse genes GRCm39, taxonomy ID:10090, source:ENSEMBL) with a cutoff of 25 pathways^27^ after pre-processing using DAVID (Database for Annotation, Visualization, and Integrated Discovery; https://davidbioinformatics.nih.gov/home.jsp#).^28^ Pathway and biological process enrichment analysis for each gene list was performed using the Metascape web‑based platform (https://metascape.org/gp/index.html#/main/step1), following the analysis workflow and summary description provided by the website.^29^ Metascape integrates multiple established knowledge bases, including Gene Ontology biological processes, KEGG pathways, Reactome gene sets, WikiPathways, and uses all genes in the genome as the background reference set. React Enriched terms were selected based on statistical significance, representation by at least three genes, and enrichment above random expectation. Metascape automatically groups related enriched terms into clusters based on shared gene membership to highlight overarching biological themes. For each cluster, the most statistically significant term is reported as the representative annotation. Unprocessed sequencing data were deposited in NCBI (accession number: PRJNA1373468).

### Metabolic studies

The metabolic capabilities were assessed in mLN, using flow cytometry (Met-Flow analysis;^30^ 8 different metabolic targets: Glucose transporter member 1 (GLUT1), Glucose-6-phosphate dehydrogenase (G6PD), Hexokinase 1 (HK1), Carnitine palmitoyltransferase 1A (CPT1A), Acetyl-CoA carboxylase alpha (ACC1), Isocitrate dehydrogenase 2 (IDH2), ATP synthase F1 subunit alpha (ATP5A), Argininosuccinate synthase 1 (ASS1), and Peroxiredoxin 2 (PRP)) with titrated mAbs and unstained controls.

### Gut microbiota composition analysis

Microbiome diversity among bacteria, fungi, viruses, protists, and archaea was assessed using Transnetyx’s Microbiome service (https://www.transnetyx.com/microbiome). Briefly, freshly collected fecal pellets or dirt were stored in Transnetyx’s buffer and submitted to the company for the downstream procedures (nucleic acid isolation, library construction, and sequencing). The data were analyzed by Transnetyx using OneCodex software. PCoA and taxonomic plots were used as descriptive visualizations of overall microbiota structure. Accordingly, conclusions regarding immune remodeling are based on comparative exposure conditions and functional immune readouts rather than species-level abundance differences inferred from ordination plots alone. PCoA was performed using Bray-Curtis dissimilarity on species-level relative abundance profiles in One Codex, and alpha diversity was assessed using the Shannon diversity index. 16S rRNA gene amplification was performed according to the Illumina 16S rRNA Metagenomic Sequencing Library preparation guide, as previously described ^19^, and the genes were classified taxonomically using Silva reference database (138.2, 2025-09-04). 16S rRNA sequencing was done at the Genomics Facility at Cornell University. Unprocessed sequencing data were deposited in NCBI (accession number: PRJNA1373468).

### Microbiota depletion

Single agents or combinations were used to deplete Gram-negative or Gram-positive bacteria or fungi. Three days before the transfer to the ENV facility, the mice received antimicrobials (0.5x final concentration to gradually habituate the animals to antibiotics (Abx)) in drinking water, and the treatment continued with the full Abx dose until the mice were analyzed. Ceftazidime (0.5 mg/ml (w/v)) was used to deplete Gram-negative bacteria. Vancomycin (0.5 mg/ml (w/v)) depleted Gram-positive bacteria. Fluconazole (0.5 mg/ml) + amphotericin B (0.1 mg/ml) + 5-fluorocytosine (5 mg/ml) (all w/v) targeted fungi. Control mice received unmedicated water. To counteract the medication taste, the water contained 5 g/L (w/v) sucrose. Microbiota depletion was confirmed by qPCR^19^ and metagenomic analysis.

### Quantitative real-time PCR (qPCR)

One microgram of each DNA-free RNA was isolated with Qiagen RNeasy Mini kit with on-column DNA digestion and reverse transcribed using SuperScript IV kit (Invitrogen) and random hexamers. qPCR was performed in triplicate using Sybr Green on a StepOnePlus PCR system (Thermo). Data were normalized to Gapdh, 16S rRNA, or ITS1. The primers used were from IDT, and their sequences are listed in Table S2.

### Histology

The tissues were fixed with 10% formalin for at least 24 h and submitted to HistoWiz (Brooklyn, NY) for processing and H&E staining. Colitis scoring (0, no discernible inflammation; 1, small, focal focus of inflammation; 2, small, multiple foci of inflammation; 3, multiple large foci of inflammation; and 4, significant inflammation with parenchymal destruction) was performed in a blind manner.

### Chronic colitis models

FACS-purified naïve CD4^+^Foxp3^hCD2-^CD44^-^CD45RB^high^ cells (Tn; 1 × 10^6^) were adoptively transferred by iv injection into TCRα^k/o^ mice. The animals were monitored for signs of colitis (weight loss, stool consistency) and euthanized for analysis (FACS, histology) at the indicated time points.

### LPS sepsis model

The mice were i.p. injected with ultrapure LPS (*E. coli* O55:B5; InvivoGen) at 10 mg/ml. The mice were observed for 2 h post-injection, three times a day for the first 72 h, and then two times a day for the remaining duration of the experiment.

### STZ diabetes model and glucose tolerance test (GTT)

The mice were ip injected with streptozotocin (STZ; Sigma) dissolved in citrate buffer (pH 4.5) at 20 mg/kg/day for 5 d. Blood glucose was measured at the indicated timepoints. For the GTT, the mice were fasted overnight and ip injected with glucose (1.5 g/kg; Sigma) dissolved in PBS. Blood glucose levels were measured at the indicated timepoints.

### Experimental schematics and a graphical abstract

All experimental schematics and graphical abstracts were created using BioRender (https://biorender.com/).

### Statistical analysis

Direct comparison between two groups was calculated with an unpaired Student’s t-test. When more than two groups were compared, one-way ANOVA followed by Tukey’s post-test for multiple comparisons was used. Kaplan–Meier survival analysis was used where needed. Statistical analysis was performed with GraphPad Prism v10 software. *p < 0.05, **p < 0.01, ***p < 0.001, ****p < 0.0001.

### The use of artificial intelligence (AI)

The manuscript was entirely written by MK and edited by all co-authors with no use of generative AI. We used Grammarly (grammarly.com) to revise the grammar and punctuation. Then, we used Microsoft Copilot 365 to read the text and suggest the changes to improve clarity. The following prompt was used: “I have written a scientific paper in the field of mucosal immunology. Please act as an expert in this field and help me edit the text for grammar, clarity, and flow. I will paste fragments of the paper for you to review. Keep all figure placements (e.g., Figure X, Figure SX) exactly where they are. Do not alter scientific meaning or terminology unless something is clearly incorrect or unclear. If you believe something important is missing or could strengthen the argument, add a suggestion in a separate paragraph, clearly marked as such. Maintain a formal scientific tone appropriate for a peer-reviewed journal in immunology. Make sure to use language resonating with the immunology researcher but also understandable by layman. Make an output document in .docx format”. The proposed improvements were implemented if found necessary. AI was also used to cluster scRNAseq upregulated genes into categories, which were then manually confirmed to be relevant using PubMed. The following prompt was used in Copilot: “Analyze the following gene clusters using the structured format: Identify cell types and phenotypes, create a gene-function table for each cluster, and summarize all clusters in a final table. These are genes from [ORGAN NAME, POPULATION NAME].”

## Results

### Establishment of a novel housing model for environmental exposure of mice (ENV)

**Figure 1.**
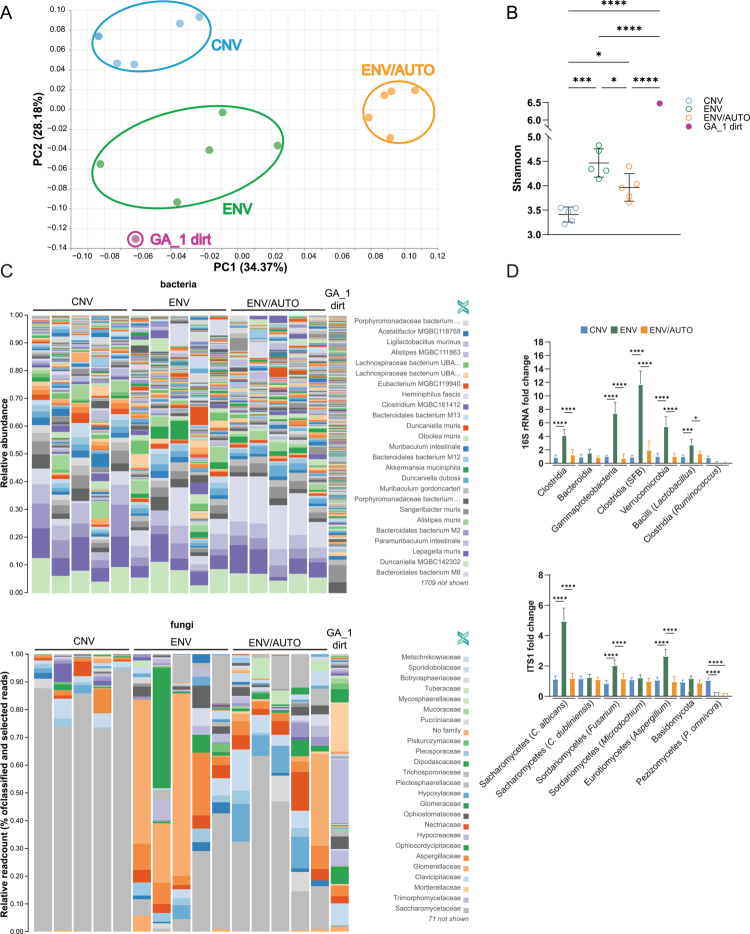
Colonization of mice with the dirt microbiome. (A) PCoA of fecal metagenomic data from CNV, ENV, and ENV/AUTO B6^Tg^ mice housed for 8 weeks in the respective conditions (*n* = 5 randomly chosen samples from 5 separate cages (one mouse per cage; each stock cage originated from a different breeding pair (i.e., the analyzed mice were not littermates)) is shown). GA_1 indicates the dirt sample collected from the ENV facility. Bray‒Curtis dissimilarity calculated from the species‑level relative abundance profile. (B) Alpha diversity measured by the Shannon index (followed by one-way ANOVA) of the samples presented in (A). (C) Typical metagenomic analysis of the fecal microbiota (bacteria (top) and fungi (bottom)) of the indicated mice. Data was analyzed with OneCodex software (Transnetyx). Data from five mice of *n* = 8 per group are shown. (D) qPCR analysis of fecal bacteria (top) and fungi (bottom). Bars indicate the relative expression calculated from six mice from six separate cages of each group. *p < 0.05, ****p < 0.0001 assessed with one-way ANOVA followed by Tukey’s post-test.

To address the paucity of mouse models exposed to environmental factors, we developed a scalable, cost-effective system where local dirt used as bedding in standard isolator cages is a source of ambient microbes, nutrients, and metabolites. The mice were housed in two settings: (1) a dedicated indoor vivarium and (2) a fenced outdoor enclosure (Figure S1A, C), located on Georgia State University property in a forested area ~12 miles from our downtown campus. We housed B6 mice expressing Nur77^GFP^, Foxp3^hCD2^, and IL-10^Thy1.1^ reporters (B6^Tg^; Figure S1B). The ENV mice remained viable for over 12 months, comparable to the age- and sex-matched conventional (CNV) mice housed under SPF conditions on corncob bedding in our main vivarium (downtown campus). Importantly, the lymphopenic ENV mice (TCRα^k/o^) also remained healthy, with no signs of morbidity. Pathogen screening (IDEXX Bioanalytics) and metagenomic sequencing (Transnetyx) confirmed the absence of life-threatening (e.g., Hantavirus^31^) or immune-modulating (e.g., Helicobacter, pinworm) pathogens in feces (Figure 1C, D) and dirt (Figure S1E).

GSU B6 mice purchased from Jax Labs were housed in cages, and those in the outdoor enclosure showed similar immune phenotypes, indicating that dirt microbes drive the ENV phenotype (Figure S1F), suggesting broad adaptability of the ENV system. However, differences in the local dirt microbiota – discussed later – should be considered when implementing this model elsewhere. While both indoor and outdoor ENV systems are maintained for future studies on environmental stressors (e.g., predator sounds, burrowing, and airborne pathogens), and to ensure similar housing conditions (cages) with CNV mice, all data presented here were generated using the indoor system. All CNV data presented here were derived from mice housed in our main SPF vivarium.

### Dirt microbiota colonizes environmental mice

**Figure 2.**
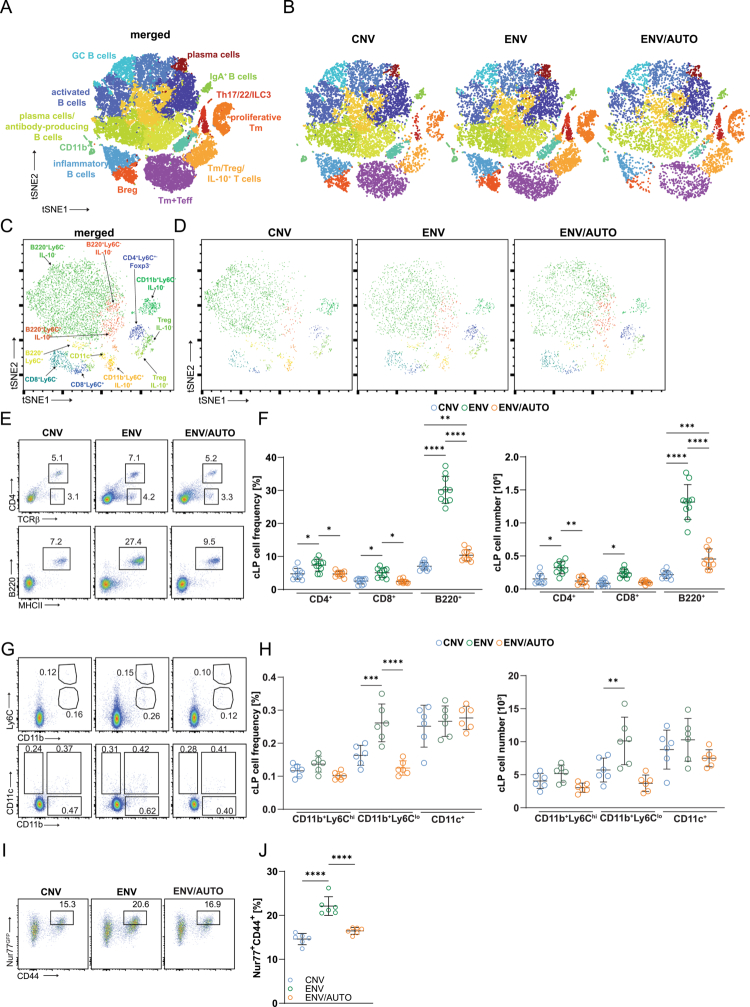
Housing of mice in dirt influences the intestinal innate and adaptive immune system. (A, B) t-distributed stochastic neighbor embedding (t-SNE) data of FACS-sorted CD45^+^ cells from cLP. The colors indicate clusters (*n* = 18) identified by cLoupe software (10x Genomics) based on the gene expression. Several populations impacted in ENV mice are indicated in the merged t-SNE plot (A). Each dot represents a single cell (*n* = 1 × 10^4^/sample; 3 pooled mice/group). (C, D) CyTOF analysis of cLP of the indicated mice. Populations affected by the environmental microbiota are indicated on the merged t-SNE plot (C). (E–G) FACS analysis of cLP adaptive (E, F) and innate (G, H) immune cells. (I, J) Environmental model mice have an increased population of activated T cells. Representative FACS plots of live CD4^+^ cells expressing CD44 and Nur77^GFP^. The cell frequencies and/or numbers are summarized on the graphs (F, H, J) with each symbol indicating an individual mouse. For (A–D), representative data of one of 5 mice is shown. *p < 0.05, **p < 0.01, ***p < 0.001, ****p < 0.0001 one-way ANOVA followed by Tukey’s post-test.

**Figure 3.**
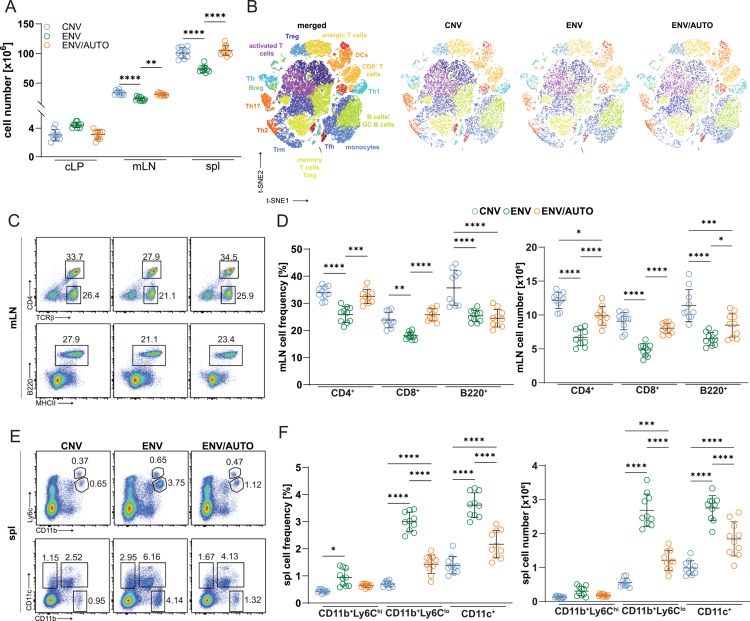
Housing of mice in dirt influences the peripheral innate and adaptive immune system. (A) Numbers of cLP, mLN, and spl cells are shown in CNV, ENV, and ENV/AUTO mice (*n* = 10 mice/group). (B) t-SNE data of FACS-sorted CD45^+^ cells from mLN (*n* = 1 × 10^4^/sample; 5 pooled mice/group). Populations affected by the environmental microbiota are indicated in the merged t-SNE plot (B). Each dot represents a single cell (*n* = 1 × 10^4^/sample). (C, E) FACS analysis of (C) mLN adaptive and (E) spl innate immune cells. The cell frequencies and numbers are summarized on graphs (D, F) with each symbol indicating an individual mouse. *p < 0.05, **p < 0.01, ***p < 0.001, ****p < 0.0001 one-way ANOVA followed by Tukey’s post-hoc test.

To assess how dirt exposure alters the gut microbiota in ENV mice, we performed metagenomic analysis of the dirt we exposed our mice to and compared it with dirt samples from several other geo locations within the US (Figure S1H). This analysis showed uniformity of Georgia (GA) samples and higher similarity of South-East (GA, SC, and AL) dirt microbiota compared to other states. We then performed metagenomic (Figure 1A–C) and amplicon sequence variant (ASV) (targeting 16S rRNA (bacteria) along with qPCR of fecal samples (Figure S1D, E) from 16-week-old mice after 8 weeks of exposure – when immune phenotypes stabilized (Figure S1G). To isolate the effects of live microbes, we included a control group housed on autoclaved dirt (ENV/AUTO) (Figure 1; Figure S1A). Microbiota profiling revealed significant shifts in microbial communities in ENV mice (Figure 1, Figure S1D, E). In contrast, ENV/AUTO mice showed modest but significant compositional shifts at the species/genus level, which is consistent with exposure to heat-killed microbes preventing active colonization but is able to influence immunity through PAMPs, antigens, etc. (Figure 1C, D, Figure S1D, E). However, they clustered separately from CNV mice, as shown in the PCoA plot (Figure 1A, Figure S1D), and showed higher richness than CNV microbes (Figure 1B), suggesting that microbial antigens alone can partially modulate immunity,^19^ ^,^ ^32^ which is supported by modest immune changes in ENV/AUTO mice (Figures 2 and 3).

In ENV mice, expanded bacterial taxa included *Verrucomicrobia*, *Clostridia* (notably segmented filamentous bacteria, SFB), *Bacilli* (*Lactobacillus* spp.), *Gammaproteobacteria*, and *Bacteroidia*. Fungal taxa included *Saccharomycetes* (*Candida albicans*, not *C. dubliniensis*), *Sordariomycetes* (*Fusarium* spp., not *Microdochium*), and *Eurotiomycetes* (*Aspergillus* spp.). The reduced taxa included *Ruminococcus* (*Clostridia*), *Basidiomycota*, and *Pezizomycetes* (Figure 1C, D, Figure S1E). Importantly, only the Georgia dirt samples showed a higher abundance of *Verrucomicrobia*. As reported elsewhere,^9^ ^,^ ^33^ colonization with dirt microbiota did not require prior antibiotic-mediated depletion, indicating the competitive advantage of wild microbes in colonizing established gut niches. We initially avoided antibiotics because of reports of morbidity in germ-free mice colonized with wild microbiota,^4^ aiming to better reflect natural human exposure. Later, experiments using antibiotics (Figures S9 and S11) showed no mortality, and the microbial profiles remained consistent after 8 weeks, regardless of antibiotic use, reinforcing the dominance of environmental microbes.

In summary, dirt exposure induced robust changes in the gut microbiota. Wild microbes successfully colonized the gut, outcompeting resident communities and establishing an immunologically active microbiome. In contrast, ENV/AUTO mice retained CNV-like microbiota. Though non-viable microbes delivered PAMPs (e.g., LPS, *β*-glucans) and antigens that partially primed immunity via PRRs (e.g., TLRs, NLRs), the absence of live microbial interactions limited metabolite production (e.g., SCFAs^34^ ^,^ ^35^) and impact on gut homeostasis.

### Dirt exposure remodels intestinal immunity

In addition to reshaping the gut microbiota (Figure 1), housing adult mice on dirt profoundly altered intestinal immunity. After 8 weeks of ENV housing, both innate and adaptive immune compartments were affected (Figure 2). Single-cell RNA sequencing (scRNAseq) (Figure S2E) revealed the expansion of colonic lamina propria (cLP) innate populations, including *Itgam⁺* (CD11b⁺), *Itgax⁺* (CD11c⁺), and *Rorc⁺* ILC3 cells (Figure 2A, B), which are likely responding to novel microbial antigens. Adaptive responses included increased CD44⁺ and Nur77^GFP⁺^ T cells (Figure 2C, D, I, J), indicating antigen-specific activation. Colonic B cells (CD19⁺), especially IgA⁺ subsets, were also expanded (Figure 2A, B, E, F), which is consistent with their role in microbiota regulation.^36^ Flow cytometry confirmed elevated numbers of activated B cells expressing IgA in cLP (Figure 2A, B; Table 1) and mLN (Figure S3A–C). The ENV mice exhibited elevated frequency of Tfh cells (Figure S3D, E) but only slightly elevated serum IgA or IgG levels (Figure S3F, G). However, we observed increased IgA coating of ENV fecal microbiota (Figure S3H, I). Protein-level validation via flow cytometry and CyTOF confirmed the RNA findings (Figure 2C-H, Figure S3A–C). 16S rRNA analysis of IgA-bound bacteria showed strong environment-dependent differences. In CNV mice, the IgA⁺ fraction was dominated by Bacteroidia, whereas environmental exposure (ENV) broadened IgA targeting to include Clostridia and Bacilli (Figure S3J). ENV/AUTO mice formed a distinct cluster, indicating that non-viable environmental components are sufficient to alter IgA specificity. Beta diversity analysis confirmed clear separation between groups, with the environment explaining most of the variance (Figure S3K). These shifts coincided with increased germinal center B cells, IgA⁺ B cells, and Tfh cells, and elevated serum and fecal immunoglobulins in ENV mice (Figure S3A–H). Together, these data demonstrate that environmental exposure reshapes IgA targeting of the microbiota in parallel with enhanced humoral immunity, and that microbial viability is not required for this effect.

Adaptive cells in ENV mice showed CD44 (Figure S4A, B) and Nur77^GFP^ upregulation (Figure 2I, J, Figure S4D), a marker of TCR/MHC engagement,^16^ while ENV/AUTO mice showed only mild activation, highlighting the importance of constant contact with live microbial antigens.^37^ ^,^ ^38^ FACS analysis revealed expansion of CD11b⁺ myeloid cells, including Ly6C^low^ monocytic and Ly6C^high^ granulocytic subsets, and CD11c⁺ dendritic cells (Figure 2G, H). Both RNA and protein analyses showed marked expansion of immunosuppressive populations: Tregs, Tr1 cells, and IL-10⁺ or Arg-1⁺ CD11b⁺ cells in ENV but not ENV/AUTO mice (Figure 2A–D; Figure S4E, F). While suppressor cells are expected in gut immunity to maintain tolerance,^39-41^ their expansion in ENV mice was striking. Microbiota analysis (Figure 1C, D; Figure S1E) revealed enrichment of *Bacteroides*, *Clostridia*, and *Verrucomicrobia*, which are known to promote Treg induction.^42-44^ Our recent work showed peripheral Tregs (pTregs) often arise from anergic CD4⁺Foxp3⁻ cells, a process facilitated by Tregs and *A. muciniphila*.^45-47^ Consistent with this, ENV mice had increased anergic helper T cells (CD4⁺CD44⁺Foxp3^hCD2⁻^FR4⁺CD73⁺ Tan) (Figure S4G). ENV Tregs expressed higher levels of IL-10, TGF-*β*, and the ectoenzymes CD39 and CD73, which degrade ATP to AMP and adenosine.^48^ Detailed gene expression and clustering data are presented in Table S1.

### Peripheral ENV adaptive but not innate immunity is underrepresented

Unexpectedly, ENV mice exhibited significantly smaller mesenteric lymph nodes (mLN) and spleens with reduced cellularity compared to CNV controls (Figure 3C–F). Transcriptomic and protein-level analyses revealed a marked reduction in adaptive immune cells and a concurrent increase in innate populations in ENV, but not ENV/AUTO, mice (Figure 3A–F, Figure S2A–F). ENV mLN cells showed decreased expression of Cd3e, Cd4, Cd8, and Cd19, alongside increased Itgax, Itgam, and Ly6c transcripts – confirmed by FACS – relative to CNV mice. Despite their reduced adaptive cell numbers, ENV mice displayed increased T cell activation, with TCR-specific signaling confirmed by pLck and Nur77^GFP^ expression (Figure S4A–D). Importantly, tolerogenic and anti-inflammatory populations, including Tregs, Tr1 cells, B220⁺IL-10⁺ B cells, and IL-10⁺/Arg-1⁺ CD11b⁺ myeloid cells, were expanded in peripheral lymphoid organs, mirroring changes in the colonic lamina propria (Figure S4D, E). Gene Ontology (GO) analysis (Figure S2G) revealed significant differences in cellular processes between CNV and ENV mice, indicating broad molecular and cellular remodeling. Network-level pathway analysis of the mLN transcriptomes (Figure S2H) revealed that environmental microbial exposure does not merely increase immune activation, but also profoundly reorganizes immune signaling architecture. CNV mice differ from environmental animals by coordinated changes in innate sensing, antigen presentation, and T cell differentiation pathways. Notably, the ENV/AUTO versus ENV comparison showed smaller yet biologically meaningful alterations characterized by immune regulation and homeostatic processes, suggesting that stable autochthonous microbiota refine rather than amplify immune responses. Pathway-level network analysis indicates that environmental housing alone (CNV→ENV/AUTO) induces substantial immune priming, characterized by changes in antigen presentation, leukocyte activation, and metabolic programs. In contrast, exposure to live environmental microbiota (CNV→ENV) results in additional qualitative remodeling of the immune signaling architecture, with coordinated integration of innate sensing, antigen processing, and adaptive immune pathways.

Together, these findings suggest that although adaptive immunity is quantitatively reduced in the peripheral tissues of ENV mice, there is a compensatory expansion of regulatory and tolerogenic subsets. This shift likely reflects a need for enhanced peripheral tolerance in response to continuous microbial stimulation.

### ENV microbiota enhances tolerogenic, anti-inflammatory immune cells

The unexpected shift in adaptive and innate immune cell proportions in ENV mice prompted us to examine *in situ* proliferation. T and B cells in ENV mice showed reduced proliferation compared to CNV controls, while innate cells had higher CD71 and Ki-67 expression (Figure S4H, Table S1). Consistently, mTOR activity was lower in ENV CD4⁺ T cells but elevated in CD11b⁺ innate cells (Figure S5A). We hypothesize that reduced adaptive cell proliferation serves to (1) prevent excessive immune activation and (2) allow space for innate cells to respond to novel microbes. The expansion of immunosuppressive populations in ENV mice supports this model, particularly the increase in IL-10⁺ Tregs, which likely promotes tolerance to microbial fluctuations (Figure S4E, F). The enhanced T cell anergy in ENV mice further supports this tolerogenic shift (Figure S4G).^21^ ^,^ ^49^ Gene and protein analyses revealed elevated CD39 and CD73 expression on ENV Tregs (Table S1), which are molecules linked to metabolic regulation and suppressive function.^48^ ENV CD4⁺Foxp3⁺ cells also expressed higher levels of connexin 43 (Cx43) (Figure S5B), a gap junction protein involved in Treg lineage commitment and effector cell anergy.^21^ ^,^ ^50^ Given Tregs’ elevated adenylate cyclase (Adcy) and reduced phosphodiesterase 3 (Pde3) expression,^51^ ^,^ ^52^ we assessed these factors in FACS-sorted CD4⁺Foxp3^hCD2⁺^ cells. ENV Tregs expressed higher *adcy4* and *adcy7* and lower *pde3*, indicating increased intracellular cAMP (Figure S5B). Functional suppression assays confirmed the superior suppressive capacity of ENV Tregs (Figure S5C). Blocking IL-10 or Cx43 partially impaired ENV Treg function but completely abolished CNV Treg suppression (Figure S5D), suggesting redundancy or compensatory mechanisms in ENV cells. Importantly, the CD28 superagonist used to expand murine Tregs ^42^ did not work for ENV Tregs (Figure S5E), similar to reported studies.^9^ ENV CD11b⁺ myeloid cells also exhibited greater suppressive capacity than their CNV counterparts (Figure S5F), expressing higher levels of Arg-1, IL-10, ROS, superoxide, and iNOS (Figure S5G–I), with no change in the mitochondrial potential (Figure S5J). These features suggest enhanced antimicrobial and immunosuppressive functions.^43^ ENV CD11b⁺ cells, which are largely dependent on IL-10, as neutralization abolished suppression (Figure S5H).

In summary, our data suggests that dirt microbiota induced robust expansion of tolerogenic and anti-inflammatory immune populations, particularly IL-10-producing cells, reshaping immune responses toward enhanced peripheral tolerance. Notably, microbial antigens alone (ENV/AUTO) were sufficient to reprogram the host’s immune tone.

### Innate and adaptive immune precursors are unaffected in ENV mice

To investigate the cause of peripheral immune shifts in ENV mice (Figure 3), we examined T and B cell maturation, precursor abundance, and lineage commitment. The frequencies of thymic single-positive (SP), double-positive (DP), and double-negative (DN) precursors were similar between the groups (Figure S6A). Although peripheral dendritic cells (DCs) may carry microbial antigens to the thymus,^44^ we did not observe increased thymic myeloid cells in ENV mice. Additionally, the proportions and expression levels of CD4⁻/CD8⁻ EpCAM⁺MHCII⁺CD80⁺ thymic epithelial cells were comparable between the groups (Figure S6B). Owing to their low abundance, subtle changes in thymic DCs may have been missed.^53^ Given the peripheral expansion of ENV Tregs, we assessed their origin but failed to observe changes in thymic SP CD4⁺CD24^low^Foxp3^hCD2⁺^ Treg frequencies (Figure S6C), suggesting that microbiota exposure does not affect thymic Treg development. Similarly, bone marrow populations – including Sca-1⁺, c-Kit⁺, CD38⁺IgM⁺, and B220⁺ cells – were comparable between ENV and CNV mice (Figure S6D), ruling out altered precursor abundance as a cause of peripheral adaptive cell reduction.

In summary, adaptive immune precursors remain intact in ENV mice, suggesting that peripheral immune shifts are driven by local antigen presentation rather than central developmental changes.

### Reduced peripheral ENV adaptive immunity is driven by enhanced intestinal translocation

We discovered that ENV mLN T cells showed increased expression of the gut-homing markers α4β7, CCR9, and CCR10, along with elevated CCL5 (Table S1). Following FTY720 treatment (a sphingosine-1-phosphate receptor 1 (S1PR1) agonist ^54^), flow cytometry revealed ~3-fold more T cells in the blood of ENV mice compared to CNV controls at 16 h post-injection. Notably, 52 ± 7.3% of ENV TCRβ⁺ cells expressed gut-homing markers, whereas 17.9 ± 4.6% in CNV mice (Figure S6E, F). These findings support a model in which peripheral adaptive cells in ENV mice recognize microbial antigens presented by DCs, become activated in the mLN, and rapidly migrate to the cLP. This likely contributes to reduced peripheral adaptive cell numbers (Figure 3B-F). These traits were absent in ENV/AUTO mice, indicating that live, replicating microbiota are essential for driving this translocation. While autoclaved dirt contains sufficient antigenic material to induce modest immune changes (Figure 3), it fails to trigger robust T cell migration to the gut, suggesting that only live microbes provide adequate antigenic stimulation.

### Dirt microbiota reprograms metabolism of ENV animals

Given the observed differences in immune cell proliferation (Figures S4H and S5A), we investigated how the environmental microbiota influences cellular bioenergetics. As previously shown, ENV Tregs exhibit enhanced suppressive function, driven in part by elevated cAMP, adenosine production, and intercellular communication (Figure S5B–D). To further characterize these changes, we employed Met-Flow, a flow cytometry-based platform for metabolic pathway analysis ^30^ (Figure S7). CD4⁺ T cells from ENV mice showed altered glycolysis (GLUT1), oxidative stress (PRDX2), TCA cycle (IDH2), and electron transport chain (ATP5A1) activity, which was consistent with reduced mTOR signaling and proliferation (Figures S7, Figure S5A; Figure S4H). ENV Tregs displayed increased oxidative phosphorylation, antioxidant capacity, and fatty acid oxidation (FAO), supporting their stable, suppressive phenotype. CNV naïve CD4⁺ cells exhibited low glycolysis, FAO, and TCA activity, reflecting a quiescent state. In contrast, ENV naïve CD4⁺ cells showed elevated GLUT1, suggesting mild tonic activation by environmental microbes. Activated CNV CD4⁺ cells relied primarily on glycolysis and the pentose phosphate pathway (PPP), whereas ENV counterparts utilized FAO and TCA activity to sustain effector function. ENV CD8⁺ cells showed reduced glycolysis, TCA, and electron transport chain activity, correlating with lower proliferation but higher activation, even within the CD44⁻ subset (Table S1). Innate immune cells in ENV mice, which proliferated more than CNV counterparts, exhibited enhanced glycolysis and TCA cycle activity. However, electron transport chain components (ATP5A1, ASS1) were downregulated. CNV-innate cells favor glycolysis, which is consistent with an M1-like inflammatory profile, while ENV innate cells expressed markers of an M2-like phenotype (IDH2, PRDX2, and ATP5A1), indicating a shift toward oxidative metabolism and redox balance. The improved mitochondrial activity in ENV innate cells further supported this reprogramming.

In conclusion, environmental microbiota exposure reshaped immune cell metabolism, shifting from glycolysis-dominant, hyperreactive states to FAO- and oxidative phosphorylation-driven profiles with enhanced redox control. These changes align with reduced adaptive cell proliferation and support immune tolerance. This metabolic reset may help mitigate hyperinflammation (e.g., sepsis), chronic adipose inflammation (e.g., obesity/diabetes), and mitochondrial dysfunction during aging (inflammaging). If the microbiota can re-tune these programs, they may offer therapeutic potential to restore metabolic balance and slow immune decline.

### Housing mice in dirt modulates intestinal inflammation

**Figure 4.**
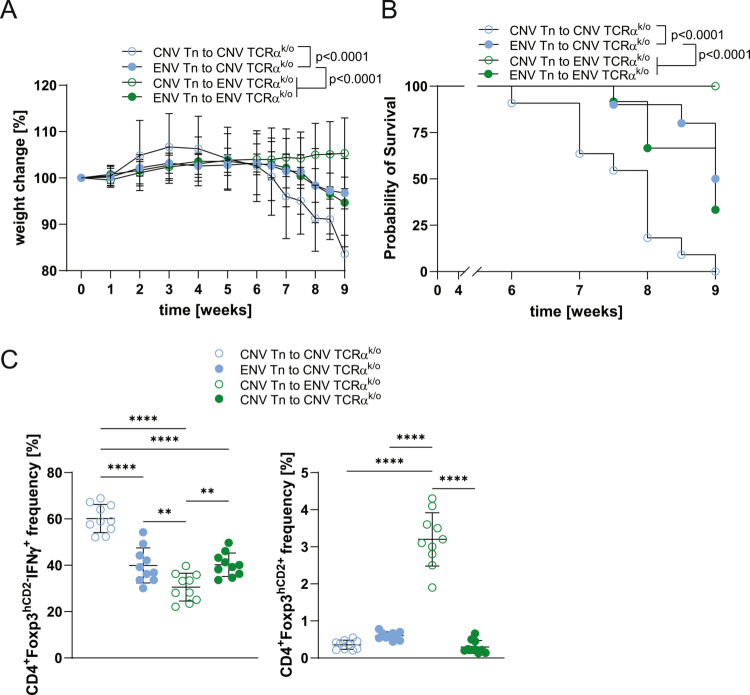
Housing of TCRα^k/o^ recipient mice in dirt induces a tolerogenic CD4^+^ cells in the CD4^+^CD45RB^hi^ colitis model. (A) Weight change of CNV and ENV TCRα^k/o^ mice transferred with FACS-purified naïve CNV or ENV CD4^+^ cells (Tn) from B6^Tg^ mice. (B) Kaplan‒Meier survival curves for the mice (*n* = 10/group) presented in (A). (C) The ENV environment induces fewer pro-inflammatory cytokines and more pTregs measured by FACS in cLP CD4^+^ cells. The animals were analyzed 9 weeks post-transfer. Each symbol in (C) indicates an individual animal. **p < 0.01, ****p < 0.0001 one-way ANOVA followed by Tukey’s post-hoc test.

To assess how the intestinal environment in ENV mice influences inflammation, we used the CD4⁺CD45RB^high^ chronic colitis model with CNV and ENV TCRα^k/o^ lymphopenic mice as recipients of naïve CD4⁺ cells (CD4⁺CD45RB^high^CD44⁻Foxp3^hCD2⁻^; Tn) and monitored the mice for nine weeks. CNV recipients exhibited greater weight loss and required euthanasia earlier than ENV recipients (Figure 4A, B). Colon length, weight, and histology confirmed more severe inflammation in CNV mice (Figure S8A–D). Flow cytometry revealed increased IFNγ and IL-17, and reduced IL-10 in CD4⁺ cells from CNV cLP, while ENV recipients showed induction of IL-10-producing peripheral Tregs (Figure 4C; Figure S8E). In complementary experiments, the transfer of ENV naïve CD4⁺ cells into CNV and ENV TCRα^k/o^ mice resulted in milder inflammation, lower IFNγ, and higher IL-10 production compared to CNV-to-CNV transfers (Figure 4; Figure S8). While the ENV environment tends to induce Tfh cells (Figure S8F, G), driving non-significant but noticeable IgA induction (Figure S8H), the level of circulating IgG was significantly reduced in ENV hosts receiving CNV naïve CD4^+^ cells, which is consistent with a reduction of colitis (Figure S8I).

In summary, the colonic microenvironment in ENV mice promotes immunomodulatory cell development. ENV-derived naïve CD4⁺ cells confer partial protection against inflammation when transferred into CNV lymphopenic hosts, highlighting the anti-inflammatory potential of microbiota-conditioned immune cells.

### Environmental Gram-negative bacteria drive anti-inflammatory immunity in ENV mice

The chronic colitis model is entirely microbiota-dependent, as germ-free mice are protected from disease.^55^ To identify microbial drivers of the ENV immune phenotype, we treated mice with antimicrobials targeting Gram-negative bacteria (ceftazidime), Gram-positive bacteria (vancomycin), or fungi (fluconazole + 5-fluorocytosine + amphotericin B), individually or in combination. Depletion of Gram-negative bacteria alone or in combination – but not Gram-positive bacteria or fungi – significantly altered immune cell frequencies, resembling CNV profiles (Figure S9A). This indicates that Gram-negative microbes are key contributors to the ENV immune phenotype. The outcome of the fecal microbiota transfer (FMT) experiments further supported this conclusion (Figure S9B, C). Given prior reports that Gram-negative bacteria *Akkermansia muciniphila*, promote tolerogenic immunity,^19^ ^,^ ^45^ we tested whether supplementation could restore the ENV phenotype in ceftazidime-treated mice. Reintroduction of *Akkermansia* modestly increased Tregs (Figure S9D) and partially rescued mice in the colitis transfer model (Figure S9E). Given the complexity of dirt microbiota, it is likely that Gram-positive bacteria and fungi also contribute through competitive or mutualistic interactions to drive the ENV phenotype (see also Figure S11).^7^ ^,^ ^10^ ^,^ ^33^ ^,^ ^56^

### Environmental microbiota protects against sepsis via IL-10-mediated immune modulation

**Figure 5.**
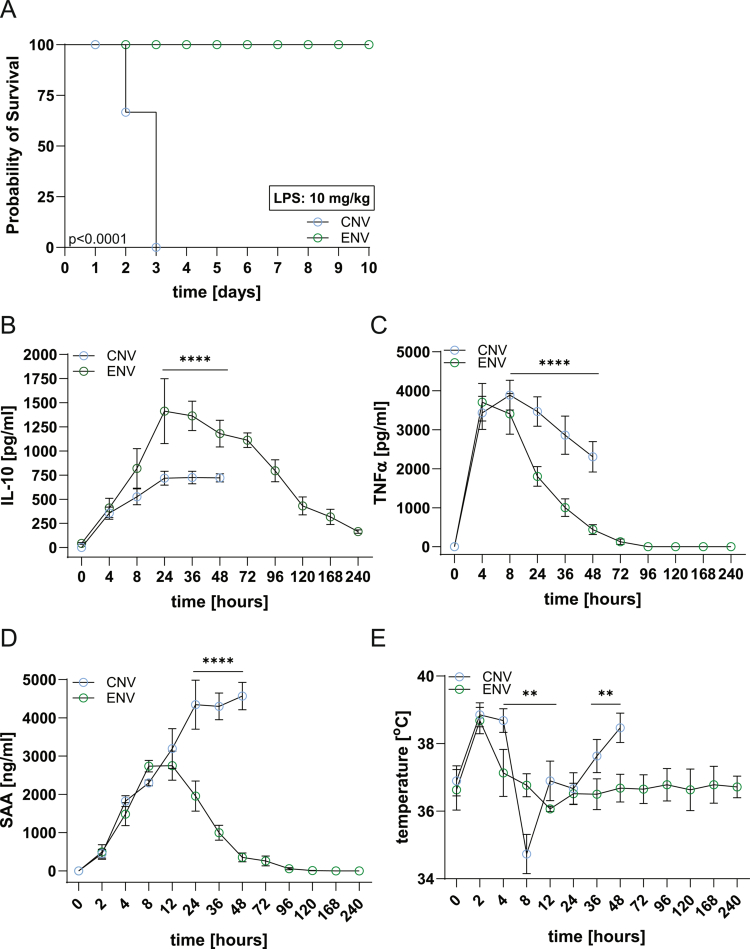
ENV immunity protects the host from sepsis. Sixteen-week-old CNV and ENV mice were injected with a lethal dose (10  mg/kg) of LPS. (A) Survival is shown for both groups. (B-E) Analysis of serum cytokine (IL-10 (B), TNFα (C)), SAA (D) expression, and animal temperature (E) at the indicated time points is shown. The experiment was repeated twice with a total of *n* = 8 mice/group. **p < 0.01, ****p < 0.0001 one-way ANOVA followed by Tukey’s post-hoc test.

Given the expansion of IL-10⁺ immune cells in ENV mice (Figure S4E, F) and their distinct metabolic profile (Figure S7), we tested their response to lipopolysaccharide (LPS), a model of Gram-negative bacterial infection and sepsis.^57-61^ LPS clearance depends on IL-10, which is transiently induced by low-dose exposure and known to confer protection.^62^ ^,^ ^63^ Since ENV mice express elevated IL-10 driven by PRDX2, CPT1A, and ATP5A1 (Figure S7C, E, F), we hypothesized that they would be more resistant to LPS toxicity. Indeed, all ENV mice survived a lethal LPS dose (10  mg/kg), while all CNV controls succumbed within 72 hours (Figure 5A). LPS triggered IL-10 release in both groups, but ENV mice produced significantly more, effectively suppressing TNFα and serum amyloid A (SAA), a CRP analog ^64^ (Figure 5B–D), and more effectively suppressing the pyrogenic effects of TNFα (Figure 5E). TLR4 expression was comparable between the groups (Figure S10I), indicating that differential receptor signaling was not responsible. Reduced IL-10 in CNV mice correlated with lower CD4⁺ cell numbers in the mLN, a loss not observed in ENV mice (Figure S10A), despite their higher initial activation (Figure S10B). The increased expression of Nur77 in ENV CD4^+^ cells (Figure S4D) likely contributes to this finding, as it was shown that Nur77 protects CD4⁺ cells from TNF-induced apoptosis.^65^ Both Tr1 and Treg IL-10⁺ CD4⁺ cells expanded in response to LPS, but were more robust in ENV mice (Figure S10C, D). The ENV mice showed increased IL-10⁺ CD11b⁺ cells but reduced total CD11b⁺ cell numbers (Figure S10E, F), suggesting that Tr1 and Tregs were the primary mediators of protection. ENV CD11b⁺ cells also cleared LPS more efficiently (Figure S10G, H).

In summary, ENV mice were protected from LPS-induced sepsis. Mechanistically, IL-10–producing CD4⁺ cells resisted toxin-induced deletion, which was supported by a metabolic program involving AMPK-PPARα/PGC-1α-driven FAO/OxPhos (CPT1A, ATP5A1), PRDX2, IDH2, and NADPH buffering. This pathway suppresses glycolytic NF-κB/mTORC1 signaling and prevents ROS-mediated cell death.^66^ These findings underscore the potential of the environmental microbiota to modulate immune responses and protect against systemic inflammation.

### ENV Gram-negative bacteria modulate immune function in sepsis

Since LPS originates from Gram-negative bacteria (and we previously showed that these microbes drive the ENV immune phenotype and function (Figure S10)), we tested LPS responses in ENV mice depleted of Gram-negative bacteria (ENV^ΔG⁻^) (Figure S11A). ENV^ΔG⁻^ mice were highly susceptible to LPS toxicity, but partial protection was restored by fecal microbiota transfer (FMT) from ENV ENV^ΔG^⁺ donors (Figure S11A, B). In contrast, supplementation with *A. muciniphila*, despite its known ability to expand IL-10⁺ Tr1 and Treg cells,^19^ ^,^ ^45^ failed to rescue ENV^ΔG⁻^ mice but modestly modulated TNFα and mouse temperature (Figure S11B–F). These results suggest that protection requires a diverse microbial community capable of fine-tuning immune responses, and that this protection is transferable via the colonization of CNV hosts with the ENV microbiota. Sensitization with low-dose LPS also improved survival (Figure S11), which is consistent with previous reports.^67^

In summary, our data shows that: (1) microbial synergy is essential for optimal host protection from sepsis, and (2) the environmental microbiota tunes host immunity to mitigate infection and endotoxemia.

### Environmental microbiota protects against obesity and diabetes

**Figure 6.**
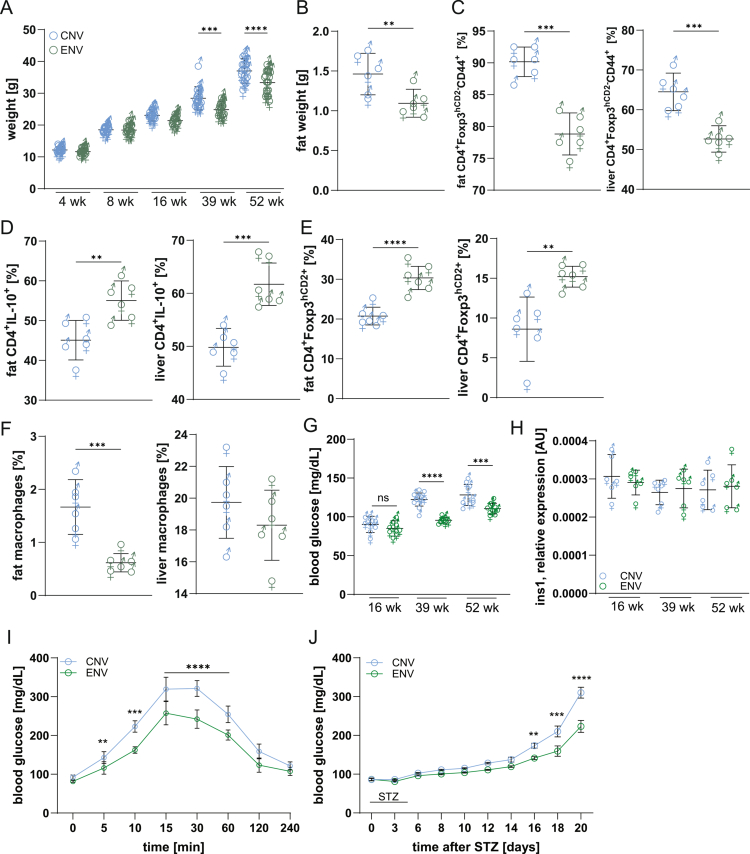
ENV mice are resistant to aging-related conditions. (A) Weight gain over time is reduced in ENV mice compared to CNV animals. (B) Fat weight of the indicated mice. (C-F) CD4^+^ cells in the fat and livers of ENV mice show lower cell activation (C) and a higher proportion of anti-inflammatory IL10^+^ (D) and Treg (E) cells and lower frequencies of pro-inflammatory macrophages (F). (B–F) Data was generated from 52-week-old mice. (G–J) The ENV microbiota regulates glucose metabolism. (G) Blood glucose was measured in CNV and mice at the indicated age. (H) The expression level of Ins1 is shown for both types of mice. (I) Glucose tolerance test in 52-week-old mice (*n* = 6/group). Glucose levels were measured at the indicated time point. (J) Streptozotocin (STZ) diabetes model shows higher resistance to chemically induced diabetes in ENV mice (*n* = 8/group). For (A-H), each symbol indicates a separate mouse. **p < 0.01, ***p < 0.001, ****p < 0.0001 one-way ANOVA followed by Tukey’s post-test.

ENV mice, regardless of sex, were resistant to weight gain compared to CNV controls (Figure 6A). No differences in cage temperature, bedding thermal properties, or animal activity were observed, and both groups maintained similar body temperatures (personal observation). These findings point to microbiota-driven modulation of host physiology. ENV mice exhibited reduced abdominal fat and fewer pathogenic IFNγ⁺ T cells, alongside expanded Tregs in the fat and liver (Figure 6B–F). Reportedly, the age-related decline in Treg function contributes to diabetes,^50^ and aging impairs insulin clearance.^51^ However, ENV Tregs are more metabolically and functionally robust (Figures S5C and S7), suggesting a protective role. Fasting glucose levels were significantly lower in ENV mice regardless of age (Figure 6G), with no difference in pancreatic *Ins1* expression (Figure 6H), ruling out altered insulin production. Glucose tolerance tests showed improved clearance and lower peak glucose in ENV mice (Figure 6I). Upon streptozotocin (STZ) challenge, ENV mice were less susceptible to diabetes induction (Figure 6J).

In summary, the environmental microbiota reprogram immune cells toward oxidative and antioxidant metabolic states, reducing inflammation and promoting tissue repair and tolerance. These adaptations enhance resistance to metabolic disease, support immune balance, and promote healthy aging.

### Environmental microbiota promote host lifespan and health

**Figure 7.**
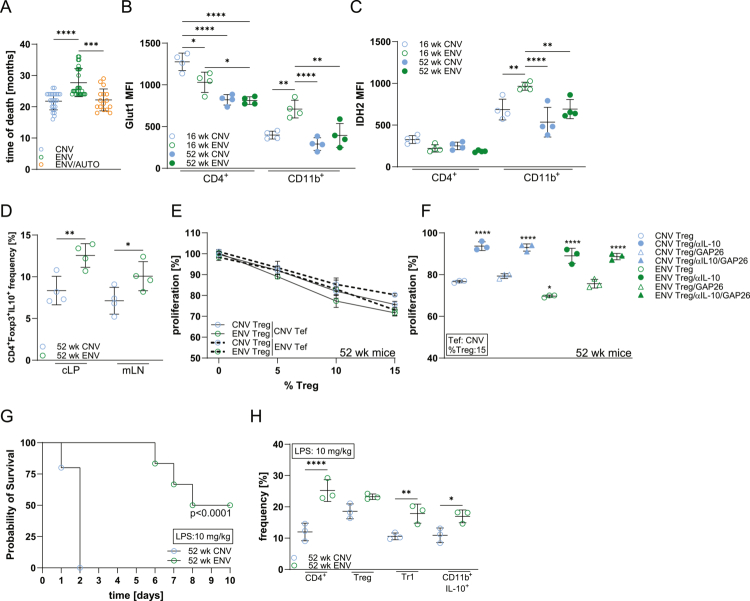
Aged ENV mice supersede their CNV counterparts. (A) Spontaneous time of death is shown for CNV, ENV, and ENV/AUTO mice. (B) Expression of Glut1 (glycolysis) and IDH2 (TCA cycle) (C) is affected in adult but not aged ENV mice compared with their CNV counterparts. (D–F) ENV Tregs maintain higher frequency (D) and suppressive function in aged animals (E, F). (G, H) Aged ENV model mice are far more resistant to lethal LPS compared with aged CNV animals (*n* = 4/group) by sparing IL-10-producing immune cells. Each symbol represents a separate animal. *p < 0.05, **p < 0.01, ***p < 0.001, ****p < 0.0001 one-way ANOVA followed by Tukey’s post-test.

ENV mice exhibited increased lifespan compared to CNV and ENV/AUTO controls (Figure 7A). Aging is associated with increased immunosuppressive cells, particularly Tregs, as a compensatory response to chronic inflammation.^68^ ^,^ ^69^ However, these cells often exhibit reduced suppressive function in both healthy and autoimmune contexts.^50^ ^,^ ^70^ ^,^ ^71^ Combined with thymic involution, peripheral tolerance mechanisms attempt to restrain inflammatory responses. Our data indicate similar glycolytic (Glut1) and TCA cycle (IDH2) activity in aged ENV and CNV mice, despite being markedly different in younger animals (Figure 7B, C; Figure S7B, D). This may reflect increased metabolic activity in aged ENV T cells rather than a decline in CNV cells, which is supported by stable innate cell numbers and elevated Tr1 and Treg populations in aged CNV and ENV mice, with increased frequencies and suppressive function of the latter (Figure 7D–F). Importantly, aged ENV mice were protected from lethal LPS challenge (Figure 7G, H).

In summary, the ENV microbiota seems to more effectively modulate age-related inflammation (inflammaging), promoting immune resilience and longevity through elevated Treg numbers and suppressive function.

## Discussion

Despite its simplicity, the ENV model produced robust and reproducible results, confirming prior findings,^4^ ^,^ ^9^ ^,^ ^72^ and revealing novel insights. ENV mice mirrored traits seen in wild microbiota models, including robust displacement of SPF strains, immune reshaping, and tolerogenic polarization of immune cells ^33^ ^,^ ^72^ or lack of Treg expansion in the CD28 superagonist (CD28SA) model.^9^ Unique features included expansion of IL-10⁺ Tregs, Tr1 cells, MDSCs, and M2 macrophages-driven by local soil microbes and in the absence of native for rodents pathogenic strains,^5^ ^,^ ^9^ pointing to unique, tolerance-driving microbiota found in Georgia. These microbial shifts had functional consequences: soil-colonized microbiota ENV mice live longer, remain leaner, show improved glucose control, resist colitis and sepsis. These benefits were absent in mice exposed only to heat-killed microbes, underscoring the importance of the live microbiota. The removal of Gram-negative microbes abolished most of the benefits, and while colonization with *A. muciniphila* alone was insufficient to restore the full phenotype, it was able to mitigate certain symptoms of chronic diseases, supporting the need for diverse microbial communities.^9^ Our findings parallel human epidemiological data: in industrialized and urban regions where environmental microbial exposure is limited, chronic inflammatory conditions such as obesity, diabetes, and inflammatory bowel disease are increasingly prevalent – impacting quality of life and imposing economic burdens.^73-75^ Dysbiosis contributes to those modern chronic diseases that were all mitigated in ENV mice through the expansion of IL-10⁺ immune cells and enhanced removal of LPS by innate cells. We discovered that peripheral adaptive cells were underrepresented in ENV vs CNV mice and established that it was due to more efficient intestinal translocation of CD4^+^ cells driven by the upregulation of gut-homing markers. Likewise, ENV mice had intestinal IgA^+^ and ILC3 cells expanded in response to novel, dirt-derived microbiota modulating microbes and affecting intestinal barrier integrity. Antibiotics reduce short-chain fatty acids (SCFAs), key microbial metabolites that regulate energy balance, immune function, and inflammation,^76^ and the removal of Gram-negative bacteria affects the phenotype, frequency, and function of ENV immune cells. Loss of *A. muciniphila*, a common feature in metabolic disease, is linked to worsened outcomes,^77-80^ and restoring microbial diversity improves inflammation and metabolism. Based on known bacteria‒host interactions and ENV microbiota composition (Figure 1; Figure S1), we hypothesize that *Clostridia* (SCFA producers), *Bacteroides* (low-agonist LPS), bile acid metabolizers, *Akkermansia*, and *Lactobacilli* contribute metabolites and TLR signals that promote FAO/OxPhos, antioxidant capacity, and IL-10 production. These features protect ENV CD4⁺ cells and restrain myeloid-driven cytokine storms during LPS-induced sepsis, highlighting the role of bacteria in modulating immune toxicity.^81^ ^,^ ^82^

While the influence of the microbiota on host physiology is well established, the reciprocal impact of the host on its microbiome remains underexplored. Our data show that colonization with environmental microbes alters immune cell composition, which in turn shapes the gut microbiota, especially during aging. Even short therapeutic interventions, such as anti-tumor treatments, depend on specific microbial profiles, influencing outcomes such as tumor rejection, autoimmunity, and colitis.^83^ Emerging tools, including microbiome Genome-Wide Association Studies (GWAS) ^84^ ^,^ ^85^ and AI-based deep learning approaches,^86^ now enable high-resolution mapping of host‒microbiota interactions. These technologies, combined with environmental animal models, will be essential for unraveling the complex, dynamic, and reciprocal relationship between host immunity and microbial ecology.

However, differences between environmental models must be carefully considered. While ENV mice were completely protected from LPS-induced sepsis (mainly due to CD4^+^IL-10^+^ cells resisting toxin-induced deletion by switching their metabolism to FAO/OxPhos), “wildlings” were reported to show the opposite effect,^9^ which we attributed to differences between the microbiota of these two models. A recent comparative analysis ^87^ summarized the strengths and limitations of various environmental mouse models, highlighting their utility in specific contexts, such as infection or allergy, indicating the importance of the local microbiota in shaping immune outcomes. Soil is the most biodiverse habitat on Earth,^88^ and recent work by Rodriguez del Rio et al. ^89^ demonstrated that global change factors – such as temperature shifts, nitrogen deposition, microplastics, herbicides, insecticides, and antibiotics – can alter soil properties and microbial diversity, which in turn can alter microbial balance and impact host immunity.^7^ ^,^ ^9^ Notably, fungicide treatment leads to a dramatic increase in bacterial biomass due to reduced fungal competition, illustrating the delicate balance between microbial species in both the environment and host.^33^ ^,^ ^87^ Although fungi play a minimal role in ENV immunity, other models have shown strong immune activation by *Candida* species.^7^ ^,^ ^10^ Microbial interactions (such as cross-feeding, metabolic plasticity, and horizontal gene transfer) further shape colonization dynamics and immune responses.^90-92^ Despite their translational potential, environmental models face reproducibility challenges owing to geographic microbiota variability, similar to SPF models. Host genetics also influences outcomes: rewilded B6, 129, and PWK/PhJ mice responded differently to *Trichuris muris* infection, with genotype affecting parasite burden but not immune composition.^93^ ^,^ ^94^ Environmental exposure modulates susceptibility, while genetic factors shape immune cell traits such as CD44 expression ^4^ (Figure S4A, B). Many intestinal T cells recognize conserved microbial proteins, such as ATP-binding cassette transporters, which can induce both Tregs and Th17 cells.^95^

ENV mice establish a gut microbial ecosystem that more closely reflects key features of rural and non‑industrialized human populations than urban cohorts. Sustained environmental microbial exposure increases community richness and functional redundancy, counteracting the reduced microbial transmission, antibiotic perturbation, and dietary simplification characteristic of urban living, which are associated with the loss of ancestral microbial functions and immune hypo‑education. Importantly, ENV exposure promotes a more tolerogenic immune tone, which is consistent with the microbiota‑driven mucosal regulation observed in rural human populations. These pathways support microbial containment while limiting excessive inflammation, paralleling human data linking high environmental microbial exposure to immune calibration rather than immune suppression. Thus, ENV mice provide a mouse‑adapted approximation of a microbiota shaped by sustained environmental exposure that supports immune tolerance, maturation, and resilience across the lifespan.^96-100^

The ENV system, despite its simplicity, proved robust in deciphering host‒microbiota interactions, demonstrating that local environmental microbes strongly influence the immune system and revealing differences between ecological niches. While this partially explains the limited reproducibility of studies across institutions – an important consideration for scientific rigor – it also underscores a fundamental principle: the goal of research is not mere replication, but to generate insights into human health and disease. Such insights must account for the inherent variability driven by numerous individual differences, including exposure to diverse environmental microbiomes. Nonetheless, natural colonization in ENV and wildling mice offers immunological parallels to humans and may be particularly valuable for studying both adaptive and innate memory responses.^60^ ^,^ ^101-103^ In this context, the ENV model functions as a translational discovery platform to identify immune programs and microbial features that promote tolerance and disease resilience, rather than as a source of direct microbial therapy. We demonstrate that ENV-induced tolerogenic immunity requires Gram-negative bacteria, as their depletion abolishes this effect, with *Akkermansia muciniphila* emerging as one key driver. These findings illustrate how ENV exposure can be leveraged to pinpoint defined, clinically relevant taxa and microbial functions that shape protective immunity, even in the context of subsequent dysbiosis. By linking environmental microbial diversity to mechanistically defined immune pathways, ENV provides a framework to bridge preclinical studies with rational prioritization of candidate microbes or microbial functions for evaluation in human inflammatory, metabolic, and septic disease.

### Limitations and future directions

This study focused exclusively on the environmental microbiota, without addressing dietary influences, an important factor in shaping microbial composition and host metabolism. Future work should explore how diet (“human” diet consisting of vegetables, fruits, seeds, and animal proteins (e.g., in the form of mealworms); Western diet, etc.) interacts with environmental microbes to modulate immunity and metabolic health. A limitation of this study is that environmental exposure was derived from soil collected in Georgia; however, sequencing of soils from multiple U.S. locations revealed substantial conservation of microbial community structure at the phylum level, with consistent dominance of Proteobacteria, Actinobacteria, Acidobacteria, and Firmicutes despite geographic variation in relative abundances (Figure S1H). These regional shifts occur within a shared core environmental microbiome and are consistent with known effects of local climate and land use. Accordingly, the immune phenotypes observed in environmental mice – reflecting enhanced immune maturation and altered inflammatory responsiveness – are best interpreted as consequences of exposure to a complex, diverse environmental microbial community rather than to site-specific taxa unique to Georgia soil. Nonetheless, soils from distinct global biogeographical regions harbor different microbial assemblages that may differentially influence immune education, and extending this model to “non-moderate” environments will be important to fully define the global generalizability of these findings. Future studies should be expanded to aim at dissecting specific microbial contributors within the ENV model. A limitation of antimicrobial-based studies is their selective targeting, which can unintentionally shift non-targeted populations, possibly explaining why Gram-negative supplementation alone did not fully restore the ENV phenotype (Figure S11). The scRNAseq design used pooled libraries (one library per condition), which preclude formal per-mouse statistical testing of cluster abundance and pseudo bulk differential expression. We therefore present scRNA-seq results descriptively and ground statistical inference in replicated orthogonal assays. Future work will incorporate per mouse single-cell libraries to enable formal compositional and pseudo bulk analyses. Longitudinal studies of microbiota shifts over time are warranted to better understand their effect on the host’s life expectancy. Similarly, tracing colonization using germ-free mice (with caution regarding potential pathogens that could harm the animals) or tagging dirt-derived microbiota would be informative, as it would enable precise tracking of microbial engraftment, persistence, and host interactions, thereby helping distinguish causal effects from simple associations. In addition to IgA, studying the IgG-coated microbiota would provide deeper insights into taxon-specific immunoglobulin targeting. The functional assessments of liver metabolism and injury, particularly during infection or LPS challenge, remain to be conducted. The tolerogenic immune environment in ENV mice also raises questions about their response to tumors. Given prior evidence that microbiota disruption impairs immunotherapy efficacy – except for CAR T cell therapy^104^ – the ENV model may offer new insights into cancer immunology. Whether environmental microbiota enhance or suppress anti-tumor immunity is an open and testable question.

## Supplementary Material

Supplementary Material Suppl_Tables_GM.pdf

Supplementary Material Supplementary_figure_legends.docx

Supplementary Material Suppl Figs_GM_resubmission.pdf

## Data Availability

All data needed to evaluate the conclusions in the paper are present in the paper and/or the Supplementary materials. Materials are available upon reasonable request and completion of a material transfer agreement (MTA).
