# Supplementary material for: Soil-derived microbiota induces T regulatory cells and protect against mouse colitis, metabolic disease, and sepsis
Source: Gut Microbes. 2026 May 24;18(1):2675089. doi: 10.1080/19490976.2026.2675089 (PMC13203056; doi:10.1080/19490976.2026.2675089)
Supplement: Supplementary Material — Suppl_Tables_GM.pdf [file KGMI_A_2675089_SM3084.pdf]

Table 1. Properties of cLP and mLN cells based on the mouse type (CNV, ENV, ENV/AUTO).

**cLP**	**Mouse Type**	**Key Features**	**Proposed Phenotype**
$CD4^{+}CD44^{-}Foxp3^{-}$ (Tn)	**CNV**	High expression of T cell identity and protein synthesis genes (*Tcf7, Batf, Zfp36*)	Homeostatic naïve $CD4^{+}$ T cells
	**ENV**	Inflammatory signaling and immune regulation (*Ifngr1, Bach2, Hif1a*)	Microbially-modulated $CD4^{+}$ T cells
	**ENV/AUTO**	Stress and effector-prone signature (*Hspa1a, Bcl2l11, Batf*)	Activated/stressed $CD4^{+}$ T cells, possible early effector or autoimmune signature
$CD4^{+}CD44^{+}Foxp3^{-}$ (Tact)	**CNV**	Expression of migration regulators (*Ccr7, S1pr1, Klf2*), survival and anti-apoptotic factors (*Bcl2, Tsc22d3*), and stress responses (*Hspa1a/b, Hspe1*). Mild activation with preserved tissue recirculation capacity.	Migratory, homeostatically activated $CD4^{+}$ T cells
	**ENV**	Regulatory transcription factors (*Bach2, Ikzf1/2, Zbtb20, Nr4a2*), immune modulators (*Cd74, Prkca, Tox*), metabolic plasticity (*Adk, Klf4*), and trafficking genes.	Transcriptionally reprogrammed, immune-tuned $CD4^{+}$ T cells under microbial exposure
	**ENV/AUTO**	Strong inflammatory and effector cytokines (*Ifng, Il17a, Il22*), stress/inflammatory mediators (*Atf3, S100a4/6, Furin, Mapkapk2*), and tissue-associated markers (*Cxcr6, Lamc1, Il1r2*).	Pathogen- or autoantigen-driven effector $CD4^{+}$ T cells with inflammatory bias
$CD4^{+}Foxp3^{+}$ (Treg)	**CNV**	Express genes such as Foxp1, Nfatc1, and Ccr7, which suggest some regulatory capacity and migration potential. However, without high expression of IL-10, CD39, CD73, or CTLA-4, their suppressive activity is likely less robust.	Moderate to low suppressive cells; they lack the key markers of potent suppression seen in other groups.
	**ENV**	The high expression of IL-10, CD39, CD73, and CTLA-4 strongly indicate highly suppressive Tregs.	Well-equipped suppressor cells prone to inhibit inflammatory responses, particularly in tissues prone to immune activation
	**ENV/AUTO**	Express markers such as CTLA-2A, Tnfrsf18, and Gpx4, which suggest enhanced survival and suppression under inflammatory conditions.	Tregs with a generalized regulatory phenotype, possibly focused on Th2-type responses

			and survival under oxidative stress
CD8$^+$CD44$^-$ (CD8 naive)	**CNV**	Primed for immune surveillance and activation, with markers related to T cell migration (e.g., *Ccr7*, *S1pr1*) and potential for rapid response to signals. These cells are likely more quiescent but responsive when needed.	Likely homeostatic, maintaining immune surveillance with quick activation potential.
	**ENV**	Show a pro-inflammatory and cytotoxic phenotype, with markers indicating active effector functions and cytotoxicity (*Gzma*, *Gzmb*). They are highly involved in inflammatory responses and may have a memory-like function, acting quickly to clear infections or control inflammation.	Cytotoxic and effector-driven, designed for responding to acute infections or inflammation.
	**ENV/AUTO**	Involved in regulation and inflammation with high expression of stress response genes (*Hspd1, Hsph1, Gadd45b*, and *Fosb*) and cytokines (*Ccl2, Il1b*, and *Tnfsf18*). These cells might play a role in chronic inflammation, tissue repair, or immune regulation rather than just acute immune responses.	Involved in long-term inflammation or regulation, with a broader role in tissue remodeling and sustained immune responses.
CD8$^+$CD44$^-$ (CD8 act)	**CNV**	*Cish*, *Tnfrsf9* (TNFR family member, suggests involvement in costimulation and activation), *Socs1* (negative feedback on cytokine signaling), *Traf4*, *Icos* (involved in T cell activation and survival). *Tnfsf11* (involved in immune modulation), *Rflnb*, *Bst2* (suggests possible role in immune response modulation or signaling).	Effector memory CD8+ T cells, with a focus on T cell activation, homing, survival, and modulation of immune responses through co-stimulatory and inhibitory signals.
	**ENV**	*Tigit*, *Ccl5* (indicates immune response regulation and chemotaxis), *Gzma* (cytotoxic effector molecule), *Cst7* (suggests involvement in immune modulation). *Rgs1*, *Litaf* (associated with inflammatory responses), *Jun* (transcription factor	Cytotoxic effector phenotype with a strong capability for immune modulation, likely effector CD8+ T cells activated in the context of inflammation or infection.

		indicating stress response), *Fosb* (involved in the activation of immune cells).	
	**ENV/AUTO**	*Tnfsf11*, *Ccl4*, *Ifng* (cytokines suggesting a pro-inflammatory response), *Cebpb* (involved in inflammation and immune cell activation). *Nfil3*, *Ywhae* (involved in transcriptional regulation of immune responses).	Highly activated cytotoxic CD8+ T cells, possibly associated with effector functions in chronic inflammation.
$CD19^+$ (B cells)	**CNV**	Enrichment in heat shock proteins (Hspa1b, Hsp90aa1, Hspd1) suggests cellular stress responses. Plk2, Junb, Pim1, Rasgef1b suggest proliferative and activated B cells. Nfkbia, Nfkbid, Ifi30, Fcer2a, Cd79b point to antigen presentation and B cell receptor (BCR) signaling. Ig-related genes (Igkv1-135, Iglc2) support active immunoglobulin production.	Activated, proliferative, stress-responsive B cells
	**ENV**	Peak1, Zhx2, Fgfr2 suggest metabolic and structural adaptations. Cd86, Cybb, Dock10, Adgre5 suggest B cells with antigen-presenting potential but reduced activation. Crip1, Vim, Rhob indicate a tolerogenic, possibly regulatory phenotype. Ig-related genes (Igkv10-96) indicate some BCR signaling, but not as prominently as CNV.	Tolerogenic B cells with metabolic adaptation
	**ENV/AUTO**	Cd79b, Btg2, Ighm indicate B cell receptor (BCR) activation and memory B cell presence. Nfkb1, Stat4, Kdm6b suggest active transcriptional regulation and inflammation. Egr1, Gadd45b suggest response to stimuli and potential for differentiation. Hsp90b1, Calr indicate antigen presentation	Memory-like B cells with enhanced antigen presentation

		and endoplasmic reticulum stress response.	
CD11c⁺CD11b⁻ (DC)	**CNV**	Macrophages or monocyte-derived cells: *C1qa, C1qb, C1qc (complement components), Csf1r (macrophage marker), Aif1 (macrophage marker), Lyz2 (lysozyme)*, *Msrb1 (reactive oxygen species management)*, *Apoe (lipid metabolism)*, *Clec4n (C-type lectin receptor)*, and *Fermt3 (involved in cell adhesion)*.	Macrophage-like with phagocytic and tissue-repairing roles, reflecting an immune surveillance or homeostasis function
	**ENV**	Dendritic cell or activated macrophage phenotype: *Ccr7 (migratory marker for dendritic cells)*, *Il1r2 (inflammation-related receptor)*, *Ifitm1 (involved in immune response)*, *Il1b (pro-inflammatory cytokine)*, *Foxp1 (transcription factor regulating immune cell function)*, *Syk (tyrosine kinase involved in signal transduction in immune cells)*, *Bst2 (immune response)*, and *Cd274 (PD-L1, immune checkpoint molecule)*.	Dendritic cells with strong antigen presentation, T-cell activation, and inflammatory response capabilities, suggesting they play a role in immune activation.
	**ENV/AUTO**	Inflammatory or cytotoxic dendritic cells, with some overlap with macrophage-like features: *Csf2 (granulocyte-macrophage colony-stimulating factor)*, *Gzma (granzyme A, cytotoxic marker)*, *Tnfrsf18 (TNF receptor involved in immune cell regulation)*, *Rora (transcription factor involved in inflammation and immune response)*, *Map3k5 (involved in cell signaling and inflammation)*, *Gzmc (granzyme C)*, *Mpeg1 (macrophage-expressed gene involved in pathogen defense)*, and *Tnfrsf9 (TNF receptor, immune regulation)*.	Cytotoxic dendritic cell phenotype with heightened inflammation and potential for cytotoxic activity, positioning them as key players in immune modulation and pathogen defense.

CD11c+CD11b+	**CNV**	Tnfsf14 (TNF superfamily, inflammation), Egr1 (immune response), Smad7 (TGF-β signaling inhibitor), Klrb1b (NK cell marker), Ccl4 (chemokine signaling), Cd2 (T-cell activation)	Inflammatory and immune regulatory with cytotoxic properties
	**ENV**	Il22 (epithelial repair), Il10 (immune suppression), Icosl (T cell costimulation), Nlrp3 (inflammasome, immune modulation), Csf1r (monocyte/macrophage regulation), Thbs1 (tissue remodeling), Retnla (anti-inflammatory macrophage marker)	Anti-inflammatory, regulatory, and tissue-repair functions
	**ENV/AUTO**	Klra4/Klra9/Klrb1a (NK receptor family, cytotoxic function), Tnfrsf18 (GITR, Treg and immune activation), Myb (dendritic cell differentiation), Pdgfa (fibrosis, tissue remodeling), Eomes (T-cell cytotoxicity and differentiation), Tcf7 (T cell activation)	Cytotoxic and highly activated antigen-presenting phenotype
CD11c-CD11b+	**CNV**	Immune regulation & trafficking: *Ccr7*, *Cd2*, *Cd69*, *Ptprcap.* Heat shock/stress response: *Hspa1a/b*, *Ddit4.* Antigen presentation & immune modulation: *H2-DMb2*, *Cd72*, *C1qa/b*, *Gimap4.*Transcriptional control: *Ebf1*, *Mef2c*, *Smad7*. Cell metabolism & survival: *Phgdh*, *Eif2ak3*, *Calcrl*, *Pml.* T cell activation & cycle: *Ccnd2*, *Apobec3*	Macrophage-like antigen-presenting cells and B-cell-associated immune regulators, with a balance of inflammation control and adaptive immune communication.
	**ENV**	Regulatory transcription factors: *Tox, Ets2, Ikzf3, Trps1, Setbp1*. Cytoskeletal & signaling adapters: *Zyx, Cnn2, Ypel3, Gngt2, Fis1*. Immune modulation: *Serpinb9b, Nfam1, Cast, GpX1*. Metabolic & oxidative stress response: *Msrb1, Crip1, Osbpl9*. Transcription and RNA processing: *Hist1h1e, Polr2a, Qk*	Higher cytotoxicity potential, antioxidant defense, and stress-response adaptation, suggesting they might be more specialized for immune tolerance and tissue repair.
	**ENV/AUTO**	Strong pro-inflammatory signature: *Il1b*, *Ccl2*, *Cxcl10*, *Xcl1*, *Slpi.* Type I/II interferon responses: *Isg15*, *Ifi205*, *Ifitm1*, *Ifngr2.* Tissue remodeling & repair: *Thbs1*, *Tgm2*, *Dgat1*, *Plec*. Immune activation & suppression:	Strong inflammatory signature, with high expression of chemokines, cytokines, and tissue remodeling genes, suggesting high activation and

		Cd83, *Socs3*, *Crem*, *Lilr4b*, *Il1r2.* Metabolism & stress adaptation: *Acod1*, *Fabp5*, *Uck2*, *Dusp2/3*	antimicrobial defense, possibly at the expense of immune regulation.
**mLN**	**Mouse Type**	**Key Features**	**Proposed Phenotype**
$CD4^+CD44^-Foxp3^-$ (Tn)	**CNV**	High translation (Rps21, Rps28, Rps29, Rpl35a, Rpl41, Rpl37, Rpl37a, Rpl39), metabolic activity (Atp5e, Atp5mpl, Cox7c, Cox6c, Ndufa2), MHC class II presentation Cd74, H2-Aa).	Highly metabolic naive CD4 cells with strong activation potential
	**ENV**	Stress-resistance (Hsp90ab1, Hspe1, Hspa8, Dnaja1, Ucp2), survival genes (Bcl2, Txnip, Ddit4, Foxn3), adaptive proliferation (Ccnd3, Rsrp1, Pdcd4)	Stress-adapted naive CD4 cells with survival advantage
	**ENV/AUTO**	Strong T cell interactions (Tcf7, Rasgrp1, Rasa3, Grap2, Dock11), cytoskeletal changes (Actn1, Msn, Flna, Apbb1ip, Evl), memory potential (Tspan32, Dgka, Arhgef18)	Memory-like naive CD4 cells with regulatory properties
$CD4^+CD44^+Foxp3^-$ (Tact)	**CNV**	High mitochondrial activity, ribosomal genes, Ccr7, Foxo1	Memory T cells, homeostatic maintenance
	**ENV**	Il7r, Stat1, inflammatory and metabolic genes	Activated T cells, inflammatory response
	**ENV/AUTO**	Top2a, Mki67, chromatin regulators, histone genes	Highly proliferative, effector T cells
$CD4^+Foxp3^+$ (Treg)	**CNV**	Highly expressed genes involved in chromatin remodeling (Hmgb2, H2afv), metabolism (Ndufa3, Cox7b, Atp5mpl), ribosomal activity (Rps28, Rpl35, Rpl37), and T cell signaling (Pik3cd, Prkcq, Grap2).	Highly proliferative and metabolically active Tregs with enhanced survival signaling.
	**ENV**	Enriched in immune response regulators (Stat1, Nfkbia, Pdcd4), inflammatory mediators (Pycard, Txnip, Ifi203), and stress response genes (Hsp90aa1, Junb).	Inflammatory, highly suppressive and stress-responsive Tregs, adapted to environmental challenges.
	**ENV/AUTO**	Express markers such as CTLA-2A, Tnfrsf18, and Gpx4, which suggest	Tregs with a generalized regulatory phenotype, possibly focused on Th2-type responses

		enhanced survival and suppression under inflammatory conditions.	and survival under oxidative stress
$CD8^+CD44^-$ (CD8 naive)	**CNV**	Increased expression of components of the translation machinery and cellular respiration (Rps29, Rps28, Rpl35a, Atp5e). Immune signaling and survival (Jund, Rbm3, Crip1).	Cytotoxic cells with enhanced protein translation, immune signaling, and survival
	**ENV**	Activation or potential transition to an effector or memory state, with expression of genes involved in cell migration, signaling, and survival (Il7r, Ccl5, Hspa8, and Bcl2). Pde3b, Ccl5, and Tmtc2 suggest chemotactic and immune response pathways	Highly cytotoxic/effector cells with enhanced migratory potential
	**ENV/AUTO**	Differentiation or activation towards an effector phenotype (Sp100, Maml2, Stat1, Ctsw), immune cell signaling (Cd37, Ifi203), and cellular adhesion (Flna, Itgb2). Actn1, Myh9, and Nlrc5 suggest cytoskeletal remodeling and immune signaling Strong signaling through immune receptors (Stat1, Pde3b, Filip1l).	Effector T cells expressing factors involved in immune response regulation and stress response
$CD8^+CD44^-$ (CD8 act)	**CNV**	Metabolic and stress-response genes (Hspa5, Hspd1, Ldha, Phb2), cell cycle regulators (Id3, Cdk17)	Homeostatic or metabolically active memory CD8+ T cells
	**ENV**	Immune activation and inflammatory genes (Ccl5, Il2rb, Ifngr1, Stat4, Hopx)	Activated cytotoxic and inflammatory effector CD8+ T cells
	**ENV/AUTO**	Tissue-resident markers and immune regulators (Foxo1, Ctsw, Ms4a4c, Klrd1, Trbv19)	Tissue-resident memory-like CD8+ T cells with enhanced immune surveillance
$CD19^+$ (B cells)	**CNV**	Genes like Hmgb1, Rps28, Rpl36a point to active protein synthesis and cellular differentiation. Rpl41, Vpreb3, Snrpg indicate B cell maturation and immune response. Iglc3, Iglc2 suggest immunoglobulin production. Cox6b1, Apoe, Crip1 imply stress response and immune modulation. Tma7, Sec61g suggest functional	Proliferative, differentiated B cells with stress and immune activation

		adaptations in the endoplasmic reticulum and protein folding.	
	**ENV**	Fgfr2, Klf2, Irf8 suggest immune regulation and signaling pathways. Hspa5, Hspa8, Hsp90aa1 point to stress response. Ighm, Syk, Igkv1-135 suggest immunoglobulin production and signaling. Dock10, Serinc3 imply immune cell migration and lipid metabolism. Jchain, Igkc, Ighg1 indicate class switching and immunoglobulin secretion.	Adapted B cells with metabolic regulation, immune tolerance
	**ENV/AUTO**	Ifi27l2a, Fcer2a, Fcmr point to immune activation and antigen presentation. Ighv2-9-1, Ighv6-3 indicate immunoglobulin variable regions, suggesting BCR diversification. Lcp1, Ezr, Fchsd2 point to immune cell motility and signal transduction. Fgfr2, Zc3h7a suggest cellular survival and differentiation regulation. Igkv10-96, Igkv12-46 suggest BCR diversity and antibody secretion	Memory-like B cells with antigen presentation and inflammatory response
$CD11c^{+}CD11b^{-}$ (DC)	**CNV**	Activation and stress response features, with increased expression of immune-related proteins and ribosomal components, suggesting an active immune state Hmgb2, Cd7, Tmsb10, Rps21, S100a11, Arhgdia).	Activation, Protein Synthesis, Stress Response
	**ENV**	focus on immune signaling and cell cycle regulation, marked by the expression of chemokines, heat shock proteins, and ribosomal proteins, indicating a heightened immune response (Ccl5, Cdk8, Eif5a, S100a6, Hspa5)	Immune Signaling, Cell Cycle, Stress Response
	**ENV/AUTO**	inflammatory and immune-regulatory phenotype with elevated levels of immune activation markers, stress	Immune Activation, Inflammation, Stress Response

		response genes, and proteins involved in cell survival and immune response (Hmgb2, Ccr7, Ctsb, S100a4, Mpeg1)	
$CD11c^{+}CD11b^{+}$	**CNV**	Fcna, C1qa, C1qb, C1qc: Complement system regulation, inflammation. Csf1r: Macrophage colony-stimulating factor receptor, macrophage differentiation. Cx3cr1: Microglial and monocyte migration and activation. Apoe: Lipid metabolism, immune regulation. Selenop, Grn, Fcgr3: Involved in antioxidant defense and innate immunity.	Macrophage-like, inflammation, antigen presentation
	**ENV**	Dscam: Immune cell adhesion and signaling. Stat2, Cd7: Cytokine signaling and immune response regulation. Ctnnd2, Vasp: Cell adhesion, migration, and cytoskeletal organization. Alcam, Ccnd1: Cellular adhesion and proliferation regulation. Mapk14: Stress response and inflammatory pathway activation. S100a9: Inflammatory response, immune cell recruitment.	Dendritic-like, cytokine signaling, adhesion
	**ENV/AUTO**	Hist1h2ae, Tubb5, Tuba1b: Cytoskeletal components, cell division. Xcl1: Chemokine involved in immune cell migration. Irf7, Nfat5: Transcription factors involved in immune response. Naaa, Ppt1: Involved in lipid metabolism and antigen processing. Cdc5l, Mki67: Cell cycle regulation, proliferation markers.	Activated dendritic cells, immune response, cell division
$CD11c^{-}CD11b^{+}$	**CNV**	Involved in immune regulation and inflammation, with markers of antigen processing and presentation (e.g., Hck, Csf1r, and Cebpb). Features suggest cellular response to immune activation (e.g., Nr4a1, Gngt2, Ifi27l2a) and cell survival.	Likely activated antigen-presenting cells (APCs) or macrophage-like cells

	**ENV**	Key involvement in antigen presentation (H2-Ab1, H2-Eb1), immune regulation, and inflammation (e.g., Ccl6, Csf3r, S100a11). The cluster suggests a role in adaptive immunity and inflammation.	Primarily antigen-presenting cells (APCs), likely dendritic cells or macrophages.
	**ENV/AUTO**	Involved in immune responses and antigen presentation (H2-Ab1, C1qb), with markers of inflammation (S100a9, S100a8, Ccl5). Features indicate response to infection and tissue remodeling.	Likely dendritic cells or activated macrophages.

Table.2. Reagents

REAGENT or RESOURCE	SOURCE	IDENTIFIER
**FACS ANTIBODIES**		
Brilliant Violet 650™ anti-mouse CD4 Antibody (clone GK1.5)	BioLegend	Cat# 100469, RRID:AB_2783035
Brilliant Ultra Violet™ 395 CD4 Monoclonal Antibody (clone GK1.5)	eBioscience	Cat# 363-0041-82, RRID:AB_2942113
APC/Fire™ 750 anti-mouse CD4 Antibody (clone GK1.5)	BioLegend	Cat# 100460, RRID:AB_2572111
APC anti-mouse CD4 Antibody (clone GK1.5)	BioLegend	Cat# 100412, RRID:AB_312697
Brilliant Violet 510™ anti-mouse CD4 Antibody (clone GK1.5)	BioLegend	Cat# 100449, RRID:AB_2564587
PerCP/Cyanine5.5 anti-mouse CD8a Antibody (clone 53-6.7)	BioLegend	Cat# 100734, RRID:AB_2075238
BUV661 Rat Anti-Mouse CD8a (clone 53-6.7)	BD	Cat# 569186, RRID:AB_3086874
APC anti-mouse CD25 Antibody (clone PC61)	BioLegend	Cat# 102012, RRID:AB_312861
Brilliant Violet 510™ anti-mouse CD11c Antibody (clone N418)	BioLegend	Cat# 117353, RRID:AB_2686978
APC anti-mouse CD11c Antibody (clone N418)	BioLegend	Cat# 117310, RRID:AB_313779
Brilliant Violet 785™ anti-mouse/human CD11b Antibody (clone M1/70)	BioLegend	Cat# 101243, RRID:AB_2561373
Brilliant Violet 421™ anti-mouse/human CD11b Antibody (clone M1/70)	BioLegend	Cat# 101251, RRID:AB_2562904
PE/Cyanine5 anti-mouse/human CD11b Antibody (clone M1/70)	BioLegend	Cat# 101209, RRID:AB_312792
anti-mouse CD16/32 Antibody (clone 2.4G2)	BioLegend	Cat# 156604, RRID:AB_2783138
APC anti-mouse CD38 Antibody (clone 90)	BioLegend	Cat# 102712, RRID:AB_312933
PE/Cyanine5 anti-mouse/human CD44 Antibody (clone IM7)	BioLegend	Cat# 103010, RRID:AB_312961
Brilliant Violet 711™ anti-mouse/human CD44 Antibody (clone IM7)	BioLegend	Cat# 103057, RRID:AB_2564214

Brilliant Violet 510™ anti-mouse CD45 Antibody (clone 30-F11)	BioLegend	Cat# 103138, RRID:AB_2563061
PE anti-mouse/human CD45R/B220 Antibody (clone RA3-6B2)	BioLegend	Cat# 103208, RRID:AB_312993
PE/Dazzle™ 594 anti-mouse/human CD45R/B220 Antibody (clone RA3-6B2)	BioLegend	Cat# 103258, RRID:AB_2564053
APC/Cyanine7 anti-mouse CD45RB Antibody (clone C363-16A)	BioLegend	Cat# 103310, RRID:AB_528821
PE anti-mouse LPAM-1 (Integrin α4β7) Antibody	BioLegend	Cat# 120605, RRID:AB_493268
PE anti-mouse CD62L Antibody (clone MEL-14)	BioLegend	Cat# 104408, RRID:AB_313095
PE/Dazzle™ 594 anti-mouse CD71 Antibody (clone RI7217)	BioLegend	Cat# 113818, RRID:AB_2749884
PE/Cyanine7 anti-mouse CD73 Antibody (clone TY/11.8)	BioLegend	Cat# 127224, RRID:AB_2716103
PerCP/Cyanine5.5 anti-rat CD90/mouse CD90.1 (Thy1.1) Antibody (clone OX-7)	BioLegend	Cat# 202516, RRID:AB_961437
BUV661 Rat Anti-Mouse CD90.2 (clone 30-H12)	BD	Cat# 741458, RRID:AB_2870927
Brilliant Violet 605™ anti-mouse CD95 (Fas) Antibody (clone SA367H8)	BioLegend	Cat# 152612, RRID:AB_2728202
APC anti-mouse CD117 (c-kit) Antibody (clone S18020A)	BioLegend	Cat# S18020A, RRID: n/a
APC/Fire™ 750 anti-mouse CD185 (CXCR5) Antibody (clone L138D7)	BioLegend	Cat# 145534, RRID:AB_2750495
PE/Cyanine7 anti-human CD199 (CCR9) Antibody (clone L053E8)	BioLegend	Cat# 358910, RRID: n/a
Alexa Fluor 700 CCR10 Antibody (clone 248918)	R&D Systems	Cat# FAB2815N100, RRID: n/a
CD279 (PD-1) Monoclonal Antibody, Super Bright™ 702 (clone J43)	eBioscience	Cat# 67-9985-82, RRID:AB_2722953
PE anti-mouse CD304 (Neuropilin-1) Antibody (clone 3E12)	BioLegend	Cat# 145204, RRID:AB_2561928
PE anti-mouse CD326 (Ep-CAM) Antibody (clone G8.8)	BioLegend	Cat# 118206, RRID:AB_1134172

BUV496 Mouse Anti-Mouse NK-1.1 (clone PK136)	BD	Cat# 741062, RRID:AB_2870674
PE anti-mouse Ly-6A/E (Sca-1) Antibody (clone QA17A36)	BioLegend	Cat# 160906, RRID: n/a
Brilliant Violet 650™ anti-mouse Ly-6C Antibody (clone HK1.4)	BioLegend	Cat# 128049, RRID:AB_2800630
Brilliant Violet 605™ anti-human CD2 Antibody (clone RPA-2.10)	BioLegend	Cat# 300224, RRID:AB_2687243
Alexa Fluor® 700 anti-mouse I-A/I-E Antibody (clone M5/114.15.2)	BioLegend	Cat# 107622, RRID:AB_493727
TCR beta Monoclonal Antibody, Super Bright™ 645 (clone H57-597)	eBioscience	Cat# 64-5961-82, RRID:AB_2723704
PE Rat Anti-Mouse Vα2 TCR (Clone B20.1)	BD	Cat# 553289, RRID:AB_394760
FITC Rat Anti-Mouse Vα 11.1, 11.2 TCR (Clone RR8-1)	BD	Cat# 553222, RRID:AB_394717
FITC anti-mouse TCR Vβ2 Antibody (Clone B20.6)	BioLegend	Cat# 127906, RRID:AB_1227782
FITC Rat Anti-Mouse TCR Vβ7 (Clone TR310)	BD	Cat# 570991, RRID:AB_3686160
Biotin Mouse Anti-Mouse Vβ 8 T-Cell Receptor (Clone F23.1)	BD	Cat# 553860, RRID:AB_395096
FITC Rat Anti-Mouse Vβ 10 TCR (Clone B21.5)	BD	Cat# 553284, RRID:AB_394756
FITC Mouse Anti-Mouse Vβ 12 T-Cell Receptor (Clone MR11-1)	BD	Cat# 553300, RRID:AB_394768
FITC Rat Anti-Mouse Vβ 14 T-Cell Receptor (Clone 14-2)	BD	Cat# 553258, RRID:AB_394738
PE IgA Monoclonal Antibody (clone mA-6E1)	eBioscience	Cat# 12-4204-83, RRID:AB_465918
Brilliant Violet 421™ anti-mouse IgM Antibody (clone RMM-1)	BioLegend	Cat# 406532, RRID:AB_2650930
Brilliant Violet 711™ anti-mouse IgD Antibody (clone 11-26c.2a)	BioLegend	Cat# 405731, RRID:AB_2563342
Brilliant Violet 785™ anti-mouse IFN-γ Antibody (clone XMG1.2)	BioLegend	Cat# 505838, RRID:AB_2629667
PE anti-mouse IL-17A Antibody (clone TC11-18H10.1)	BioLegend	Cat# 506904, RRID:AB_315464
Brilliant Violet 711™ anti-mouse IL-17A Antibody (clone TC11-18H10.1)	BioLegend	Cat# 506941, RRID:AB_2565836
PE anti-mouse IL-17F Antibody (clone	BioLegend	Cat# 517008, RRID:AB_10690818

9D3.1C8)		
APC IL-22 Monoclonal Antibody (clone IL22JOP)	eBioscience	Cat# 17-7222-82, RRID:AB_10597583
TNF alpha Monoclonal Antibody, PE-Cyanine7 (clone MP6-XT22)	eBioscience	Cat# 25-7321-82, RRID:AB_11042728
PerCP-eFluor 710 Phospho-LCK (Tyr505) Monoclonal Antibody (clone SRRCHA)	eBioscience	Cat# 46-9076-42, RRID: n/a
PE-Cyanine7 Phospho-S6 (Ser235, Ser236) Monoclonal Antibody (clone cupk43k)	eBioscience	Cat# 25-9007-42, RRID: n/a
APC Phospho-Stat1 (Tyr701) Monoclonal Antibody (clone Stat1Y701-3E6)	eBioscience	Cat# MA5-37041, RRID: n/a
eFluor 450 Phospho-STAT3 (Tyr705) Monoclonal Antibody (clone LUVNKLA)	eBioscience	Cat# 48-9033-42, RRID: n/a
PE-eFluor 610 Phospho-STAT6 (Tyr641) Monoclonal Antibody (clone CHI2S4N)	eBioscience	Cat# 61-9013-41, RRID: n/a
Alexa Fluor® 647 Phospho-SMAD2 (Ser465/Ser467) (clone E8F3R)	Cell Signaling Technology	Cat# 68550S, RRID: n/a
PE/Dazzle™ 594 anti-T-bet Antibody (clone 4B10)	BioLegend	Cat# 644828, RRID:AB_2565677
BB700 Mouse Anti-GATA3 (clone L50-823)	BD	Cat# 566642, RRID:AB_2813884
PE Mouse anti-Mouse RORγt (clone Q31-378)	BD	Cat# 562607, RRID:AB_11153137
APC FOXP3 Monoclonal Antibody (clone FJK-16s)	eBioscience	Cat# 17-5773-82, RRID:AB_469457
PE-Cyanine7 Arginase 1 Monoclonal Antibody (clone A1exF5)	eBioscience	Cat# 25-3697-82, RRID:AB_2734841
Alexa Fluor™ 700 iNOS Monoclonal Antibody (clone CXNFT)	eBioscience	Cat# 56-5920-82, RRID:AB_2848474
CoraLite® 555 ATP5A1 Monoclonal Antibody (clone 1B10H3)	eBioscience	Cat# CL555-66037, RRID:AB_2919670
CoraLite® Plus 647 CPT1A Polyclonal Antibody	eBioscience	Cat# CL647-15184, RRID:AB_2934912
CoraLite® 594 GLUT1 Monoclonal Antibody (clone 2A5A2)	eBioscience	Cat# CL594-66290, RRID:AB_2934721
CoraLite® Plus 647 G6PD Monoclonal Antibody (clone 2A7B12)	eBioscience	Cat# CL647-66373, RRID:AB_2920281
CoraLite® Plus 647 ACC1 Monoclonal Antibody (clone 1A11G10),	eBioscience	Cat# CL647-67373, RRID:AB_2920292

IDH2 Recombinant Monoclonal Antibody (clone JA55-31)	eBioscience	Cat# MA5-41232, RRID:AB_2898985
PRDX2 Recombinant Monoclonal Antibody (clone JJ090-3)	eBioscience	Cat# MA5-32466, RRID:AB_2809743
HK1 Monoclonal Antibody (clone 3A10)	eBioscience	Cat# MA5-15680, RRID:AB_10979325
Goat anti-Rabbit IgG (H+L) Cross-Adsorbed Secondary Antibody, Alexa Fluor™ 750	eBioscience	Cat# A-21039, RRID:AB_2535710
Goat anti-Mouse IgG (H+L) Highly Cross-Adsorbed Secondary Antibody, Alexa Fluor™ 790	eBioscience	Cat# A11357, RRID:AB_2534140
FITC Streptavidin	BioLegend	Cat# 405201
Brilliant Violet 650™ Streptavidin	BioLegend	Cat# 405231
Brilliant Violet 421™ Streptavidin	BioLegend	Cat# 405225
FITC Streptavidin	BioLegend	Cat# 405201
Purified anti-mouse IFN-γ Antibody (clone XMG1.2)	BioLegend	Cat# 505802, RRID:AB_315396
Purified anti-mouse IL-4 Antibody (clone 11B11)	BioLegend	Cat# 504102, RRID:AB_315316
Purified anti-mouse IL-12 (p70) Antibody (clone C18.2)	BioLegend	Cat# 511802, RRID:AB_2123769
Purified anti-mouse CD3ε Antibody (clone 145-2C11)	BioLegend	Cat# 100302, RRID:AB_312667
Purified anti-mouse CD28 Antibody (clone 37.51)	BioLegend	Cat# 102102, RRID:AB_312867
anti-mouse CD28 superagonist (clone D665)	Bio X Cell	Cat# BE0328, RRID:AB_2819055
**CYTOF ANTIBODIES (custom-made panel-combination of Cat# 201306, Cat# 201310, and on-site tagged CD103, Thy1.1, and hCD2) AND REAGENTS**		
89Y CD45 (clone 30-F11)	Standard Biotools	Cat# 3089005B, RRID:AB_2651152
169Tm TCRβ (clone H57-597)	Standard Biotools	Cat# 3169002B, RRID:AB_2827883
172Yb CD4 (clone RM4-5)	Standard Biotools	Cat# 3172003B, RRID:AB_2811242
153Eu CD8α (clone 53-6.7)	Standard Biotools	Cat# 3153012B, RRID:AB_2885019
175Lu hCD2 (clone RPA-2.10)	Biolegend	Cat# 300202, RRID:AB_314026
151Eu CD25 (clone 3C7)	Standard Biotools	Cat# 3151007B, RRID:AB_2827880
171Yb CD44 (clone IM7)	Standard Biotools	Cat# 3172003B, RRID:AB_2811242
160Gd CD62L (clone MEL-14)	Standard Biotools	Cat# 3160008, RRID:AB_2687840
145Nd CD69 (clone H1.2F3)	Standard Biotools	Cat# 3145005B, RRID:AB_2895115

159Tb PD1 (clone 29F.1A12)	Standard Biotools	Cat# 3159024, RRID:AB_2687839
154Sm CTLA4 (clone UC10-4B9)	Standard Biotools	Cat# 3154008B, RRID:AB_3665152
149Sm CD19 (clone 6D5)	Standard Biotools	Cat# 3149002B, RRID:AB_2814679
209Bi I-A/I-E (clone M5/114.15.2)	Standard Biotools	Cat# 3209006B, RRID:AB_2885025
142Nd CD11c (clone N418)	Standard Biotools	Cat# 3142003B, RRID:AB_2814737
148Nd CD11b (clone M1/70)	Standard Biotools	Cat# 3148003B, RRID:AB_2814738
150Nd Ly6C (clone HK1.4)	Standard Biotools	Cat# 3150010B, RRID:AB_2895118
141Pr Ly6G (clone 1A8)	Standard Biotools	Cat# 3141008B, RRID:AB_2814678
146Nd F4/80 (clone BM8)	Standard Biotools	Cat# 3146008B, RRID:AB_2895117
147Sm CD103 (clone 2E7)	Standard Biotools	Cat# 110902, RRID:AB_2922461
170Er NK1.1 (clone PK136)	Standard Biotools	Cat# 3170002B, RRID:AB_2885023
158Gd Thy1.1 (clone OX-7)	Biolegend	Cat# 202501, RRID:AB_314013
Maxpar® X8 Antibody Labeling Kit, 158Gd—4 Rxn	Standard Biotools	Cat# 201158A
Maxpar® X8 Antibody Labeling Kit, 175Lu—4 Rxn	Standard Biotools	Cat# 201175A
Maxpar® X8 Antibody Labeling Kit, 158Gd—4 Rxn	Standard Biotools	Cat# 201158A
Maxpar® X8 Antibody Labeling Kit, 147Sm—4 Rxn	Standard Biotools	Cat# 201147A
Cell-ID™ Intercalator-Ir (191Ir/193Ir)—500µM	Standard Biotools	Cat# 201192B
Cell-ID™ Cisplatin, 100 µL	Standard Biotools	Cat# 201064
Maxpar® Fix and Perm Buffer—100 mL	Standard Biotools	Cat# 201067
Maxpar® Cell Staining Buffer—500 mL	Standard Biotools	Cat# 201068
EQ Four Element Calibration Beads—100 mL	Standard Biotools	Cat# 201078
**KITS**		
CellTrace™ Violet Cell Proliferation Kit, for flow cytometry	Invitrogen	Cat# C34557

LIVE/DEAD™ Fixable Aqua Dead Cell Stain Kit, for 405 nm excitation	Invitrogen	Cat# L34966
LIVE/DEAD™ Fixable Near IR (876) Viability Kit, for 808 nm excitation	Invitrogen	L34982
CD8a (Ly-2) MicroBeads, mouse	Miltenyi Biotech	Cat# 130-117-044
CD19 MicroBeads, mouse	Miltenyi Biotech	Cat# 130-121-301
EasySep™ Mouse CD4+ T Cell Isolation Kit	Stem Cell Technologies	Cat# 19852
EasySep™ Mouse Naïve CD4+ T Cell Isolation Kit	Stem Cell Technologies	Cat# 19765
IL-10 ELISA kit	Invitrogen	Cat# 88-7105-88
LegendPlex Mouse T Helper Cytokine Panel	BioLegend	Cat# 741044
LPS ELISA kit	LSBio	Cat# LS-F17912
QIAquick PCR & Gel Cleanup Kit	Qiagen	Cat# 28506
RNeasy Mini Kit	Qiagen	Cat# 74106
RNeasy PowerFecal Pro Kit – RNA Extraction from Stool	Qiagen	Cat# 78404
SuperScript™ IV First-Strand Synthesis System	Invitrogen	Cat# 18091200
SAA ELISA kit	Abcam	Cat# ab157723
TNFα ELISA kit	Invitrogen	Cat# BMS607-3
True-Nuclear™ Transcription Factor Buffer Set	BioLegend	Cat# 424401
**ANTIBIOTICS**		
Ceftazidime	Fisher	Cat# 50-488-614
Vancomycin	Fisher	Cat# 50-213-727
Amphotericin-B	Fisher	Cat# 50-213-137
Fluconazole	Fisher	Cat# F06775G
5-Fluorocytosine	Fisher	Cat# F032125G
**PRIMERS (5'→3')**		
806RB Reverse PCR Primer	IDT	CAA GCA GAA GAC GGC ATA CGA GAT AGT CAG CCA GCC GGA CTA CNV GGG TWT CTA AT
16S Read 1 Sequencing Primer	IDT	TAT GGT AAT TGT GTG YCA GCM GCC GCG GTA A
16S Read 2 Sequencing Primer	IDT	AGT CAG CCA GCC GGA CTA CNV GGG TWT CTA AT
16S Index Sequence Primer	IDT	AAT GAT ACG GCG ACC ACC GAG ATC TAC ACG CT
ITS1-F Forward Primer	IDT	AAT GAT ACG GCG ACC ACC GAG ATC TAC ACG GCT TGG TCA TTT AGA GGA AGT AA

ITS Read 1 Sequencing Primer	IDT	TTG GTC ATT TAG AGG AAG TAA AAG TCG TAA CAA GGT TTC C
ITS Read 2 Sequencing Primer	IDT	CGT TCT TCA TCG ATG CVA GAR CCA AGA GAT C
ITS Index Sequence Primer	IDT	TCT CGC ATC GAT GAA GAA CGC AGC CG
Barcoded primers (normalized to 8 nmole)	IDT	variable
CalbicansF (C. albicans)	IDT	TTTATCAACTTGTCACACCAGA
CalbicansR (C. albicans)	IDT	GGTCAAAGTTTGAAGATATACGT
CdubliniensisF (C. dubliniensis)	IDT	TTGGGTTTGCTTGAAAGATGAT
CdubliniensisR (C. dubliniensis)	IDT	AAAGTTTGAAGAATAAATGGC
PO3F (P. omnivora)	IDT	CGAGCGTCAGCATAACAAAA
PO2R (P. omnivora)	IDT	CACCACCATACTACGTCAAAGG
EF1-Fusa-spp-Bioger-F (Fusarium)	IDT	TCGTCGTCATCGGCC
EF1-Fusa-spp-Bioger-R (Fusarium)	IDT	AGTGATCATGTTCTTGATGAAATC
BtubfusaterF (Microdochium)	IDT	CACGGTCTCGACAGCAATG
BtubfusaTerR (Microdochium)	IDT	ATGGTACCGGGCTCGAGAT
Aversi_ITS_ f (Aspergillus)	IDT	CTGAGAGTGATGCAGTCTGAGTCTG
Aversi_ITS_ r (Aspergillus)	IDT	AGTTCGCTGCGTTCTTCATC
ITS4bBasi (Basidiomycota)	IDT	CAG GAG ACT TGT ACA CGG TCC AG
5.8srBasi (Basidiomycota)	IDT	TCG ATG AAG AAC GCA GCG
ITS1fUniv (panfungal)	IDT	TCC GTA GGT GAA CCT GCG G
5.8sUniv (panfungal)	IDT	CGC TGC GTT CTT CAT CG
Eubacterium rectale/Clostridium coccoides (Clos XIV)_UniF338	IDT	ACTCCTACGGGAGGCAGC
Eubacterium rectale/Clostridium coccoides (Clos XIV)_C.cocR491	IDT	GCTTCTTAGTCAGGTACCGTCAT
Lactobacillus/Lactococcus_LabF362	IDT	AGCAGTAGGGAATCTTCCA
Lactobacillus/Lactococcus_LabR677	IDT	CACCGCTACACATGGAG
Bacteroides_BactF285	IDT	GGTTCTGAGAGGAGGTCCC
Bacteroides_UniR338	IDT	GCTGCCTCCCGTAGGAGT
Segmented filamentous bacteria_SFB736F	IDT	GACGCTGAGGCATGAGAGCAT
Segmented filamentous bacteria_SFB844R	IDT	GACGGCACGGATTGTTATTCA
Enterobacteriaceae_Uni515F	IDT	GTGCCAGCMGCCGCGGTAA
Enterobacteriaceae_Ent826R	IDT	GCCTCAAGGGCACAACCTCCAAG
A. muciniphila_AM1	IDT	CAGCACGTGA AGGTGGGGAC
A. muciniphila_AM2	IDT	CCTTGCGGTTG GCTTCAGAT
Ruminococcus_f	IDT	
Ruminococcus_R	IDT	
universal bacteria_ UniF340	IDT	ACTCCTACGGG AGGCAGCAGT
UniR514_ UniF340	IDT	ATTACCGCGGC TGCTGGC
Adcy4_5’	IDT	GCTGACCATCCTCCTCTTGA

Adcy4_3’	IDT	CCTGATGATGGTGATGCTGA
Adcy7_5’	IDT	GTGGAGAGCGAGAACATCCA
Adcy7_3’	IDT	TGGGTCTTGGCTTTCTCTGA
Pde3b_F	IDT	GAGAGGCCCTTCATGGAGAC
Pde3b_R	IDT	TGGTGAGGTCCTCTGGTCTT
Cx43_F	IDT	CAGACAGGTCTGAGAGCCCGAACTCT
Cx43_R	IDT	AAGGACCCAGAAGCGCACGTGAGAG
Ins1_f	IDT	CCTGTTGGTGCACTTCCTAC
Ins1_r	IDT	TGCAGTAGTTCTCCAGCTGG
Gapdh_f	IDT	CAAATGGTGGAAGCACAGTTGGCA
Gapdh_r	IDT	TTGTGTCCAGGTCCTCCATGATGT
**REAGENTS, BUFFERS, etc.**		
2-mercaptoethanol	Gibco	Cat# 21-985-023
Alexa Fluor 594 LPS (E. coli serotype 055:B5)	Invitrogen	Cat# L23353
ABH hydrochloride	Sigma	Cat# SML1466
ACK Lysing Buffer	Gibco	Cat# A4000225501
Agarose	Fisher	Cat# BP1356-500
Bovine Serum Albumin solution	Sigma	Cat# A1595-50ML
Brefeldin A	BioLegend	Cat# 420601
Collagenase D	Roche	Cat# 11088882001
DAPI	Invitrogen	Cat# D3571
DNaseI	Roche	Cat# 10104159001
DMSO	Corning	Cat# 25-950-CQC
Dynabeads™ Mouse T-Activator CD3/CD28 for T-Cell Expansion and Activation	Invitrogen	Cat# 11456D
EDTA, 0.5M, pH 8.0	Invitrogen	Cat# 15-575-020
Essential Amino Acids Solution (50X)	Gibco	Cat# 11130051
Ethidium bromide	Invitrogen	Cat# 15-585-011
FBS	HyClone	Cat# SH30396.03
FTY720	Sigma	Cat# SML0700
Glucose	Sigma	Cat# 49139-250G
HBSS	Corning	Cat# 55-022-PB
HEPES (1M)	Cellgro	Cat# MT25060CI
Ionomycin	Sigma	Cat# 10634-1MG
iTaq Universal SYBR Green Supermix	BioRad	Cat# 1725122
LPS (ultrapure; E. coli O55:B5)	InvivoGen	Cat# tlrl-pb5lps
Monensin	BioLegend	Cat# 420701
Non-Essential Amino Acids Solution (100X)	Gibco	Cat# 11-140-076

Normal rat serum	Stem Cell Technologies	Cat# 13551
PBS	Cellgro	Cat# 55-031-PC
PCR-grade water	Sigma	Cat# W3500
Pen/Strep	Gibco	Cat# 15-140-122
Platinum Hot Start PCR Master Mix	Fisher	Cat# 13000014
PMA	Sigma	Cat# P8139-5MG
RPMI 1640	Cellgro	Cat# MT10040CVMP
Sodium Pyruvate (100 mM)	Gibco	Cat# 11-360-070
SsoAdvanced Universal SYBR Green Supermix	BioRad	Cat# 1725272
SYTO-BC	Invitrogen	Cat# S34855
Streptozotocin	Sigma	Cat# S0130-100MG
True-Nuclear™ Transcription Factor Buffer Set	BioLegend	Cat# 424401
**10x GENOMICS scRNAseq REAGENTS**		
Chromium Next GEM Single Cell 5' Kit v2, 16 rxns	10X Genomics	Cat# 1000263
Chromium Next GEM Single Cell 5' Kit v2, 4 rxns	10X Genomics	Cat# 1000265
Chromium Next GEM Chip K Single Cell Kit, 48 rxns	10X Genomics	Cat# 1000286
Chromium Next GEM Chip K Single Cell Kit, 16 rxns	10X Genomics	Cat# 1000287
Library Construction Kit, 16 rxns	10X Genomics	Cat# 1000190
Dual Index Kit TT Set A 96 rxns	10X Genomics	Cat# 1000215
Chromium Single Cell Mouse TCR Amplification Kit, 16 rxns	10X Genomics	Cat# 1000254
Chromium Single Cell Mouse BCR Amplification Kit, 16 rxns	10X Genomics	Cat# 1000255
**BACTERIAL STRAINS**		
A. muciniphila	ATTC	Cat# BAA-835
