# Supplementary material for: Soil-derived microbiota induces T regulatory cells and protect against mouse colitis, metabolic disease, and sepsis
Source: Gut Microbes. 2026 May 24;18(1):2675089. doi: 10.1080/19490976.2026.2675089 (PMC13203056; doi:10.1080/19490976.2026.2675089)
Supplement: Supplementary Material — Supplementary_figure_legends.docx [file KGMI_A_2675089_SM3082.docx]

**Supplementary figure legends:**

**Fig. S1. Housing of mice in dirt as a novel model of natural environmental exposure**. (A) Study design. Sex- and age-matched inbred laboratory mice housed in conventional corncob bedding (CNV) were moved to cages with dirt as bedding (ENV) and exposed to dirt containing microbiota. To assess the effects of live microorganisms in the dirt, a cohort of mice was housed in cages with autoclaved dirt obtained from the same location (ENV/AUTO). (B) Expression of lineage-tracking reporters (Nur77^GFP^, Foxp3^hCD2^, and IL10^Thy1.1^) by lymphocytes in ENV mice. Flow cytometry analysis of cLP is shown in gated live TCRβ^-^B220^-^ innate, and CD4^+^TCRβ^+^ cells. (C) Environmental mouse facility. In-ground and in-room systems are shown. Data presented in the manuscript was made using in-room system. (D, E) 16S rRNA fecal microbiota analysis is depicted as PCoA (D) and taxa (class level) bar graphs (E) (n=10 CNV, n=10 ENV, n=4 ENV/AUTO; feces from two mice per cage; animals from different cages were not littermates). Bray-Curtis dissimilarity calculated from class‑level relative abundance profile. (F) Comparison of ENV adaptive and innate cell phenotype of GSU and Jax mice. (G) Frequencies of blood CD4^+^ and CD11b^+^ cells were obtained with FACS at the indicated time points. (H) Dirt microbiota comparison. Taxa plots at the phylum level, displaying the top 20 species. The Georgia (GA) samples show a higher abundance of *Verrucomicrobia* and *Ascomycota* compared to samples from other states in the US. GA_1: ENV facility, GA_2: ~12 miles from GA_1, GA_3: ~50 miles from GA_1, GA_4: ~150 miles from GA_1. **p<0.01, ****p<0.0001 Student t-test.

**Fig. S2. scRNAseq reveals differences between laboratory and environmental mice.** (A) The heatmap represents clustering and differentially expressed genes in mLN CD45^+^ cells (see Fig. 3). (B, C) Clusters identified by Loupe (10x Genomics; Fig. 3) are shown. (D) Representative gene expression patterns for CD4 are shown on t-SNE plots for the indicated mice. The scale bars represent expression levels. (D) Representative gene expression patterns for CD4 is shown on t-SNE plots for the indicated mice. The scale bars represent expression levels. (E) The heatmap represents differentially expressed genes in cLP and mLN CD45^+^ cells (see Fig. 2 and 3). (F) mLN innate cell analysis. Mean values and SD are shown. (G) Gene Ontology (GO) terms for the indicated mouse based on their gene expression. (H) Network‑level pathway analysis of mLN transcriptomes generated with Metscape. *p<0.05, ***p<0.001, ****p<0.0001 one-way ANOVA followed by Tukey’s post-test.

**Fig. S3. Activation of ENV B cells enhances IgA coating of fecal microbiota**. (A) Typical FACS analysis of mLN germinal center (GC) B cell frequencies and IgA expression by naïve (IgD^high^) and activated/memory (IgD^low^) B cells. (B, C) Quantification of the GC and IgDlowIgA+ cells. (D, E) Elevated frequencies of ENV Tfh cells. Representative FACS (D) and summary (E) is shown. (F, G) Quantification of serum IgA and IgG in naïve CNV and ENV mice. (H, I) IgA coating is elevated in ENV mice. Typical FACS plot (H) and summary (I) are shown. Feces from Rag1^k/o^ mouse was used as a negative control. Microbiota were pre-gated using SytoBC. (J) 16S rRNA analysis of FACS-sorted IgA^+^ fecal microbiota (n=6 mice/group). (K) PCoA analysis of data presented in (J). Bray-Curtis dissimilarity calculated from class‑level relative abundance profile. Each symbol represents a single mouse or fecal sample from a single mouse. ****p<0.0001 assessed using Student t-test.

**Fig. S4. Housing mice in dirt activates T cells and enhances anti-inflammatory/tolerogenic phenotype of CD4^+^ cells.** Frequencies of activated (CD44^+^) mLN CD4^+^ (A) and CD8^+^ (B) T cells. (C, D) Proximal (pLck) and distal (Nur77^GFP^) TCR signaling is enhanced in ENV mice. Data from mLN cells is shown. (E, F) IL-10^+^ Tr1 (E) and Treg (F) cells are expanded in ENV mice. (G) Environmental mice have elevated population of anergic (CD44^+^FR4^+^ CD73^+^) CD4^+^ cells. (H) Proliferation of mLN ENV adaptive but not innate cells is perturbed. Mean values and SD are shown. *p<0.05, **p<0.01, ****p<0.0001 one-way ANOVA followed by Tukey’s post-test.

**Fig. S5. ENV Tregs and MDSCs show enhanced suppressive capabilities.** (A) ENV adaptive but not innate cells have lower metabolism measured by mTOR activity (phospho-S6; data for CD4^+^ and CD11b^+^ is shown). (B) Expression of connexin 43 (Cx43), adenylate cyclases (Adcy 4 and 7) and phosphodiesterase 3b (Pde3b) was measured in FACS-sorted Tregs from CNV and ENV mice by qPCR. (C) *In vitro* Treg suppression assay. FACS-sorted responder cells (Tn) and Tregs were loaded with CTV (cell trace violet), mixed at the indicated ratio and activated with aCD3/aCD28 beads. Dilution of the CTV was measured at day 4. (D) The role of IL-10 and Cx43 in ENV Treg-mediated suppression. Anti-IL10 antibody and GAP26 peptide disrupting Cx43 gap junctions were used. (E) CD28SA (D665 Ab) does not increase Treg numbers in ENV animals. Mice were injected with the Ab and mLN Tregs were analyzed four days later. (F) MDSC inhibition assay. MDSC were mixed with Tn cells at the indicated ratios. Tn cells were activated as in (C). (G) Expression of Arg-1 in CD11b^+^ cells is shown. (H) Blocking IL-10 and Arg-1 reduces MDSC inhibitory function. (I) Expression of inducible nitric oxide synthase (iNOS), reactive oxygen species (ROS) and superoxide was measured by FACS in CD11b^+^ cells. (J) Total mitochondrial content does not differ between CNV and ENV innate cells. Each symbol indicates an individual animal. Mean values and SD are shown. *p<0.05, **p<0.01, ***p<0.001, ****p<0.0001 one-way ANOVA followed by Tukey’s post-test.

**Fig. S6**. **Gut-homing and not disturbance in their precursors or antigenic cell receptor usage is responsible for lower number of peripheral T cells**. (A) Frequencies of thymic CD4/CD8 subpopulations. (B) Frequencies of thymic MHCII^+^ epithelial and DCs. (C) Frequencies of thymic CD4SPCD24^lo^ Tregs. (D) Frequencies of bone marrow stem cells (Sca1), hematopoietic precursors (c-Kit), pre/pro-B cells (B220/CD38), and innate precursors (CD11b/Ly6C). Each symbol depicts a separate mouse. Analysis was done using cells isolated from 16-week-old CNV and ENV animals by FACS. SP: CD4^+^CD8^-^ or CD4^-^CD8^+^, DP: CD4^+^CD8^+^, DN: CD4^-^CD8^-^. (E, F) 16-week-old CNV and ENV mice (n=6/group) were injected with FTY720. Peripheral blood was collected at indicated times and analyzed by FACS. (E) Frequencies of CD4^+^ cells and (F) CD4^+^ cells expressing CCR9, CCR10 or α4β7 are plotted. **p<0.05, **p<0.01, ***p<0.001, ****p<0.0001 one-way ANOVA followed by Tukey’s post-test.

**Fig. S7**. **Analysis of metabolic pathways in immune cells reveals changes induced by environmental microbiota.** FACS analysis of enzymes (A; scheme) involved in glycolysis (B), oxidative stress, bioenergetics (TCA cycle, electron transport chain; C, D) and fatty acid oxidation (E, F), was measured by FACS in gated naïve (Tn) and activated (act) CD4 and CD8 cells, and CD11b myeloid mLN cells. Each symbol indicates a separate mouse. Mean and SD are shown. *p<0.05, **p<0.01, ***p<0.001, ****p<0.0001 one-way ANOVA followed by Tukey’s post-test.

**Fig. S8. Chronic colitis is reduced in environmental mice.** (A) Typical pictures of colons isolated from indicated CNV or ENV TCRα^k/o^ mice transferred with FACS-purified naïve CNV or ENV CD4^+^ cells from CNV or ENV mice. (B) Summary of colon measurements of mice from (A) (n=15-17/group). (C) Representative H&E stainings and summary of colitis scores (D). (E) Typical Treg and cytokine FACS data. Expression of Foxp3^hCD2+^ cells and indicated cytokines is shown in gated live/CD4^+^TCRβ^+^ cells. (F, G) Tfh analysis in mice described above. (H, I) Serum immunoglobulin (IgA, IgG) assessment. Animals were analyzed 8 weeks post transfer. Each symbol in (B, D, G-I) indicates an individual animal. Mean values and SD are shown. *p<0.05, **p<0.01, ***p<0.001, ****p<0.0001 one-way ANOVA followed by Tukey’s post-test.

**Fig. S9**. **Gram-negative bacteria are responsible for ENV phenotype**. CNV mice were exposed to dirt in ENV facility for 8 weeks. Mice were fed with antibiotics targeting Gram-negative, Gram-positive bacteria or fungi and analyzed by FACS at week 8. (A) Frequencies of mLN T cells and CD11b^+^ cells are shown on graphs. (B) ENV phenotype is transferable by fecal microbiota transplantation (FMT). Indicated mice received indicated FMT by oral gavage (3 times/week for 2 weeks, followed by 1x/week for weeks 3-5). FACS analysis was done at week 8. Frequencies of mLN T and CD11b^+^ cells are shown on graph. Each symbol indicates a separate mouse. (C) FMT recapitulates dirt exposure. Metagenomic analysis of bacteria at the genus level of the indicated FMT groups. Data from representative mouse of n=2/group is shown. (D, E) *A. muciniphila* partially restores ENV immune cell phenotype ((D) mLN Treg frequencies are shown) and function ((E) chronic colitis model). ΔG(-): ceftazidime depleting Gram-negative bacteria, ΔG(+): vancomycin targeting Gram-positive bacteria, Δfungi: fluconazole/amphotericin B/5-Fluorocytosine depleting fungi. Akk-A. muciniphila. *p<0.05, **p<0.01, ***p<0.001, ****p<0.0001 one-way ANOVA followed by Tukey’s post-test.

**Fig. S10**. **ENV immunity protects host from sepsis by sparing IL10^+^CD4^+^ cells**. (A-G) 16-week-old CNV and ENV mice were injected with a lethal dose (10 mg/kg) of LPS, and mice were analyzed by FACS at t=48 hrs. Frequencies of mLN CD4^+^ (A), activated CD4^+^ cells, (B) Tr1 cells (C), Tregs (D), and innate CD11b^+^ cells (E) expressing IL-10 (F) are shown on the graphs. (G, H) Clearance of LPS. CNV and ENV CD11b^+^ innate cells were exposed *in vitro* to LPS labelled with Alexa Fluor 594 (AF594; 100 ng/ml). Twelve hours later LPS levels in culture supernatant were analyzed by ELISA (G) and by FACS (H). Each symbol represents a separate animal. (I) scRNAseq analysis of TLR4 expression on CD45^+^ mLN cells in CNV and ENV mice. Expression levels are based on the color intensity and depicted on the violin plots. inactiv-cells incubated in 10% sodium azide (NaN_3_) for 15 min followed by 45 min in 1% NaN_3_ in culture media before adding LPS. *p<0.05, **p<0.01, ***p<0.001, ****p<0.0001 one-way ANOVA followed by Tukey’s post-test.

**Fig. S11**. **Gram-negative bacteria set up protection form sepsis**. (A) Overview of the experiment. Some mice were depleted with Gram-negative bacteria after which were colonized with feces from mice depleted with Gram(+) bacteria, or with *Akkermansia muciniphila*, or received low-dose (50 μg/kg) LPS prior to experiment; all control mice received PBS at the indicated times. 16-week-old CNV and ENV mice were injected lethal dose (10 mg/kg) of LPS. (B) Survival is shown for all groups. (C, D) Analysis of serum cytokine (IL-10, TNFα), expression and animal temperature (E) at indicated time points is shown. (F) Frequencies of mLN CD4^+^, Tregs, and Tr1 cells are shown. Experiment was repeated twice with a total of n=6 mice/group. ΔG(-): ceftazidime depleting Gram-negative bacteria, ΔG(+): vancomycin depleting Gram-positive bacteria A.m: *A. muciniphila*, FMT: fecal microbiota transplantation. Data from two separate experiments is shown with a total of n=six mice. *p<0.05, **p<0.01, ***p<0.001 one-way ANOVA followed by Tukey’s post-test.
