# Supplementary figures and images for: Soil-derived microbiota induces T regulatory cells and protect against mouse colitis, metabolic disease, and sepsis
Source: Gut Microbes. 2026 May 24;18(1):2675089. doi: 10.1080/19490976.2026.2675089 (PMC13203056; doi:10.1080/19490976.2026.2675089)

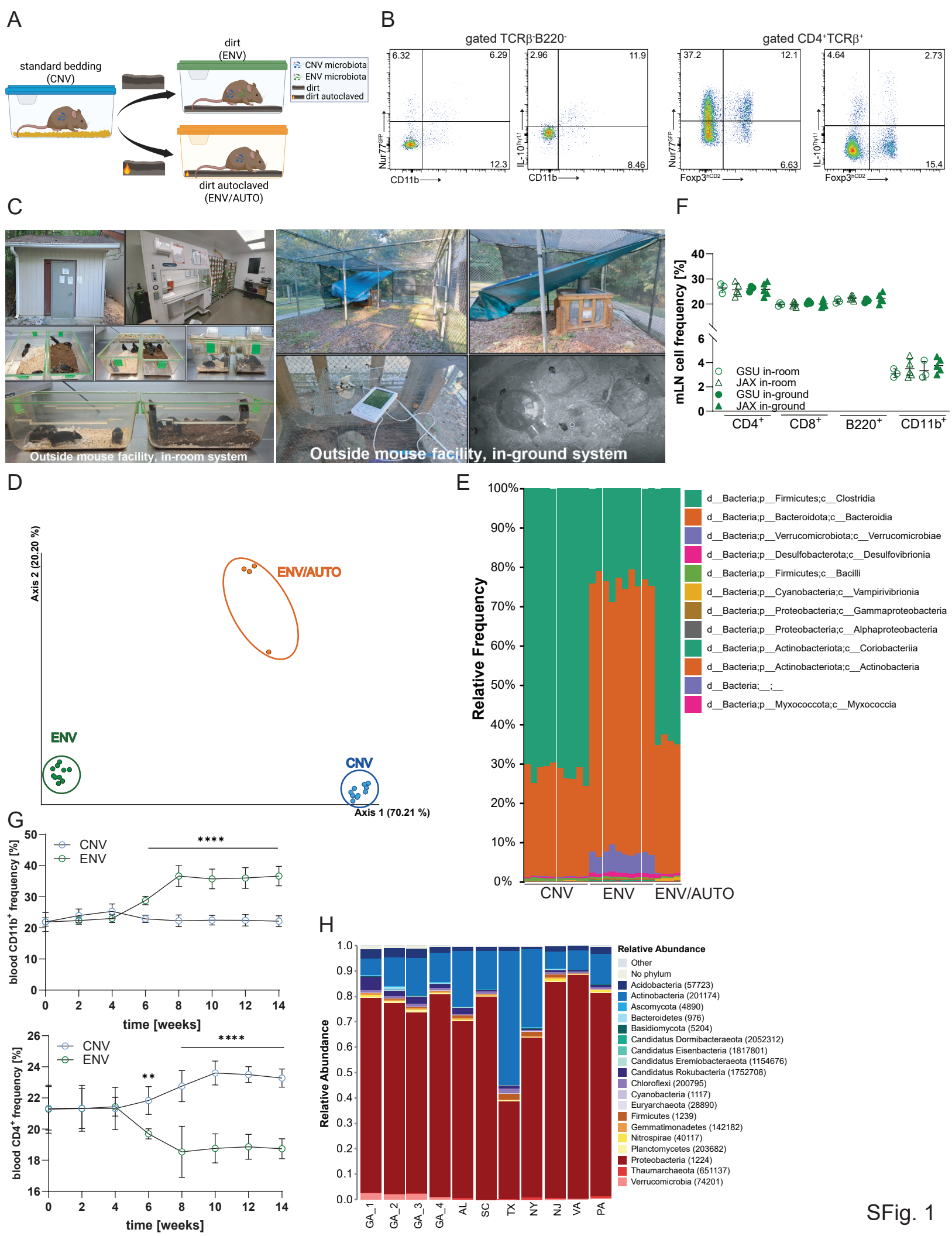

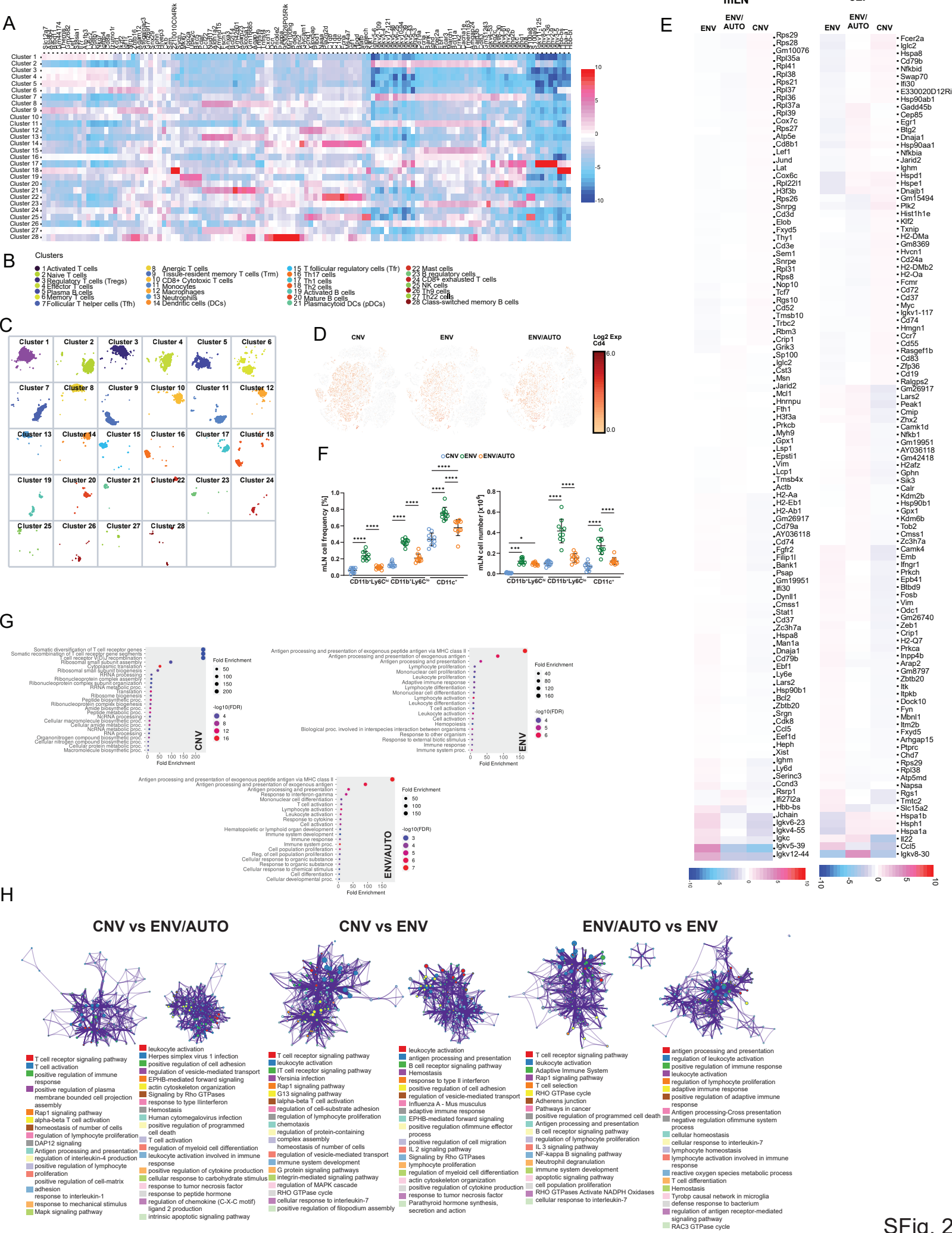

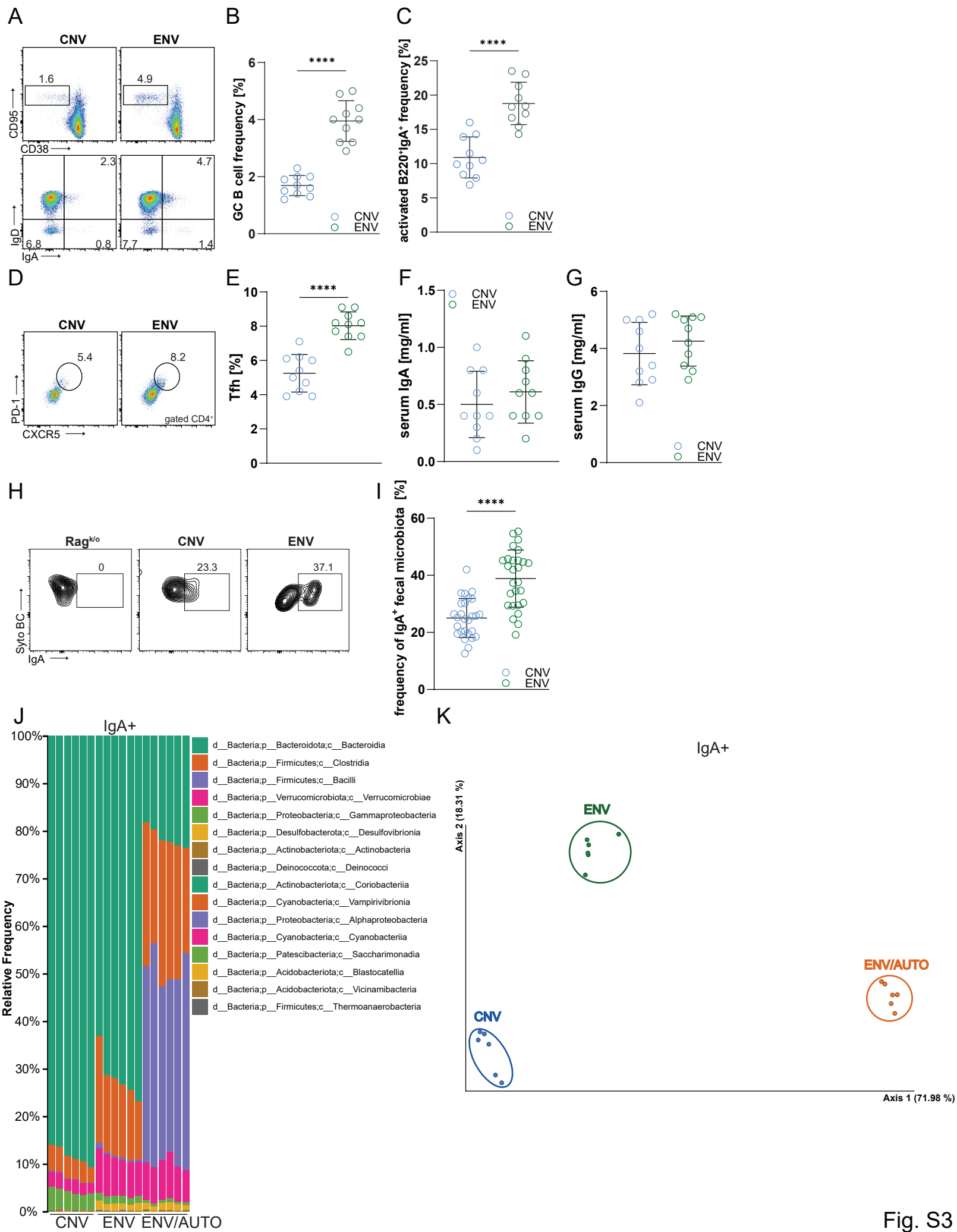

Fig. S3

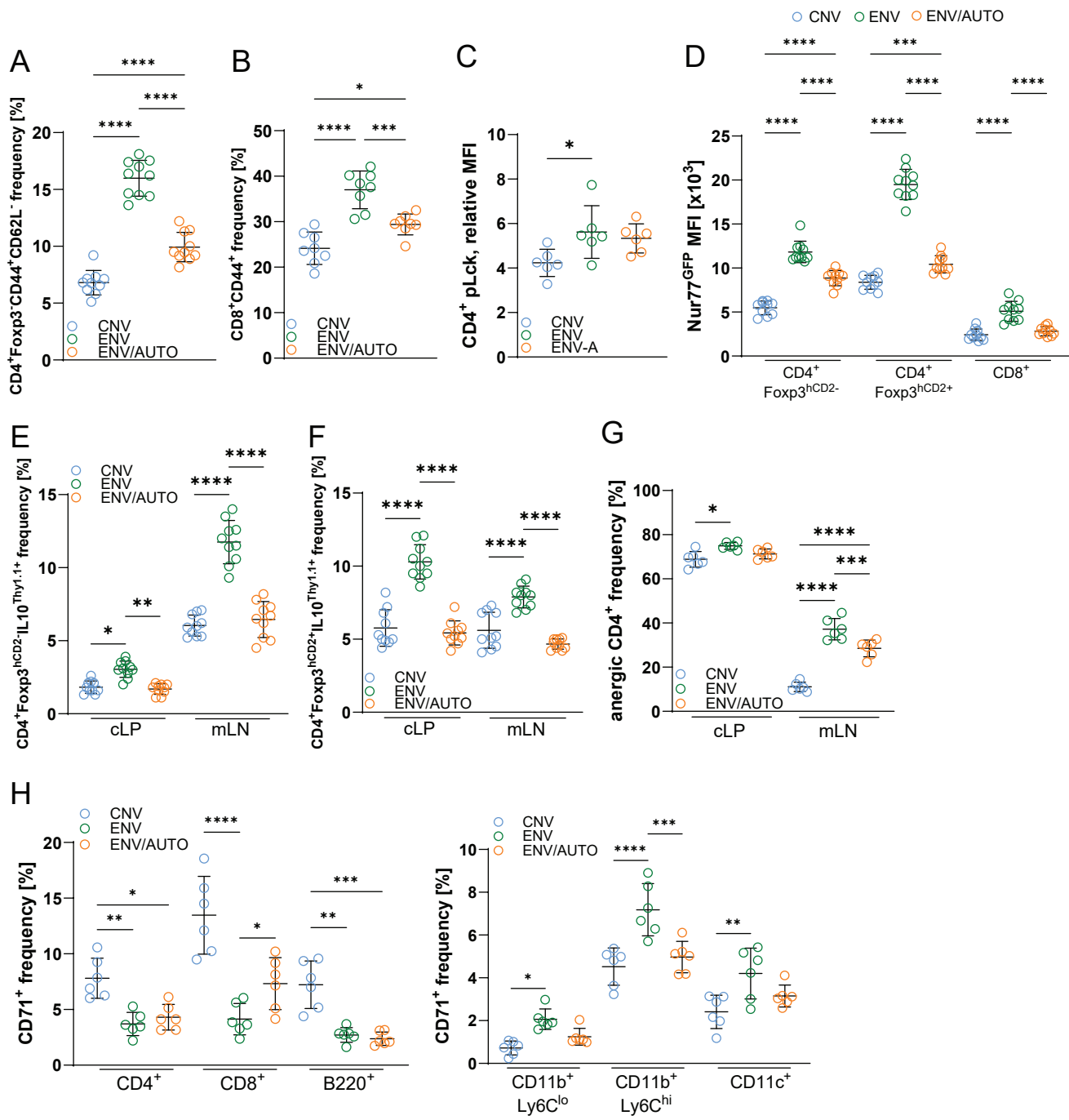

SFig. 4

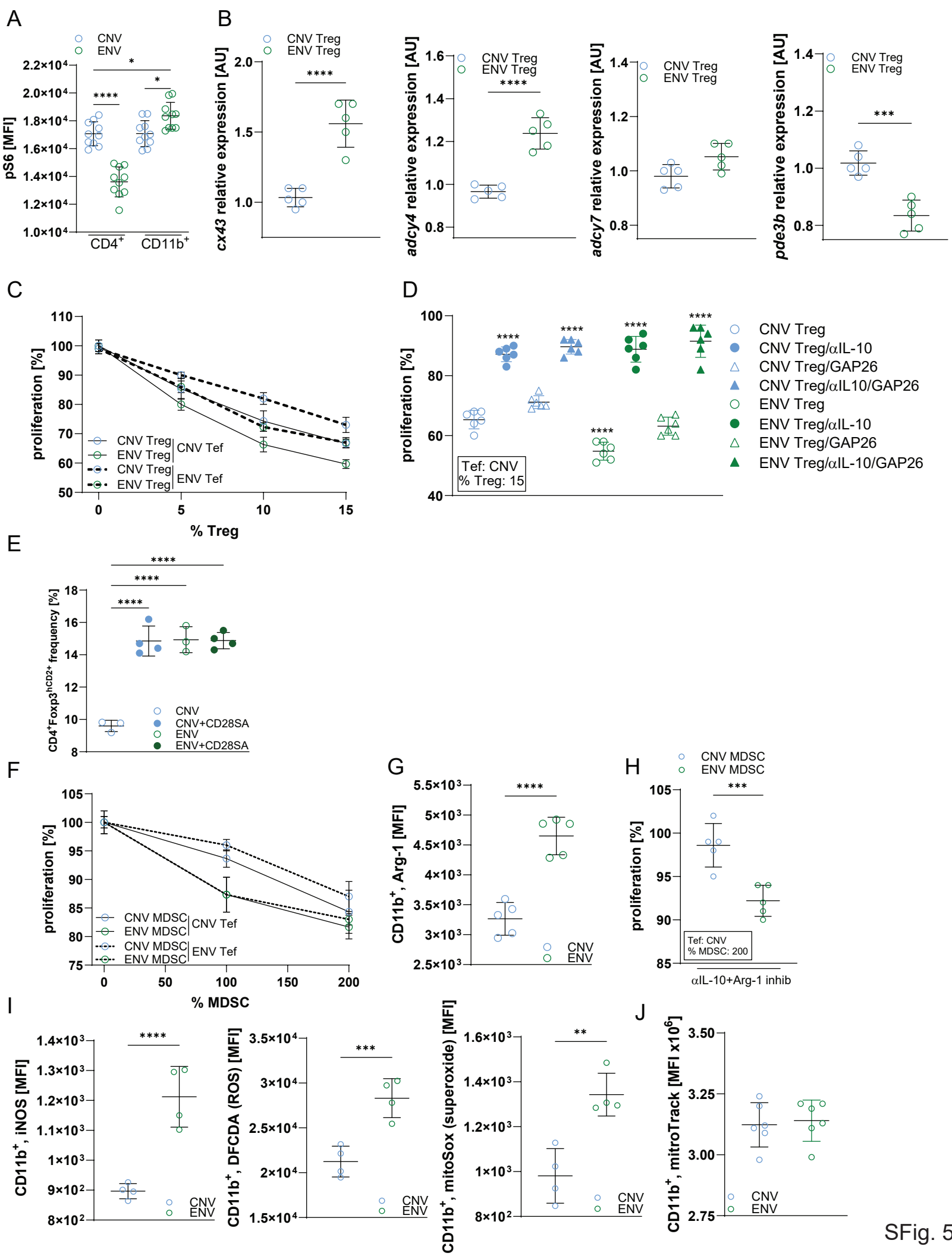

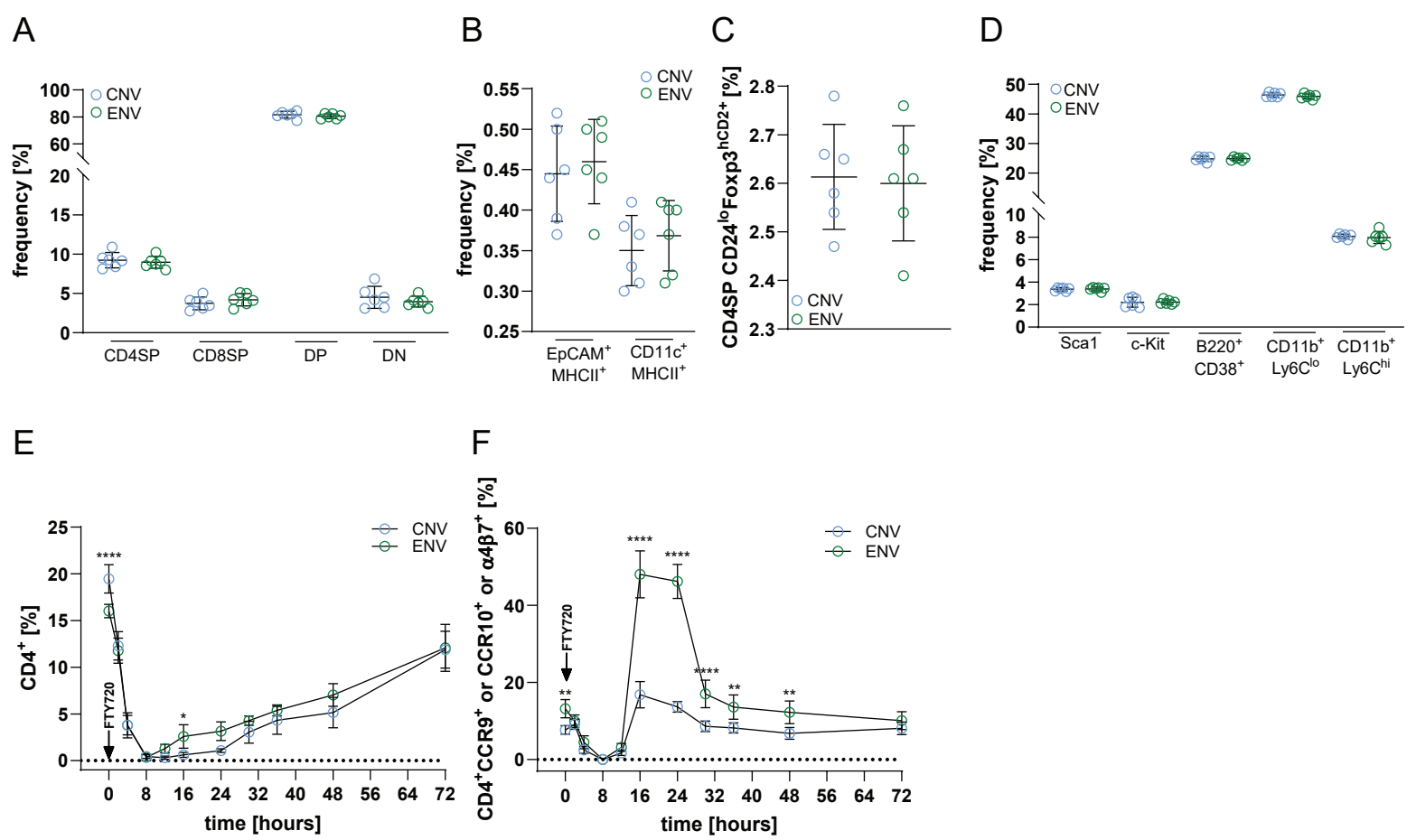

SFig. 6

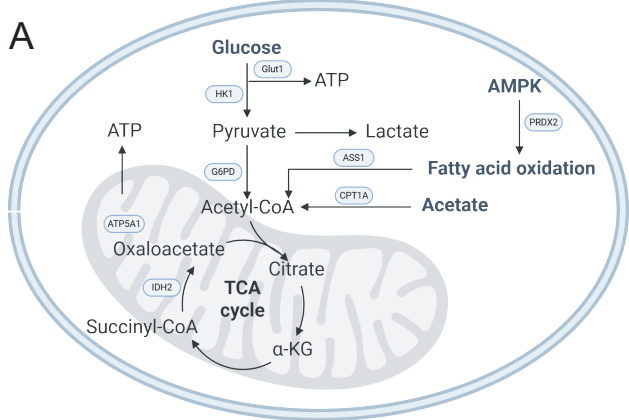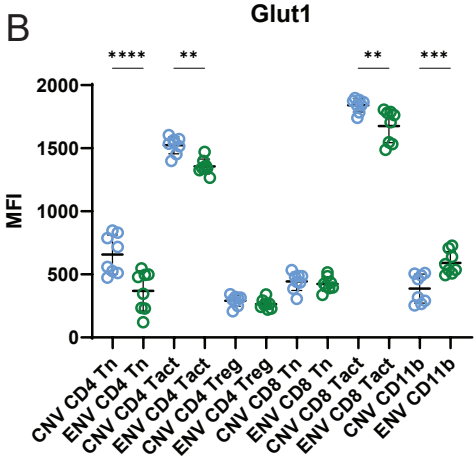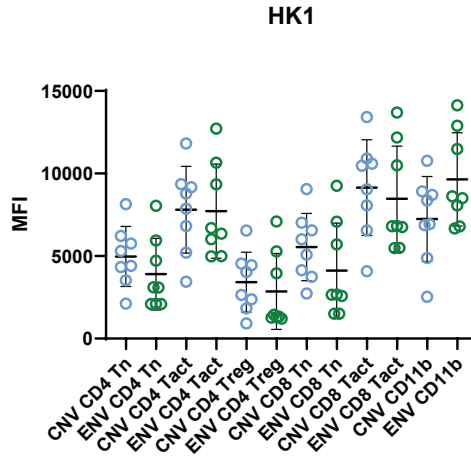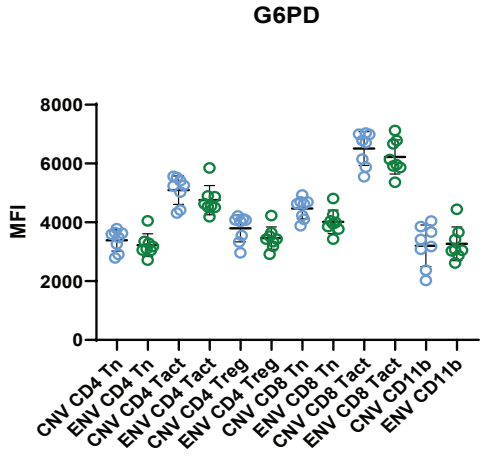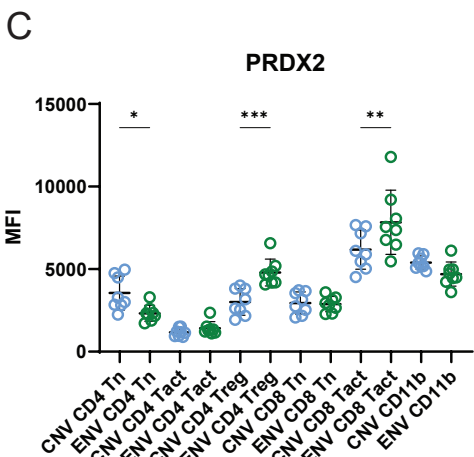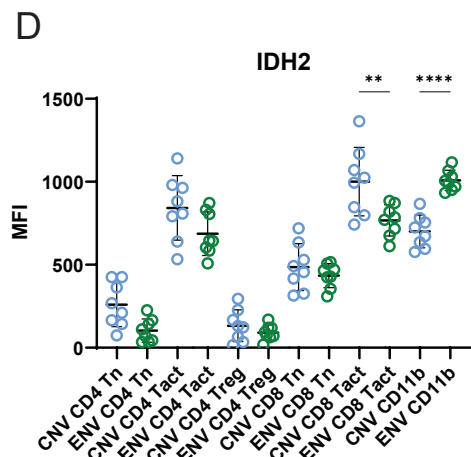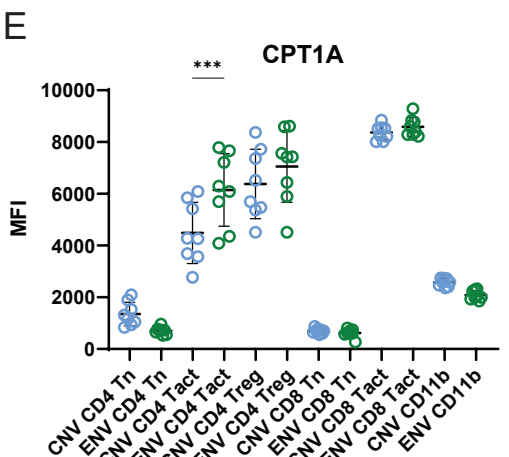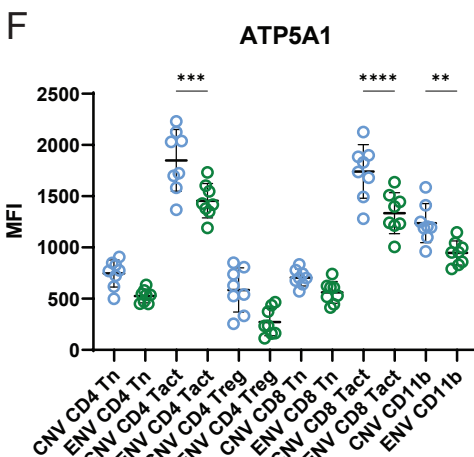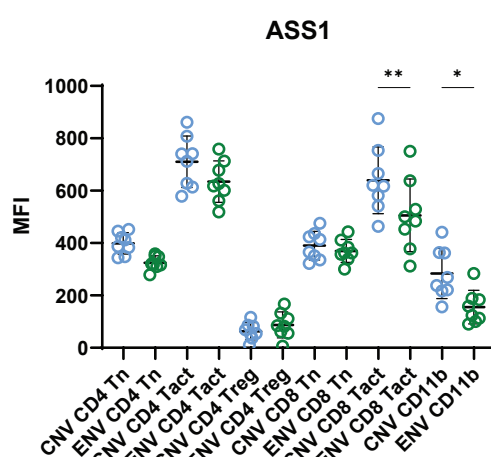

B

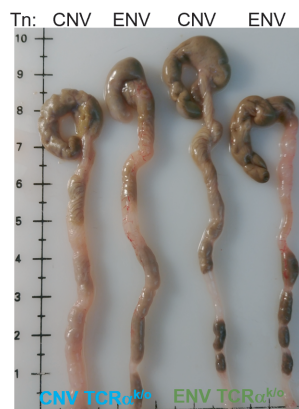

C

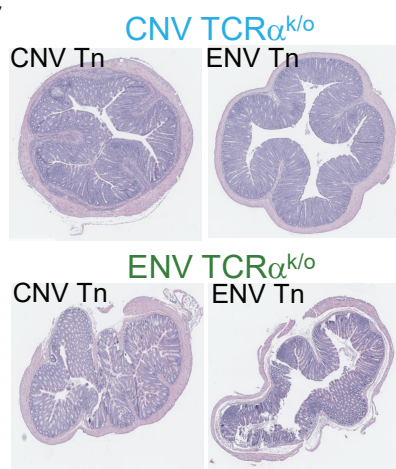

B

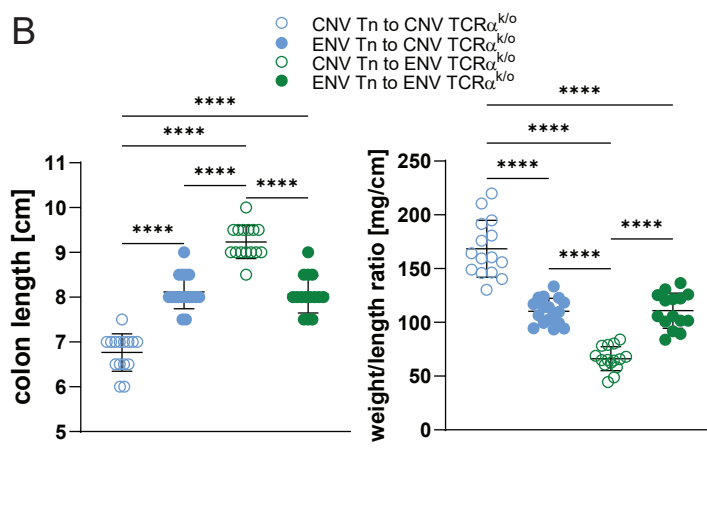

D

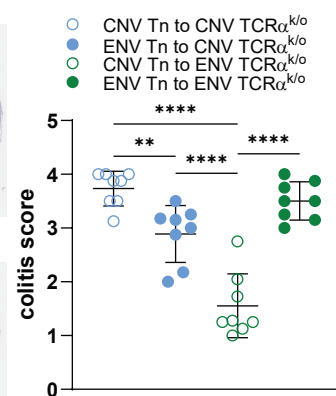

E

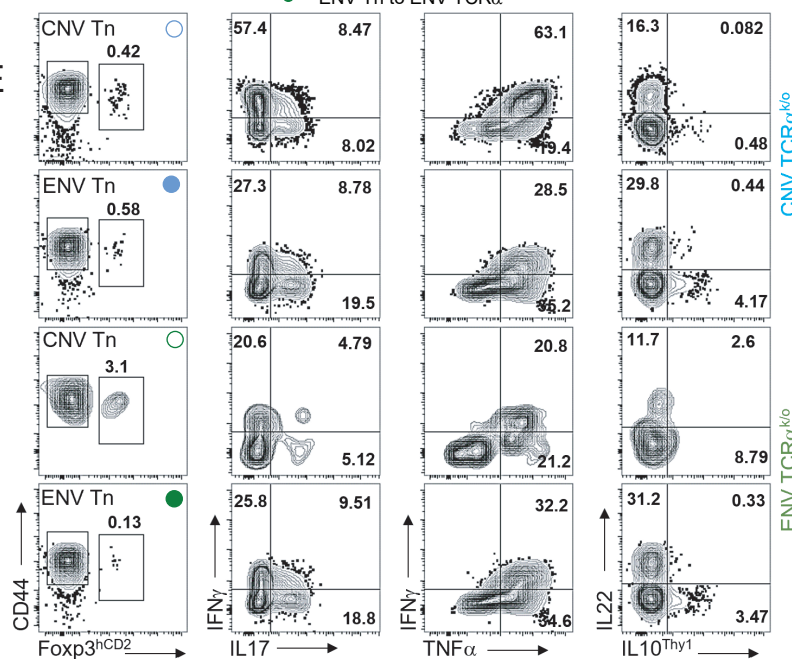

F

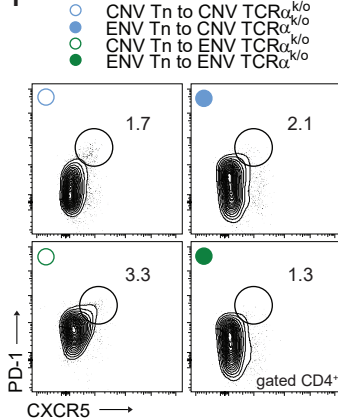

G

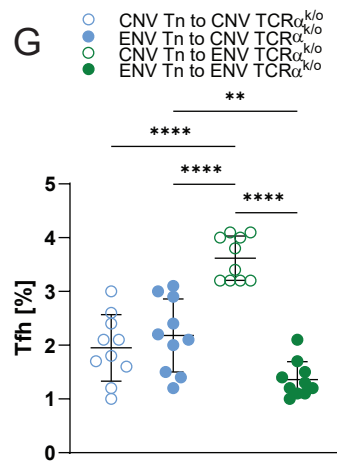

H

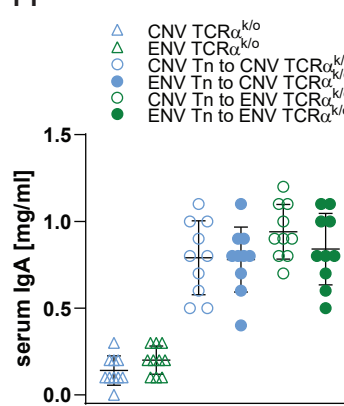

1

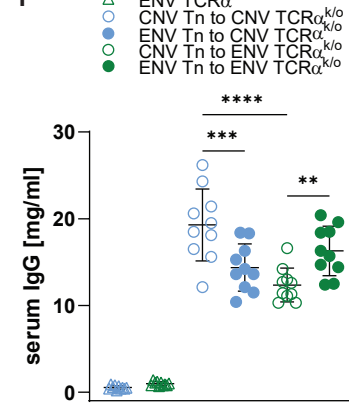

A

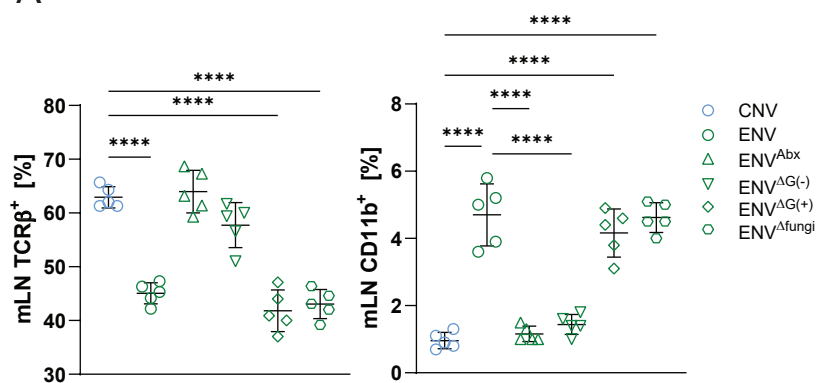

B

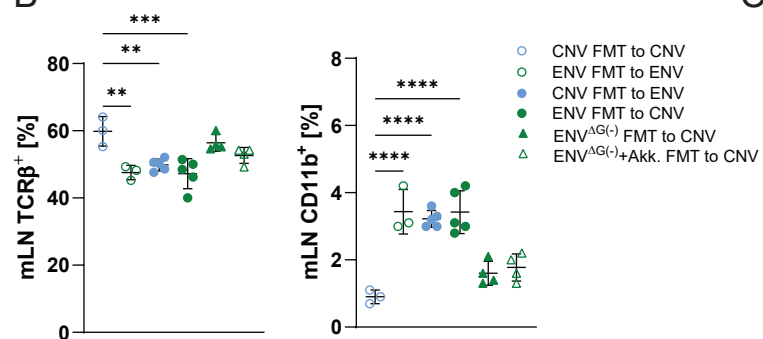

C

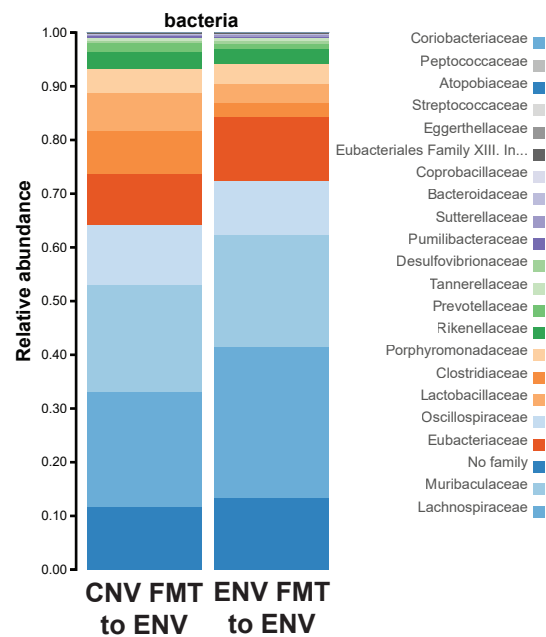

D

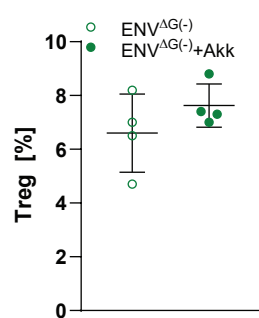

E

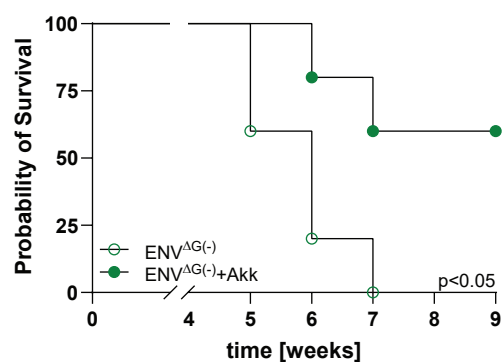

SFig. 9

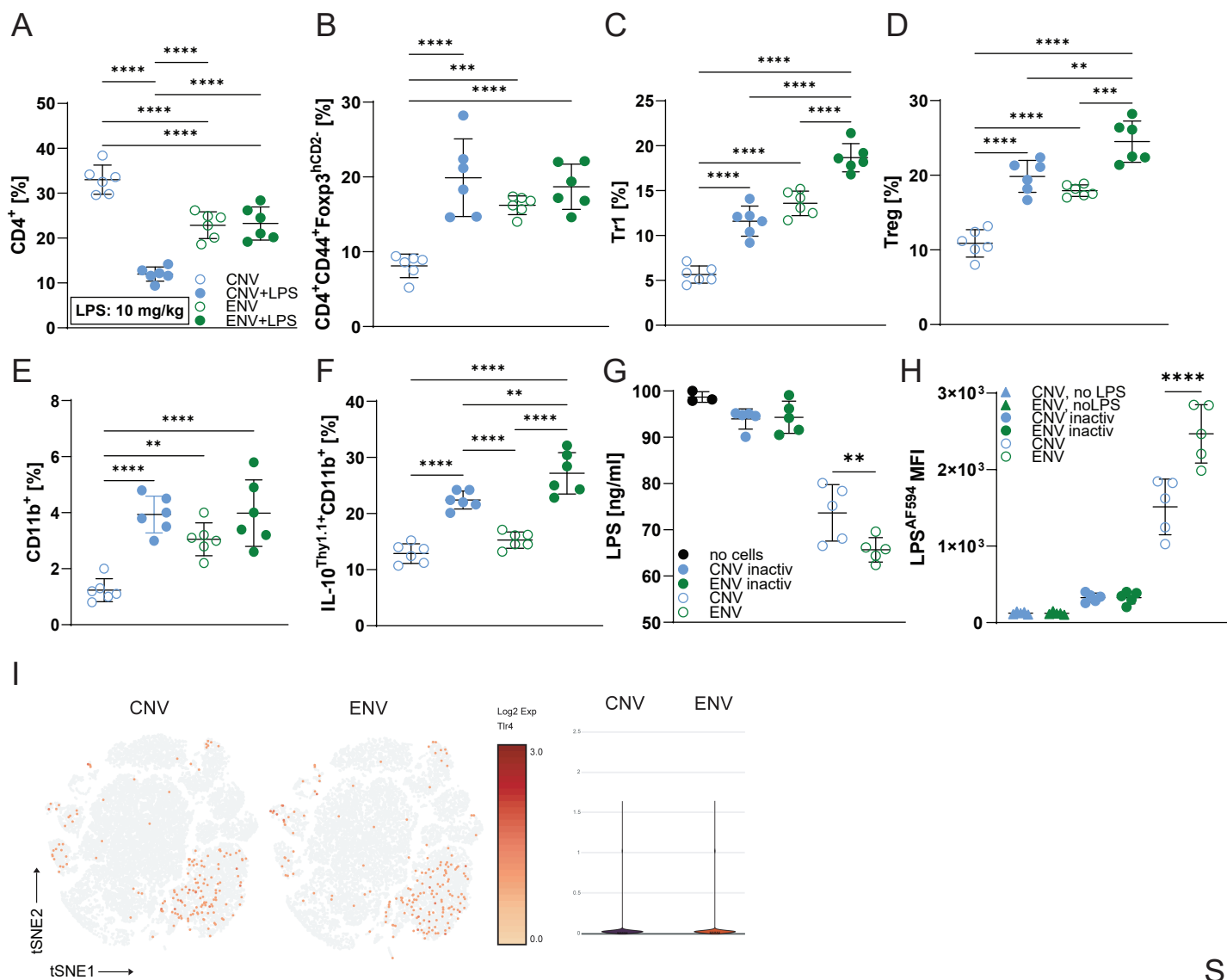

SFig. 10

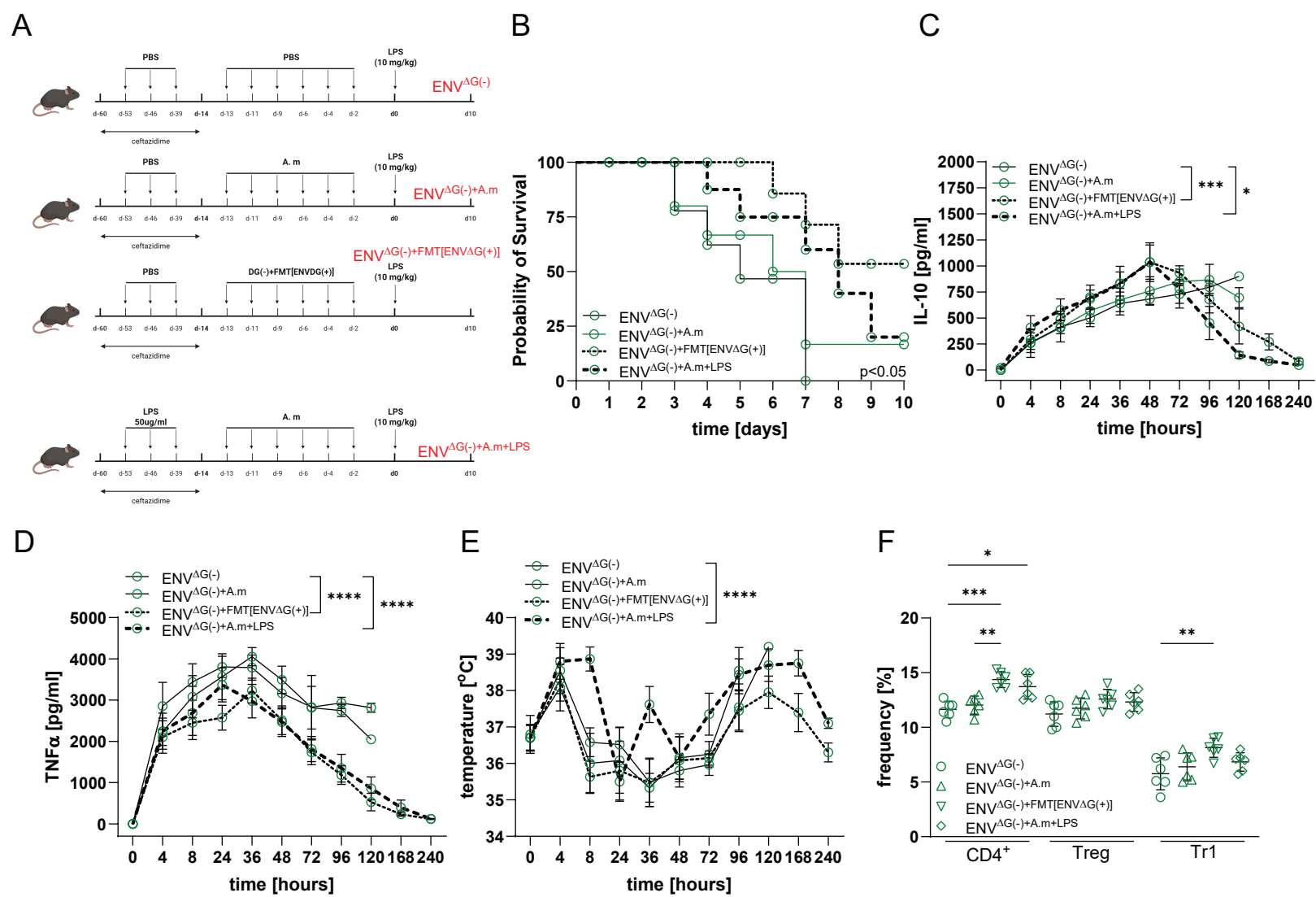

SFig. 11

Supplement: Supplementary Material — Suppl Figs_GM_resubmission.pdf [file KGMI_A_2675089_SM9168.pdf]
